# Novel Resveratrol Derivatives as Dual PDE4 Inhibitors and Free Radical Scavengers: Rational Design, Synthesis, and Biological Evaluation

**DOI:** 10.3390/antiox15070899

**Published:** 2026-07-20

**Authors:** Youzhi Wang, Huizhen Shen, Ying Liang, Guoqing Yang, Botao Zhang, Yunbao Zhi, Jinxin Wang

**Affiliations:** 1 Jiangsu Key Laboratory of Drug Design and Optimization, Department of Medicinal Chemistry, School of Pharmacy, China Pharmaceutical University, Nanjing 210009, China; 2 Institute of Traditional Chinese Medicine, Wuxi Affiliated Hospital of Nanjing University of Chinese Medicine, Wuxi 214071, China

**Keywords:** resveratrol, PDE4 inhibitor, free radical scavenger, COPD

## Abstract

A novel class of resveratrol derivatives as dual PDE4 (Phosphodiesterase 4) inhibitors and free radical scavengers for the treatment of COPD (chronic obstructive pulmonary disease) was developed in this study. In vitro, the most promising compound **WYZ69** showed similar PDE4 inhibitory activity and anti-inflammatory activity to rolipram and better DPPH free radical scavenging ability and anti-lipid peroxidative activity than edaravone. In addition, compound **WYZ69** turned out to be a novel ferroptosis inhibitor, which significantly inhibited ferroptosis as a free radical scavenger, decreased the levels of ROS (reactive oxygen species), increased the expression of GPX4 (Glutathione peroxidase 4), and decreased the expression of transferrin in A549 cells induced by a ferroptosis activator, RSL3 ((1S,3R)-RSL3). In vivo, it exhibited a half-life of 1.63 h in mice and moderate anti-inflammatory activity in mice induced by LPS (Lipopolysaccharide). Hence, these findings suggest that compound **WYZ69** is a promising dual PDE4 inhibitor and free radical scavenger with anti-inflammatory and anti-ferroptosis activities for the treatment of COPD.

## 1. Introduction

COPD (chronic obstructive pulmonary disease) is a common lung disease closely associated with inflammation and oxidative stress [1,2]. It is difficult to achieve an ideal treatment effect for COPD with bronchodilators, anti-inflammatory drugs and antioxidants alone in clinical practice [3,4]. So, the combination of anti-inflammatory drugs [5,6,7] and antioxidants [8] with bronchodilators, including β_2_ agonists and M_3_ antagonists, for COPD is common and advantageous in clinical practice [5,6,7,8,9].

Moreover, bifunctional compounds for COPD, such as dual anti-inflammatory agents/bronchodilators [10,11], have become a hot direction for the research and development of new drugs for COPD. PDE4 (Phosphodiesterase 4) inhibitors are currently an important targeted anti-inflammatory drug in the clinical treatment of COPD. The current bifunctional compounds for COPD are mainly focused on PDE4 inhibitors conjugated with *β*_2_ agonists or M_3_ antagonists [10,11].

Oxidative stress and inflammation are the two main pathological bases of COPD, and they can reinforce each other through signaling pathways [12,13,14,15,16]. On the one hand, ROS (reactive oxygen species) generated by NOXs (NADPH oxidase) are activated by several cytokines, and on the other hand, ROS activate multiple immunological activators such as IL-6 and TNF-α [14]. The main factors involved in this coupling are the transcription factors (NF-kB, AP-1 and NFAT), signaling pathways (p38, PI3K/Akt and ERK1/2) and the protein kinase C (PKC) [14]. Therefore, dual anti-inflammatory agents and antioxidants as bifunctional compounds should be superior to anti-inflammatory drugs and antioxidants alone for treating COPD. Dual PDE4 inhibitors and free radical scavengers as dual anti-inflammatory agents and antioxidants for COPD have been developed in our previous studies [17,18], but their activity and drug-like properties need to be improved.

Resveratrol is a natural plant polyphenol with PDE4 inhibition [19] and free radical scavenging activities [20]. And it has been regarded as a potential alternative therapeutic agent for COPD [21]. Therefore, this study aims to discover novel dual PDE4 inhibitors and free radical scavengers with better activity and drug-like properties from novel resveratrol derivatives for the treatment of COPD through rational design, synthesis, and biological evaluation.

## 2. Materials and Methods

### 2.1. Synthetic

The synthetic materials and procedures are as follows. All starting materials and reagents with high-grade purity were obtained from commercial sources. The ^1^H NMR and ^13^C NMR spectra were recorded on Bruker NMR spectrometers in CDCl_3_ or DMSO-d_6_. The LRMS (ESI) spectra of the synthetic intermediates were recorded on a Shimadzu LC-MS2020 mass spectrometer, and the HRMS (ESI) spectra of the synthesized target compounds were recorded on an Agilent 6230 Q-TOF mass spectrometer.

#### 2.1.1. Preparation of Benzaldehyde Intermediates **1e-1h** and **1r-1u**

3-hydroxy-4-methoxybenzaldehyde or 3-hydroxy-4-difluoromethoxy-benzaldehyde (4 mmol) and different bromo-hydrocarbons (4.8 mmol) were dissolved in DMF, added with potassium carbonate (12 mmol), and stirred at 80 °C for 12 h. The reaction mixture was diluted with ethyl acetate and washed with water. The organic phase was dried over anhydrous sodium sulfate, concentrated in vacuo and separated by silica gel column chromatography to obtain the benzaldehyde intermediates. The NMR and LRMS of the intermediates **1e-1h** have been reported in our previous study [18].

#### 2.1.2. Preparation of Styrene Intermediates **2a-2h**

The benzaldehyde intermediate (2 mmol) was dissolved in dioxane, and methyltriphenylphosphonium bromide (2.4 mmol) and potassium carbonate (3 mmol) were added and stirred at reflux temperature for 12 h [22]. The reaction mixture was diluted with ethyl acetate and washed with water. The organic phase was dried over anhydrous sodium sulfate, concentrated in vacuo and separated by silica gel column chromatography to obtain the styrene intermediates **2a-2h**.

#### 2.1.3. Preparation of Target Compounds **3a-3j**

The different bromophenol intermediates (1 mmol) and an equivalent amount of the styrene intermediates (**2a-2h**) were dissolved in triethylamine, and palladium acetate (0.04 mmol) and triphenylphosphine (0.08 mmol) were added and stirred at 80 °C for 36 h [23]. The reaction mixture was diluted with ethyl acetate and washed with water. The organic phase was combined, dried over anhydrous sodium sulfate, concentrated in vacuo and separated by silica gel column chromatography to obtain the target compounds **3a-3j**.

#### 2.1.4. Preparation of Target Compounds **4a-4h**

5-hydroxyindolin-2-one (1 mmol) and an equivalent amount of the different benzaldehyde intermediates (**1e-1h**, **1r-1u**) were dissolved in ethyl alcohol, and piperidine (0.02 mL) was added and stirred at 80 °C for 12 h [24]. The reaction mixture was diluted with ethyl acetate and washed with water. The organic phase was dried over anhydrous sodium sulfate, concentrated in vacuo and separated by silica gel column chromatography to obtain the target compounds **4a-4h**.

### 2.2. PDE4 Inhibition Activity Assay

The inhibition activity of the compounds on the PDE4 enzyme was tested using the IMAP FP Explorer Kit (Molecular Devices, San Jose, CA, USA). The buffers containing PDE4B1 (BPS bioscience, San Diego, CA, USA) and without PDE4B1 were added to the plates containing the DMSO solutions of the test compounds as the compound groups and blank groups, respectively. The buffers containing PDE4B1 were added to the plates without the test compounds as the PDE4 groups. All the plates were incubated at room temperature for 15 min. Then, the FAM-cAMP substrate solution was added and incubated at room temperature for 60 min. Finally, termination buffer was added and incubated at room temperature for 1 h. The fluorescence intensity (Ex. 485 nm, Em. 530 nm) was detected using a PerkinElmer Multifunctional enzyme label, and the inhibition rate (%) of PDE4 activity of the test compounds was calculated as the percentage of (FLU_PDE4_ − FLU_compound_)/(FLU_PDE4_ − FLU_blank_). The values and curves of IC_50_ for the test compounds were calculated and fitted using GraphPad Prism 8 software.

### 2.3. DPPH Radical Scavenging Activity Assay

The assay method for DPPH radical scavenging activity of the compounds was performed as described in our previous report [17]. The values and curves of IC_50_ for the test compounds were calculated and fitted using Prism GraphPad software.

### 2.4. Cytotoxicity and Anti-Inflammatory Effects Assay in RAW 264.7 Cells Induced by LPS

RAW264.7 cells were obtained from the Chinese Academy of Science Cell Bank (Shanghai, China) and were cultured at 37 °C with 5% CO_2_ in DMEM containing 10% fetal bovine serum (Gibco, New York, NY, USA), streptomycin_2_ (100 mg/mL) and penicillin (100 U/mL). Cells were seeded into 96-well plates at 2 × 10^4^ cells/well and cultured for 12 h. Then, the growth medium was replaced with differentiation mediums containing the stimulant rolipram (10 μM), test compounds (10 μM) or vehicle (0.1% DMSO). One hour later, the cells pretreated with the compounds were induced with LPS (10 ng/mL) as the compound groups, the cells without compounds were induced with LPS (10 ng/mL) as the LPS groups, and the cells without compounds and LPS were used as the control groups. After 12 h, the growth mediums were collected and centrifuged (3500 rpm, 10 min, 4 °C) to obtain the supernatant, which was stored at −80 °C for testing TNF-α levels using an ELISA kit (Fine Biotech, Wuhan, China) according to the manufacturer’s instructions. Optical density (OD) measurements were taken at a wavelength of 450 nm with a microplate photometer (Thermo Scientific, Waltham, MA, USA). The % inhibition of TNF-α was calculated as the percentage of (OD_LPS_ − OD_compound_)/(OD_LPS_ − OD_control_). A total of 10 μL of CCK8 (Beyotime, Shanghai, China) was added to each well with cells and incubated for 1 h at 37 °C in 5% CO_2_ after the growth medium was collected and replaced. OD measurements were taken at a wavelength of 450 nm with a Thermo Scientific Multiskan FC microplate photometer.

### 2.5. Anti-Lipid Peroxidation Effect in Fe^2+^-Induced Mouse Lung Homogenates

Mice were killed, and their lungs were collected to prepare 10% lung tissue homogenate with saline solution. A total of 100 μL of saline solution (2.5% DMSO and 5% Tween 80) containing the test compounds **WYZ69** or edaravone (2000, 1000 and 500 μM) was added to 100 μL of 10% lung tissue homogenate containing FeCl_2_ (1 mM), which was used as a test sample (1000, 500 and 250 μM). A total of 100 μL of saline solution (2.5% DMSO and 5% Tween 80) was added to 10% lung tissue homogenate containing FeCl_2_ (1 mM), which was used as a Fe^2+^ control sample. A total of 100 μL of saline solution (2.5% DMSO and 5% Tween 80) was added to 10% lung tissue homogenate, which was used as a blank control sample. MDA levels in the samples were detected using an MDA kit (Nanjing Jiancheng, Nanjing, China) after incubation at 37 °C for 1 h in the dark. The reaction reagents of the kit were added to the samples and incubated at 90 °C for 1 h in the dark. The mixtures were cooled to room temperature and then added to a 96-well plate. OD measurements were taken at a wavelength of 545 nm with a Thermo Scientific Multiskan FC microplate photometer. The inhibition rate (%) of MDA levels was calculated as the percentage of (OD_Fe_ − OD_test_)/(OD_Fe_ − OD_blank_).

### 2.6. Anti-Ferroptosis Effect in A549 Induced by RSL3

#### 2.6.1. Cytotoxicity and Cell Viability Assay

A549 cells were obtained from the Chinese Academy of Science Cell Bank (Shanghai, China) and cultured at 37 °C with 5% CO_2_ in DMEM containing 10% fetal bovine serum (Gibco, USA). Cells were seeded (0.2 × 10^4^ cells/well) in a 96-well plate and cultured for 24 h. Then, the culture mediums of the cells were replaced with fresh culture mediums or differentiation culture mediums containing Fer-1 (5 μM, 0.1% DMSO) or **WYZ69** (5 μM and 10 μM, 0.1% DMSO), and the cells were stimulated with RSL3 (5 μM, 0.1% DMSO) or vehicle (0.2% DMSO) for 72 h. A total of 10 μL of CCK8 (Beyotime, Shanghai, China) was added to each well with cells and incubated for 1 h at 37 °C in 5% CO_2_ after the growth medium was collected and replaced. OD measurements were taken at a wavelength of 450 nm with a Thermo Scientific Multiskan FC microplate photometer.

#### 2.6.2. Intracellular ROS Levels Assay

A549 cells were plated (4 × 10^4^ cells/well) in a 6-well plate and incubated for 12 h. Then, the culture mediums of the cells were replaced with fresh culture mediums or differentiation culture mediums containing Fer-1 (5 μM, 0.1% DMSO) or **WYZ69** (10 μM, 0.1% DMSO), and RSL3 (5 μM, 0.1% DMSO) or vehicle (0.2% DMSO) was added to the cells for 72 h. After that, the intracellular ROS levels were quantified by measuring the fluorescence of DCFH-DA (Beyotime, Shanghai, China). The cells were washed with culture mediums without FBS and incubated with DCFH-DA at 37 °C for 20 min. The cells were washed three times with fresh culture mediums without FBS. Finally, fluorescence signals were captured using an Olympus CFX53 fluorescence microscope with excitation/emission wavelengths of 488/522 nm. The mean fluorescence intensity was quantified using Image J software 1.47v (National Institutes of Health, Bethesda, MD, USA).

#### 2.6.3. Western Blot Assay

A549 cells were plated (4 × 10^4^ cells/well) in a 6-well plate and incubated for 12 h. Then, the culture mediums of the cells were replaced with fresh culture mediums or the culture mediums containing Fer-1 (5 μM, 0.1% DMSO) or **WYZ69** (10 μM, 0.1% DMSO), and the cells were stimulated with RSL3 (5 μM, 0.1% DMSO) or vehicle (0.2% DMSO) for 72 h. After that, the cells were collected, and total proteins were extracted from cells. The BCA assay kit (Beyotime, Shanghai, China) was used to evaluate protein concentration. Sodium dodecyl sulfate–polyacrylamide gel electrophoresis (SDS-PAGE) was applied to separate 20 μg proteins of each sample. The proteins were transferred to poly-vinylidene fluoride (PVDF) membranes (Merck Millipore, Rahway, NJ, USA). Subseque, USA, membranes were incubated with primary antibodies at 4 °C overnight after being blocked in 5% skim milk for 1 h at 37 °C. The primary antibodies include anti-GPX4 (1:1000, Abclonal, Shanghai, China), anti-transferrin (1:1500, Abclonal, Shanghai, China), and HRP Conjugate anti-GAPDH (1:5000, CST, USA). The membrDanvers, MA, anes for anti-GPX4 and anti-transferrin-incubated membranes were incubated with goat anti-rabbit IgG, HRP-linked antibody (1:6000, CST, USA) for 2 h atDanvers, MA, room temperature. Finally, protein signals were captured using the Tanon-4600 image system (Tanon, Shanghai, China). The band intensities were quantified by Image J software 1.47v (National Institutes of Health, Bethesda, MD, USA).

### 2.7. Animals

Male ICR mice (18–22 g) were obtained from Hangzhou Medical College (Hangzhou, China).

### 2.8. Pharmacokinetic Study in Mice

The pharmacokinetic study of compound **WYZ69** (1 mg/kg, i.v) in mice was studied, and the method was performed as described in our previous report [25].

### 2.9. Anti-Inflammatory Effect on LPS-Induced Mice

Anti-inflammatory effects on LPS-induced acute inflammation in mice of compound **WYZ69** (10 mg/kg and 30 mg/kg, i.p.) were studied, and the method was described in a previous report [17]. The TNF-α levels in serum were measured using an ELISA kit (Fine Biotech, Wuhan, China) according to the manufacturer’s instructions. OD measurements were taken at a wavelength of 450 nm with a Thermo Scientific Multiskan FC microplate photometer. The inhibition rate (%) of TNF-α levels was calculated as the percentage of (OD_LPS_ − OD_test_)/(OD_LPS_ − OD_control_).

### 2.10. Molecular Docking In Silico

Molecular docking in silico was performed using the same procedures as in our previous report [17].

### 2.11. Statistical Analysis

Data were expressed as the means ± SD, and *p* < 0.05 was considered statistically significant. All data from different groups were analyzed using Prism GraphPad software. Comparisons among multiple groups were conducted using one-way ANOVA, and pairwise comparisons between groups were performed using the LSD test. The comparisons between two groups alone required the Student’s *t*-test.

## 3. Results

### 3.1. Compound Design

**Figure 1:**
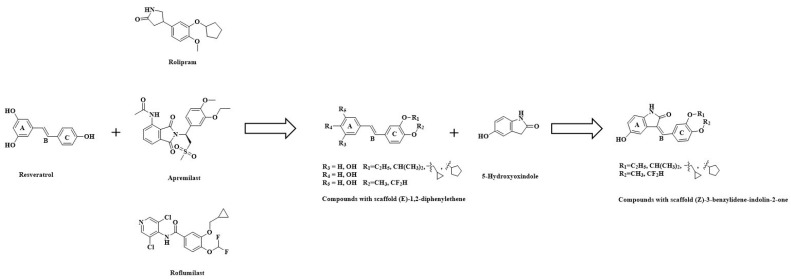
The rational design strategy of novel resveratrol derivatives as dual PDE4 inhibitors and free radical scavengers.

The overall design idea of developing better bifunctional compounds in this study is shown in Figure 1. Firstly, resveratrol was used as the lead compound and hybridized with PDE4 inhibitors such as rolipram, apremilast and roflumilast to improve its PDE4 inhibitory activity, resulting in a series of target bifunctional compounds with the scaffold (E)-1,2-diphenylethene. Subsequently, they were further hybridized with 5-Hydroxyoxindole, an excellent natural free radical scavenger [26], to improve their free radical scavenging activity, resulting in a series of target bifunctional compounds with the scaffold (Z)-3-benzylidene-indolin-2-one.

### 3.2. Chemistry

**Scheme 1:**
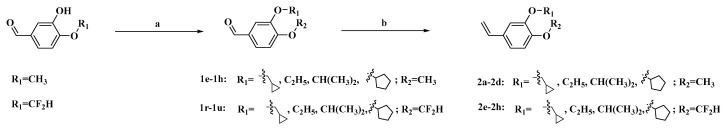
Reagents and conditions: (a) RBr, K_2_CO_3_, DMF, 80 °C, 12 h. (b) Methyltriphenylphosphonium bromide, K_2_CO_3_, dioxane, reflux, 12 h.

**Scheme 2:**
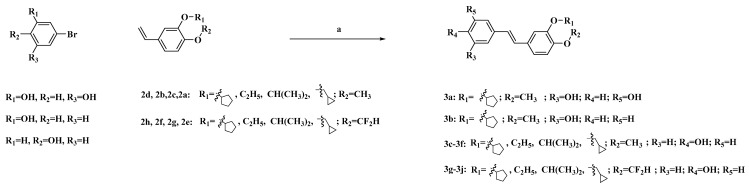
Reagents and conditions: (a) Tris-(2-methylphenyl)-phosphine, Pd (OAc)_2_, triethylamine, 80 °C, 36 h.

**Scheme 3:**
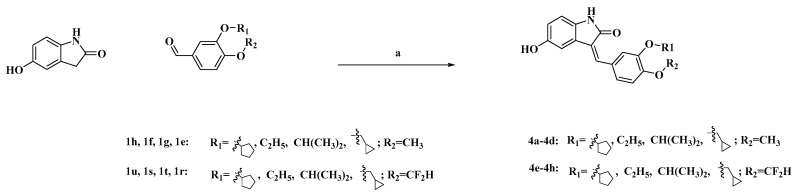
Reagents and conditions: (a) piperidine, ethanol, 80 °C, 12 h.

The benzaldehyde intermediates (**1e-1h**, **1r-1u)** were synthesized by 3-hydroxy-4-methoxybenzaldehyde or 3-hydroxy-4-difluoromethoxy-benzaldehyde (4 mmol) and different bromo-hydrocarbons (4.8 mmol) with potassium carbonate (12 mmol) in DMF at 80 °C for 12 h (Scheme 1). These intermediates were then reacted with methyltriphenylphosphonium bromide and K_2_CO_3_ in refluxed dioxane for 12 h to give the styrene intermediates (**2a-2d, 2e-2h**) (Scheme 1). The target compounds **3a-3j** with the scaffold (E)-1,2-diphenylethene were obtained from different bromobenzene raw materials and the styrene intermediates with tris-(2-methylphenyl)-phosphine and Pd (OAc)_2_ in triethylamine at 80 °C for 36 h (Scheme 2). The target compounds **4a-4h** with the scaffold (Z)-3-benzylidene-indolin-2-one were obtained by 5-hydroxyindolin-2-one and the benzaldehyde intermediates with piperidine in ethanol at 80 °C for 12 h (Scheme 3).

### 3.3. Screening for Dual PDE4 Inhibitors and Free Radical Scavengers

To verify the feasibility of rationally designing dual PDE4 inhibitors and free radical scavengers through the molecular hybridization strategy in Figure 1, the first batch of compounds **3a-3c** with (E)-1,2-diphenylethene as a scaffold and **4a** with (Z)-3-benzylidene-indolin-2-one as a scaffold were prepared and tested. The structures and dual activities against PDE4 and DPPH (2, 2-diphenyl-1-picrylhydrazyl) free radicals of those compounds, control PDE4 inhibitor rolipram, and control free radical scavenger edaravone are shown in Table 1. Most of the compounds possess dual PDE4 inhibition and free radical-scavenging activities. Among them, the derivative of resveratrol, **4a**, exhibited significantly enhanced PDE4 inhibitory activity and free radical scavenging activity compared to resveratrol. It means that the molecular hybridization strategy worked. Compound **3c** with the best inhibitory activity of PDE4 and compound **4a** with the best free radical scavenging activity were selected as the lead compounds with the skeletons of (E)-1,2-diphenylethene and (Z)-3-benzylidene-indolin-2-one for subsequent optimization.

To further optimize the PDE4 inhibition activity and drug-like properties of the lead compounds **3c** and **4a**, they were further molecularly hybridized with PDE4 inhibitors and derivatized. By replacing R_1_ and R_2_ of the lead compounds **3c** and **4a** with the pharmacological groups of the PDE4 inhibitors in Figure 1, the second batch of compounds was prepared and tested. As shown in Table 2, all the compounds possess good dual PDE4 inhibition and free radical scavenging activities and the compounds **3d**, **3h**, **4b** and **4f** with R_1_ as an ethyl group have better PDE4 inhibition activity than the others. Furthermore, according to the classic lead compound optimization strategy, compounds containing fluorine may have better in vivo metabolic stability.

According to the PDE4 inhibitory activities of the compounds in Table 2, the compounds **3d**, **3h**, **4b** and **4f** with R_1_ as an ethyl group were selected as candidate compounds for IC_50_ tests of PDE4 and DPPH in vitro. As shown in Table 3, the compounds **3h** and **4f** with R_2_ as a difluoromethyl group possess better PDE4 inhibition activity than the compounds **3d** and **4b** with R_2_ as a methyl group. The compounds **4b** and **4f** with the skeletons of (Z)-3-benzylidene-indolin-2-one have better free radical scavenging activity than the compounds **3d** and **3h** with the skeletons of (E)-1,2-diphenylethene, but their PDE4 inhibition activity is slightly weaker. Among them, the derivative of resveratrol, **4f**, exhibited significantly enhanced PDE4 inhibitory activity and free radical scavenging activity compared to resveratrol.

### 3.4. Anti-Inflammatory Activities of the Candidate Compounds in RAW264.7 Cells Induced by LPS

**Figure 2:**
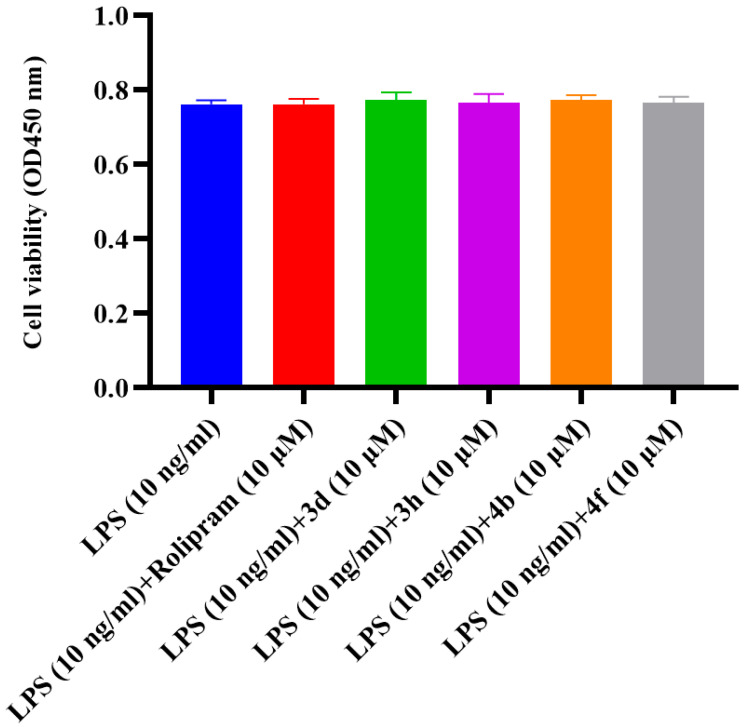
Cytotoxicity of the candidate compounds. The value is expressed as mean ± SD (*n* = 3).

**Figure 3:**
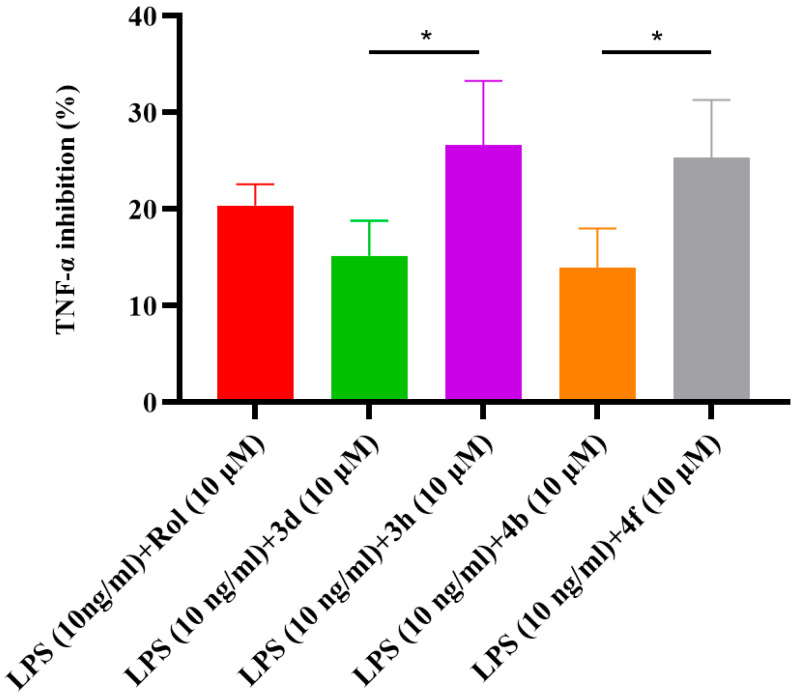
Anti-TNF-α production effects of the candidate compounds in cells. The value is expressed as mean ± SD (*n* = 3). * *p* < 0.05.

PDE4 inhibitors play an anti-inflammatory role mainly by inhibiting the production of the inflammatory cytokine TNF-α (tumor necrosis factor-alpha). RAW264.7 cells induced by LPS (Lipopolysaccharide) were used to evaluate the anti-TNF-α production effects of the candidate compounds in this study. The cytotoxicity and anti-inflammatory activities of the candidate compounds **3d**, **3h**, **4b** and **4f** in LPS-induced RAW264.7 cells are shown in Figure 2 and Figure 3, respectively. Compounds **3h** and **4f** (10 µM) with CF_2_H substitution as R_2_ possess significantly superior anti-inflammatory activity compared to compounds **3d** and **4b** (10 µM) with alkyl substitution as R_2_ and no cytotoxicity. There are no significant differences in anti-inflammatory activity among compounds **3h**, **4f** and rolipram (10 µM). Interestingly, although the PDE4 inhibitory activity of compound **4f** is not as strong as that of compound **3h**, it has similar anti-inflammatory activity to that of compound **3h**. This might be related to its strong free radical scavenging activity, which enhances its anti-inflammatory activity through the synergistic effect of antioxidant stress. Therefore, compound **4f** was selected as the candidate compound for in vitro and in vivo activity studies and was named **WYZ69** according to the initial of the inventor’s full name and the compound discovery number, as mentioned in the subsequent research.

### 3.5. Antioxidant Stress Effect of the Candidate Compounds in Fe^2+^-Induced Mouse Lung Homogenates

The lipid peroxidation induced by Fe^2+^ in mouse lung homogenates was used as an animal model of COPD in vitro to evaluate the antioxidant stress effect of compound **WYZ69,** and the levels of the lipid peroxidation product MDA were tested as the biomarker of lipid peroxidation. Edaravone was used as a free radical scavenger inhibitor control. As shown in Table 4, the inhibitory effect of **WYZ69** on MDA levels in Fe^2+^-induced mouse lung homogenates is 3 to 5 times that of edaravone. Therefore, **WYZ69** indeed possesses the same strong antioxidant stress activity as hypothesized.

### 3.6. Ferroptosis Inhibitory Effect of Compound WYZ69 in A549 Induced by RSL3

More and more studies have found that ferroptosis is closely related to the pathogenesis of COPD [27,28,29,30]. Recent studies have found that CS (cigarette smoke) mediates iron metabolism and iron-regulated ROS (reactive oxygen species) production and ferroptosis in COPD [30,31,32]. According to the mechanism of ferroptosis, free radical scavengers are one type of ferroptosis inhibitors [33,34].

**Figure 4:**
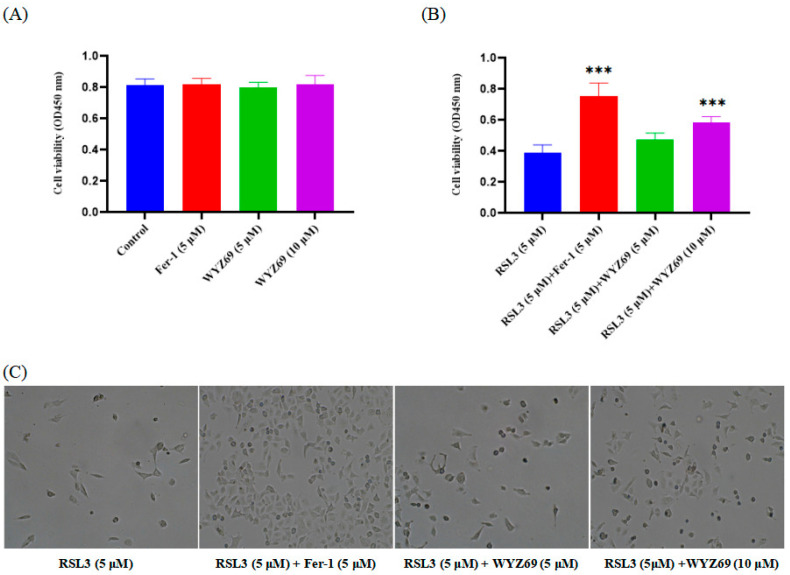
Cytotoxicity and anti-ferroptosis effect of compound **WYZ69** in A549 cells induced by RSL3: (**A**) Cytotoxicity of compound **WYZ69** (5 µM and 10 µM) in A549 cells compared with Fer-1 (5 µM). (**B**) Effect of compound **WYZ69** on cell viability of A549 cells treated with 5 µM RSL3 in the absence or presence of Fer-1 (5 µM) or compound **WYZ69** (5 µM and 10 µM). (**C**) Morphology of A549 cells in different groups under a microscope. The value is expressed as mean ± SD (n = 3). *** *p* < 0.001 compared to the RSL3 group.

**Figure 5:**
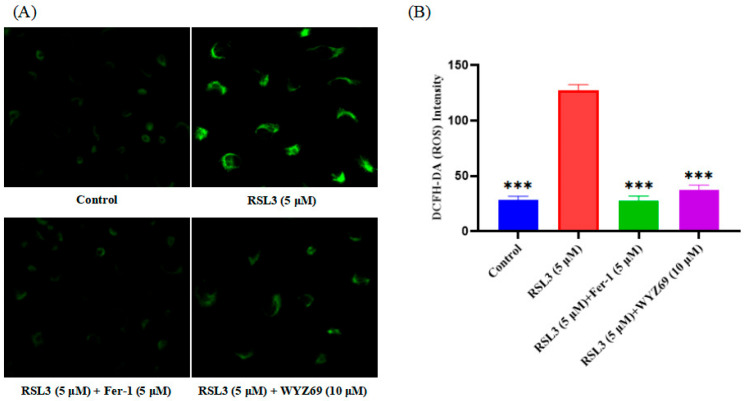
Effect on the intracellular ROS level of compound **WYZ69** in A549 cells induced by RSL3: (**A**) The confocal image of the fluorescence of DCFH-DA in A549 cells untreated and treated with 5 µM RSL3 in the absence or presence of Fer-1 (5 µM) or compound **WYZ69** (10 µM). (**B**) DCFH-DA fluorescence intensity of the A549 cells in different groups. The value is expressed as mean ± SD (*n* = 3). *** *p* < 0.001 compared to the RSL3 group.

Therefore, the ROS free radical scavenging and anti-ferroptosis activities of compound **WYZ69** were tested. As shown in Figure 4, compound **WYZ69** (10 µM) possesses similar inhibitory activity against the GPX4 covalent inhibitor RSL3 ((1S,3R)-RSL3)-induced ferroptosis in A549 cells to the control ferroptosis inhibitor Fer-1 (5 µM) without cytotoxicity. As shown in Figure 5, compound **WYZ69** (10 µM) exhibits excellent intracellular ROS radical scavenging activity in A549 cells induced by RSL3.

**Figure 6:**
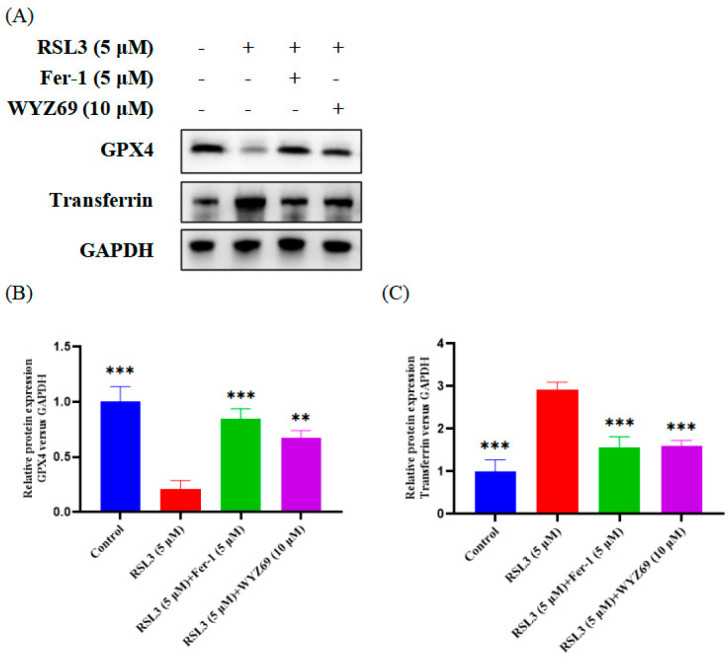
Effect on the expression of GPX4 and transferrin of compound **WYZ69** in A549 cells induced by RSL3: (**A**) Western blotting analyses of GPX4 and transferrin of A549 cells untreated and treated with 5 µM RSL3 in the absence or presence of Fer-1 (5 µM) or compound **WYZ69** (10 µM). (**B**) Quantification of the Western blotting analyses of GPX4 in different groups. (**C**) Quantification of the Western blotting analyses of transferrin in different groups. The value is expressed as mean ± SD (*n* = 3). ** *p* < 0.01 and *** *p* < 0.001 compared to the RSL3 group.

The expression level of GPX4 in the death cells induced by RSL3 via the ferroptosis pathway decreases, while the expression level of transferrin increases, and it can be reversed by the ferroptosis inhibitor Fer-1 [35]. As shown in Figure 6, compound **WYZ69** (10 µM) can also significantly increase the expression of GPX4 and reduce the expression of transferrin in A549 induced by RSL3 and is similar to Fer-1. Hence, these findings suggest that compound **WYZ69** is a potent ferroptosis inhibitor that acts as a ROS free radical scavenger for the treatment of COPD. Although the compound may exert anti-ferroptosis effects by scavenging free radicals, we are not sure whether the products generated by its reaction with ROS have PDE4 inhibitory activity. This requires further research in the future.

### 3.7. Pharmacokinetics of Compound WYZ69 in Mice

The pharmacokinetic properties of compound **WYZ69** were studied in mice. As shown in Table 5, the terminal half-life (T_1/2_) of intravenous administration of **WYZ69** (1 mg/kg) is 1.63 h, and its clearance (CL) is 874.35 mL/(kg·h). The terminal half-life of 1.63 h is acceptable for sustained-release formulations and inhalation preparations, but it is insufficient for oral generic preparations.

### 3.8. Anti-Inflammatory Effect of Compound WYZ69 in LPS-Induced Mice

**Figure 7:**
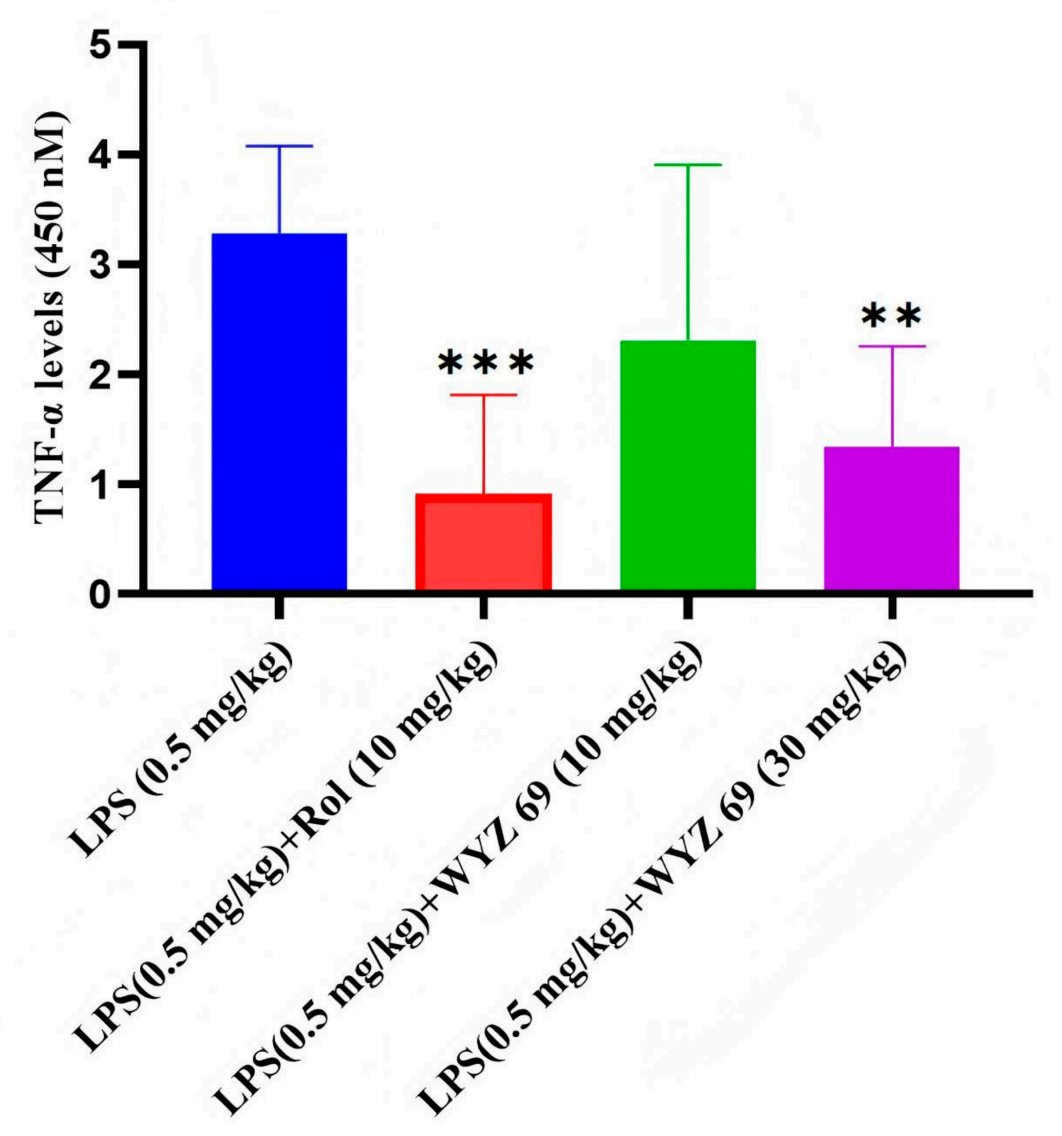
The value is expressed as mean ± SD (n = 8). ** *p* < 0.01 and *** *p* < 0.001 compared to the LPS group.

The mice induced by intratracheal injection of LPS were adopted as an animal model of COPD in vivo to evaluate the anti-inflammatory effect of compound **WYZ69**. As shown in Figure 7 and Table 6, serum levels of TNF-α in mice (n = 8) as the biomarker of inflammation were significantly inhibited by compound **WYZ69** (30 mg/kg, i.p.).

### 3.9. Molecular Docking of WYZ69 In Silico

**Figure 8:**
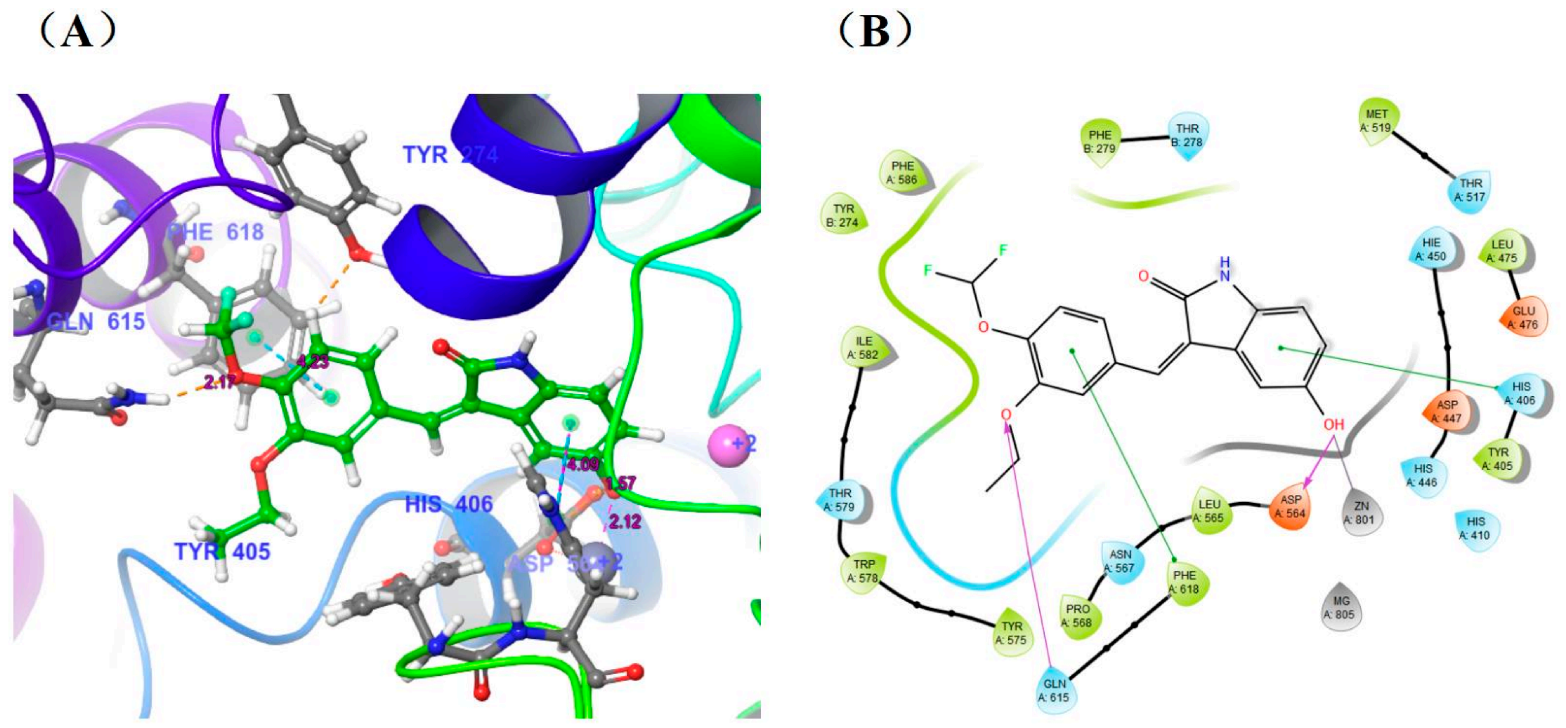
Diagram of the molecular docking: (**A**) 3D diagram of **WYZ69**/4X0F. (**B**) 2D diagram of **WYZ69**/4X0F.

The molecular docking of compound **WYZ69** with PDE4 (4X0F) is shown in Figure 8. The group -OC_2_H_5_ of compound **4f** occupies small hydrophobic pockets of PDE4 binding sites, and its benzene ring forms a π-π interaction with PHE618 (4.23 Å). The group -OCF_2_H of compound **4f** not only occupies large pockets of PDE4 binding sites but also its oxygen atom forms an H-bond with GLN615 (2.17 Å). Its hydrophilic terminal phenol hydroxyl occupies the metal chelating region, its hydroxy forms an H-bond with ASP564 (1.67 Å), its benzene ring forms a π-π interaction with HIS406 (4.09 Å), and its oxygen atom forms an electrostatic interaction with Zn801 (2.12 Å) within the metal chelating region of PDE4 binding sites.

## 4. Discussion

Resveratrol is a natural compound with dual functions of anti-inflammation and antioxidation. It can exert anti-inflammatory effects by inhibiting PDE4 and antioxidant effects by eliminating ROS. However, its inhibitory activity on PDE4 and its ROS-eliminating activity are both very weak. The multiple phenolic hydroxyl groups in its molecule result in poor pharmacological properties. In this study, it was molecularly hybridized with the marketed PDE4 inhibitors and excellent free radical scavengers, and compound **WYZ69** with better activity and pharmacological properties than resveratrol, was obtained. **WYZ69** has shown potential for use in treating COPD in both in vitro and in vivo studies. However, its elimination half-life in mice is relatively short, making it unsuitable for development as an oral immediate-release formulation. Inhalation preparations and sustained-release formulations will be our development directions for **WYZ69** in the application of treating COPD.

**WYZ69** is a dual PDE4 inhibitor and free radical scavenger that we have developed for the treatment of COPD. The main purpose of its molecular design is to enhance the free radical clearance function of the PDE4 inhibitor in order to improve the therapeutic effect against COPD. According to the pathogenesis of COPD, inflammation and oxidative stress are the two main pathological features. **WYZ69**, through the inhibition of PDE4 and the clearance of ROS, simultaneously inhibits inflammation and oxidative stress in COPD patients and may produce better therapeutic effects than the currently available PDE4 inhibitors. Moreover, the free radical clearance activity of the current clinical antioxidants for COPD, such as NAC, is very weak, and there is little significance in combining them with PDE4 inhibitors. The free radical clearance activity of **WYZ69** is comparable to that of the currently available free radical clearance agent edaravone, and even as an antioxidant for treating COPD, it has research and application value.

Although **WYZ69** demonstrated potential for anti-COPD application in this study, some limitations of this study need to be mentioned. In this study of PDE4 inhibitory activity, we did not test the A-D isoform selectivity of the PDE4 inhibitory activity of **WYZ69**. This is because its molecular structure was obtained by hybridizing resveratrol with non-selective PDE4 inhibitor molecules. It is expected that **WYZ69** is a non-subtype-selective PDE4 inhibitor.

In this research on the anti-COPD effect, we combined the induction of acute pneumonia in mice by intratracheal injection of LPS and the lipid peroxidation model of mouse lung tissue homogenate induced by Fe^2+^ in vitro to evaluate the potential of **WYZ69** to exert anti-COPD effects through dual anti-inflammatory and antioxidant functions. However, there is a lack of direct efficacy studies of **WYZ69** on COPD model animals. The dosage selection of the positive drugs rolipram and **WYZ69** takes into account the research purpose, the research basis, the half-life of **WYZ69**, and the acute toxicity.

Based on our previous research foundation [17], the positive drug rolipram at a dose of 10 mg/kg showed a significant inhibitory effect on inflammation in the LPS-induced model mice. To conduct a head-to-head efficacy comparison between compound **WYZ69** and the positive drug, we used the same dose of 10 mg/kg. Considering that the half-life of compound **WYZ69** is shorter than rolipram, we also added a high dose of 30 mg/kg without acute toxicity.

In this study of anti-ferroptosis effects, we used RSL3 to induce a ferroptosis cell model in A549 cells. We employed ROS levels and cell proliferation ability, as well as the expression levels of GPX4 and transferrin, as the efficacy indicators for evaluating the anti-ferroptosis effects of the drug **WYZ69** in eliminating intracellular ROS. Although in many studies, A549 cells were used in studies related to COPD [36,37,38,39,40], it is possible that A549, as a human lung adenocarcinoma epithelial cell, does not fully represent the airway epithelium cell in COPD. Whether **WYZ69** indirectly increases the expression level of GPX4 by eliminating ROS or directly increases it through other mechanisms needs to be proven in our subsequent research.

## 5. Conclusions

In this study, novel series of resveratrol derivatives were designed and synthesized to develop better dual PDE4 inhibitors and free radical scavengers for the treatment of COPD. As a result, **WYZ69** was discovered as the best candidate compound for COPD in this work, which showed excellent DPPH scavenging activity (IC_50_ = 35.7 µM), moderate PDE4 inhibitory activity (IC_50_ = 469 nM), similar anti-inflammatory activity to that of rolipram in LPS-treated RAW 264.7 cells, and better anti-lipid peroxidation activity than edaravone in vitro. In addition, it significantly decreased the levels of ROS, increased the expression of GPX4, and decreased the expression of transferrin in A549 cells induced by RSL3, which showed a significant anti-ferroptosis effect as a free radical scavenger. Compound **WYZ69** showed a half-life of 1.63 h in mice (1 mg/kg, i.v.) and moderate anti-inflammatory activity in mice. Hence, compound **WYZ69** is a promising dual PDE4 inhibitor and free radical scavenger with anti-inflammatory and anti-ferroptosis activities, which, as a bifunctional compound, may provide a new therapeutic strategy for the treatment of COPD.

## Figures and Tables

**Table 1:** Dual-target inhibition activity of the first batch of compounds.

NO	Structure	Inhibition Rate (%)	
		PDE4/10 µM	DPPH/50 µM
Resveratrol	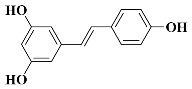	56.0 ± 1.0	42.3 ± 0.7
**3a** *	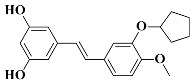	72.7 ± 1.4	6.8 ± 0.9
**3b** *	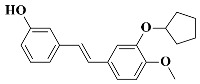	54.1 ± 0.7	NA
**3c** *	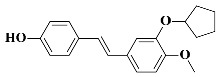	97.0 ± 1.5	30.0 ± 0.6
**4a** *	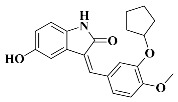	88.0 ± 0.8	69.8 ± 0.1
Rolipram	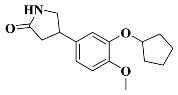	99.8 ± 0.3	ND
Edaravone	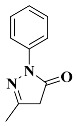	ND	63.4 ± 1.0

The value is expressed as mean ± SD (*n* = 2). *: new molecular entity; NA: not active (inhibition rate < 5%); ND: not determined.

**Table 2:** Dual-target inhibition activity of the second batch of compounds.

NO	Structure	Inhibition Rate (%)	
		PDE4/1 µM	DPPH/50 µM
Resveratrol	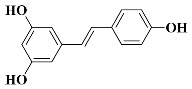	32.0 ± 1.3	43.1 ± 0.8
**3c** *	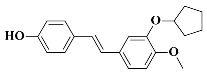	67.3 ± 1.6	31.3 ± 1.4
**3d** *	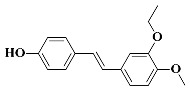	80.4 ± 1.9	35.7 ± 2.8
**3e** *	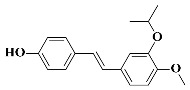	64.9 ± 1.7	42.3 ± 1.2
**3f** *	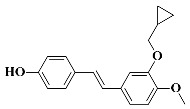	66.3 ± 1.5	31.7 ± 1.6
**3g** *	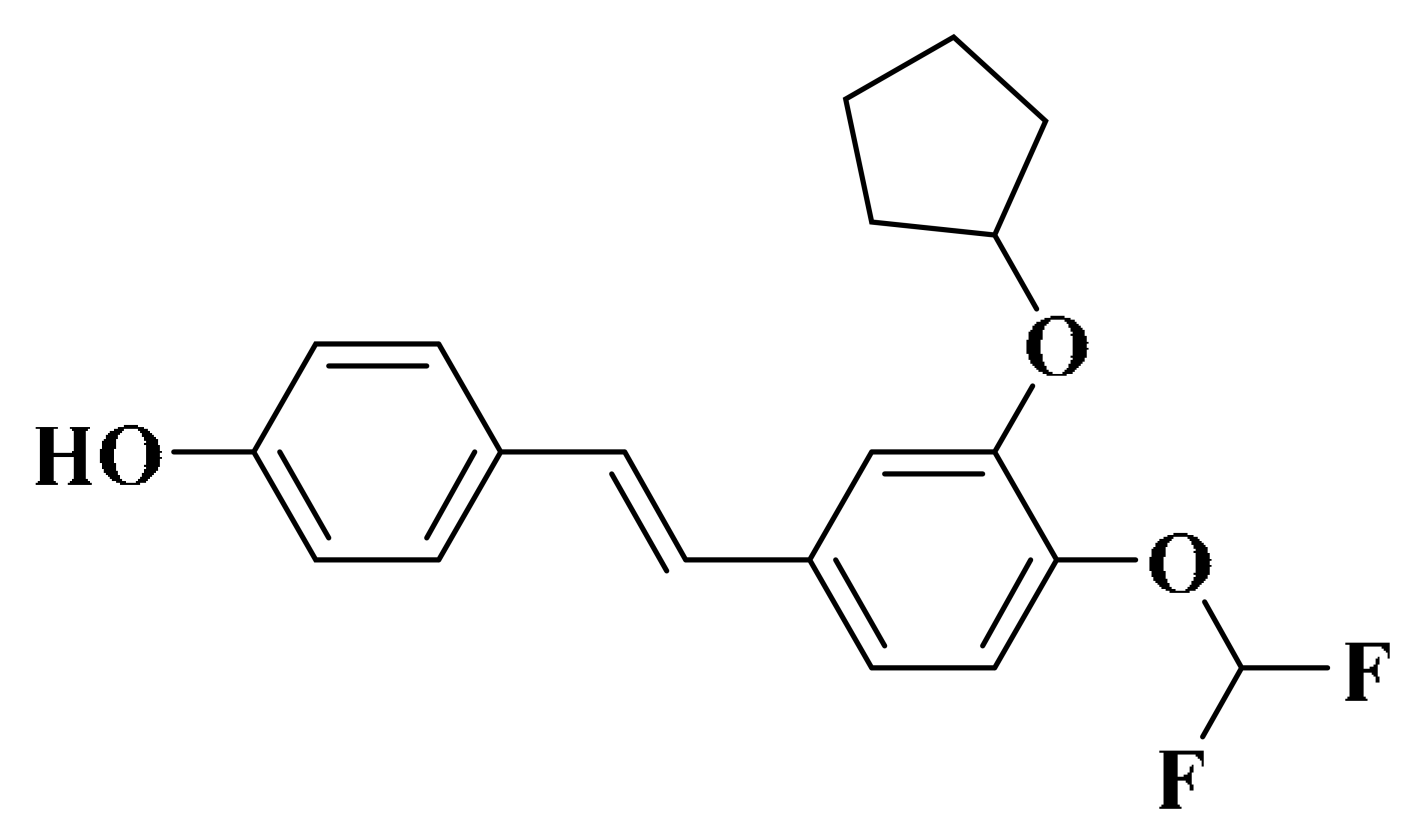	70.2 ± 2.5	35.2 ± 0.6
**3h** *	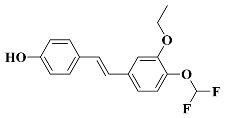	82.1 ± 1.9	34.7 ± 0.1
**3i** *	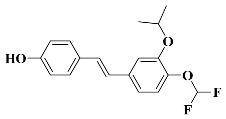	71.4 ± 1.7	35.8 ± 0.2
**3j** *	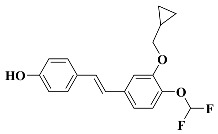	60.6 ± 0.8	32.4 ± 0.4
**4a** *	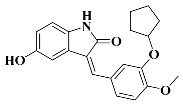	48.5 ± 2.4	69.8 ± 0.1
**4b** *	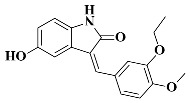	64.5 ± 1.3	70.7 ± 0.5
**4c** *	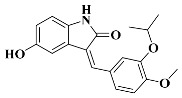	44.2 ± 2.5	76.7 ± 0.8
**4d** *	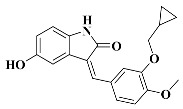	47.2 ± 1.7	68.4 ± 0.3
**4e** *	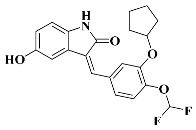	35.5 ± 2.4	73.8 ± 0.8
**4f** *	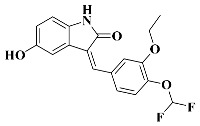	78.4 ± 1.8	68.8 ± 0.1
**4g** *	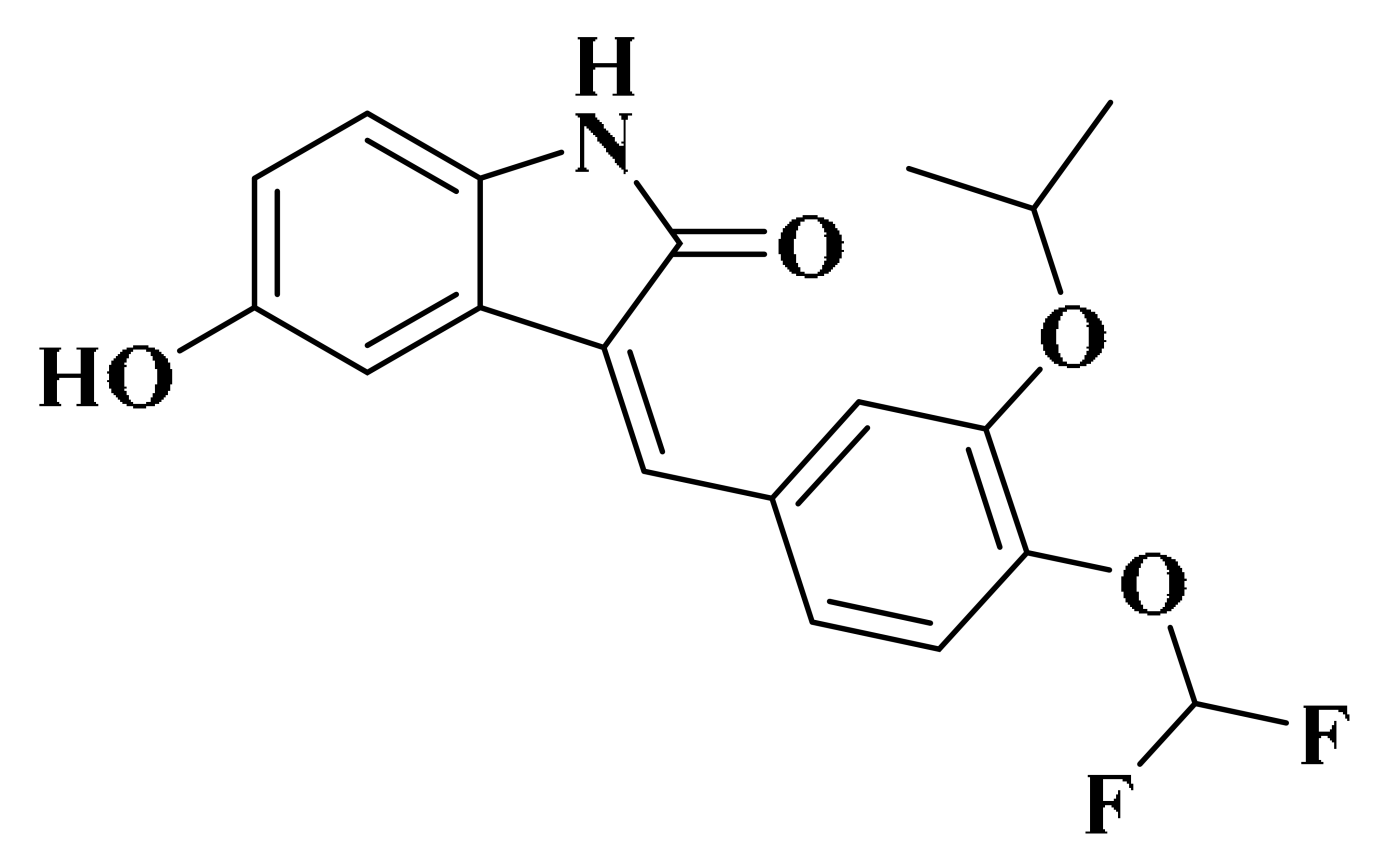	41.5 ± 2.6	78.1 ± 0.4
**4h** *	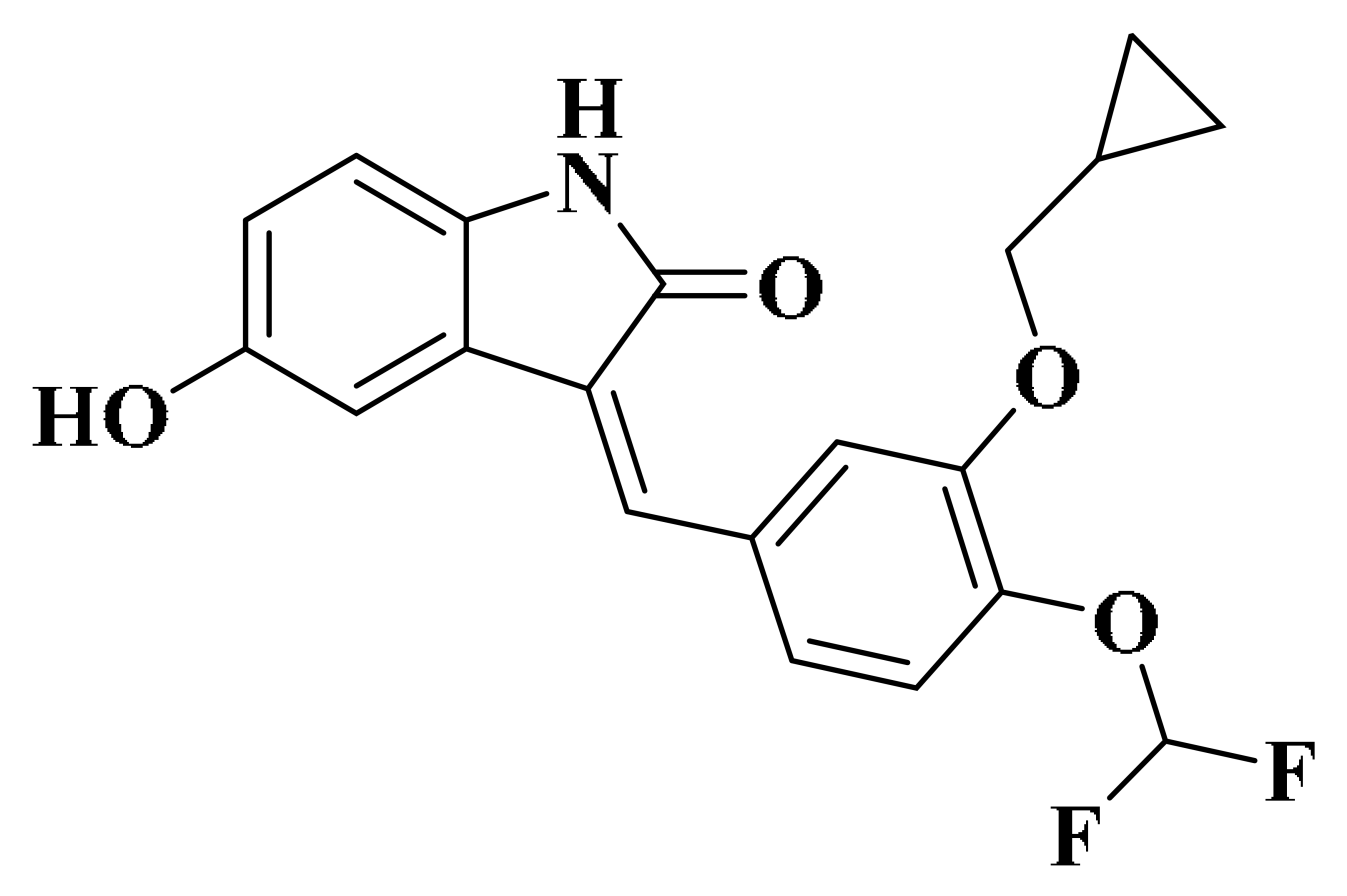	47.0 ± 1.5	69.8 ± 0.2
Rolipram	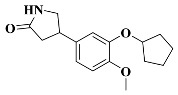	84.8 ± 1.6	ND
Edaravone	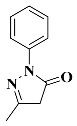	ND	65.3 ± 0.7

The value is expressed as mean ± SD (*n* = 2). *: new molecular entity; ND: not determined.

**Table 3:** IC_50_ values of the candidate compounds against PDE4 and DPPH.

NO	Structure	IC_50_ (nM)	IC_50_ (µM)
		PDE4	DPPH
Resveratrol	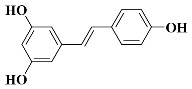	6006 ± 844	75.9 ± 0.4
**3d** *	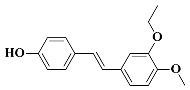	218 ± 22	82.2 ± 0.8
**3h** *	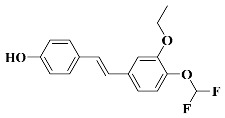	160 ± 35	78.7 ± 0.2
**4b** *	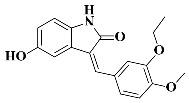	631 ± 27	38.4 ± 0.3
**4f** *	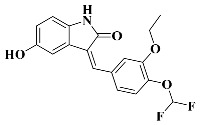	469 ± 21	35.7 ± 0.5
Rolipram	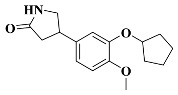	170 ± 22	ND
Edaravone	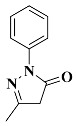	ND	37.9 ± 0.1

The value is expressed as mean ± SD (*n* = 2). *: new molecular entity; ND: not determined.

**Table 4:** Anti-lipid peroxidation effect of compound **WYZ69** in Fe^2+^-induced mouse lung homogenates.

Dose (µM)	Inhibition Rate (%) of MDA Levels	
	WYZ69	Edaravone
250	52.6 ± 0.5	10.5 ± 0.9
500	82.5 ± 0.4	17.5 ± 0.6
1000	98.2 ± 0.6	28.1 ± 0.5

The value is expressed as mean ± SD (*n* = 3).

**Table 5:** Pharmacokinetic properties of compound **WYZ69** in mice.

Compound	T_max_ (h)	C_max_ (ng/mL)	AUC_0-t_ (ng·h/mL)	AUC_0-∞_ (ng·h/mL)	MRT_0-∞_ (h)	T_1/2_ (h)	Vz (mL/kg)	CL (mL/(kg·h))
**WYZ69** (1mg/kg, i.v)	0.08 ± 0.00	1306.40 ± 228.56	1134.8 8 ± 104.42	1150.65 ± 105.09	2.01 ± 0.18	1.63 ± 0.04	2064.22 ± 199.82	874.35 ± 79.26

The value is expressed as mean ± SD (*n* = 3).

**Table 6:** Anti-TNF-α production effect of compound **WYZ69** in LPS-induced mice.

Dose (mg/kg, i.p.)	Inhibition Rate (%) of TNF-α Levels	
	WYZ69	Rolipram
10	29.6	72.1
30	59.2	ND

The value is expressed as mean (*n* = 8); ND: not determined.

## Data Availability

The original contributions presented in this study are included in the article/Supplementary Material, see as Figures S1–S66. Further inquiries can be directed to the corresponding author.

## Supplementary Materials

The following supporting information can be downloaded at: https://www.mdpi.com/article/10.3390/antiox15070899/s1, 1. Yield, ^1^HNMR and LRMS of Benzaldehyde Intermediates **1e-1h** and **1r-1u**. 2. Yield, ^1^HNMR and LRMS of Styrene Intermediates **2a-2h**. 3. Yield, ^1^HNMR, ^13^CNMR and HRMS of Target Compounds **3a-3j**. 4. Yield, ^1^HNMR, ^13^CNMR and HRMS of Target Compounds **4a-4h**. 5. ^1^HNMR, ^13^CNMR and HRMS Spectra of Synthetic Compounds (Figures S1–S66).
