# Supplementary material for: Novel Resveratrol Derivatives as Dual PDE4 Inhibitors and Free Radical Scavengers: Rational Design, Synthesis, and Biological Evaluation
Source: Antioxidants (Basel). 2026 Jul 20;15(7):899. doi: 10.3390/antiox15070899 (PMC13405238; doi:10.3390/antiox15070899)
Supplement: Supplementary file 1 [file antioxidants-15-00899-s001.zip › antioxidants-4343145-supplementary.pdf]

Supporting Information

Novel Resveratrol Derivatives as Dual PDE4 Inhibitors and Free Radical Scavengers: Rational Design, Synthesis, and Biological Evaluation

Youzhi Wang [1,2], Huizhen Shen [1], Ying liang [1], Guoqing Yang [1], Botao Zhang [1], Yunbao Zhi [1], Jinxin Wang [1,*]

[1] Jiangsu Key Laboratory of Drug Design and Optimization, Department of Medicinal Chemistry, School of Pharmacy, China Pharmaceutical University, Nanjing 210009, China

[2] Institute of Traditional Chinese Medicine, Wuxi Affiliated Hospital of Nanjing University of Chinese Medicine, Wuxi 214071, China

* Correspondence: jinxinwang@163.com

Table of Contents:

1. Yield,^{1}HNMR and LRMS of Benzaldehyde Intermediates **1e-1h** and **1r-1uError! Bookmark not define**
2. Yield,^{1}HNMR and LRMS of Styrene Intermediates **2a-2h** .... **Error! Bookmark not defined.**
3. Yield,^{1}HNMR, ^{13}CNMR and HRMS of Target Compounds **3a-3j** 2
4. Yield,^{1}HNMR, ^{13}CNMR and HRMS of Target Compounds **4a-4h** 3
5. ^{1}HNMR, ^{13}CNMR and HRMS Spectra of Synthetic Compounds 5

1.Yield,^{1}HNMR and LRMS of Benzaldehyde Intermediates 1e-1h and 1r-1u

3-(cyclopropylmethoxy)-4-(difluoromethoxy)benzaldehyde ***(1r).*** Yield: 784 mg (81%) white solid. ^{1}H NMR (300 MHz, DMSO) δ 9.94 (s, 1H), 7.63 – 7.52 (m, 2.25H, ArH and CF_2H), 7.40 (d, J = 8.2 Hz, 1H), 7.29 (s, 0.5H, CF_2H), 7.05 (s, 0.25H, CF_2H), 3.98 (d, J = 7.0 Hz, 2H), 1.31 – 1.22 (m, 1H), 0.65 – 0.51 (m, 2H), 0.44 – 0.28 (m, 2H). LRMS (ESI) *m/z* for $C_{12}H_{12}F_2O_3$ $[M+H]^+$: found 243.0.

4-(difluoromethoxy)-3-ethoxybenzaldehyde ***(1s).*** Yield: 648 mg (75%) white solid. ^{1}H NMR (300 MHz, DMSO) δ 9.96 (s, 1H), 7.61 – 7.52 (m, 2.25H, ArH and CF_2H), 7.40 (d, J = 8.0 Hz, 1H), 7.28 (s, 0.5H, CF_2H), 7.04 (s, 0.25H, CF_2H), 4.19 (q, J = 7.0 Hz, 2H), 1.38 (t, J = 7.0 Hz, 3H). LRMS (ESI) *m/z* for $C_{10}H_{10}F_2O_3$ $[M+H]^+$: found 217.0.

4-(difluoromethoxy)-3-isopropoxybenzaldehyde ***(1t).*** Yield: 727 mg (79%) white solid. ^{1}H NMR (300 MHz, DMSO) δ 9.95 (s, 1H), 7.63 (d, J = 1.8 Hz, 1H), 7.56 (dd, J = 8.2, 1.8 Hz, 1H), 7.49 (s, 0.25H, CF_2H), 7.39 (d, J = 8.2 Hz, 1H), 7.24 (s, 0.5H, CF_2H), 6.99 (s, 0.25H, CF_2H), 4.76 (p, J = 6.0 Hz, 1H), 1.33 (s, 3H), 1.31 (s, 3H). LRMS (ESI) *m/z* for $C_{11}H_{12}F_2O_3$ $[M+H]^+$: found 231.0.

3-(cyclopentyloxy)-4-(difluoromethoxy)benzaldehyde ***(1u).*** Yield: 778 mg (76%) white solid. ^{1}H NMR (300 MHz, DMSO) δ 9.96 (s, 1H), 7.62 – 7.52 (m, 2H), 7.45 (s, 0.25H, CF_2H), 7.39 (d, J = 8.1 Hz, 1H), 7.21 (s, 0.50H, CF_2H), 6.96 (s, 0.25H, CF_2H), 4.99 (tt, J = 5.7, 2.3 Hz, 1H), 2.02 – 1.89 (m, 2H), 1.84 – 1.67 (m, 4H), 1.67 – 1.54 (m, 2H). LRMS (ESI) *m/z* for $C_{13}H_{14}F2O_3$ $[M+H]^+$: found 257.0.

2. Yield,^{1}HNMR and LRMS of Styrene Intermediates 2a-2h

2-(cyclopropylmethoxy)-1-methoxy-4-vinylbenzene ***(2a).*** Yield: 322 mg (79%) white solid. ^{1}H NMR (300 MHz, DMSO) δ 7.05 (d, J = 1.9 Hz, 1H), 6.96 (dd, J = 8.3, 1.9 Hz, 1H), 6.91 (d, J = 8.3 Hz, 1H), 6.62 (dd, J = 17.7, 10.9 Hz, 1H), 5.68 (dd, J = 17.6, 1.1 Hz, 1H), 5.11 (dd, J = 10.9, 1.1 Hz, 1H), 3.81 (d, J = 7.0 Hz, 2H), 3.76 (s, 3H), 1.23 (qd, J = 4.8, 4.0, 2.2 Hz, 1H), 0.66 – 0.46 (m, 2H), 0.36 – 0.23 (m, 2H). LRMS (ESI) *m/z* for $C_{13}H_{16}O_2$ $[M+H]^+$: found 205.1.

2-ethoxy-1-methoxy-4-vinylbenzene ***(2b).*** Yield: 252 mg (71%) white solid. ^{1}H NMR (300 MHz, DMSO) δ 7.07 (d, J = 1.9 Hz, 1H), 6.96 (dd, J = 8.3, 1.9 Hz, 1H), 6.91 (d, J = 8.3 Hz, 1H), 6.64 (dd, J = 17.6, 10.9 Hz, 1H), 5.69 (dd, J = 17.6, 1.1 Hz, 1H), 5.11 (dd, J = 10.9, 1.1 Hz, 1H), 4.03 (q, J = 7.0 Hz, 2H), 3.75 (s, 3H), 1.33 (t, J = 7.0 Hz, 3H). LRMS (ESI) *m/z* for $C_{11}H_{14}O_2$ $[M+H]^+$: found 179.1.

2-isopropoxy-1-methoxy-4-vinylbenzene ***(2c).*** Yield: 284 mg (74%) white solid. ^{1}H NMR (300 MHz, DMSO) δ 7.08 (d, J = 1.9 Hz, 1H), 6.98 (dd, J = 8.3, 2.0 Hz, 1H), 6.92 (d, J = 8.3 Hz, 1H), 6.63 (dd, J = 17.6, 10.9 Hz, 1H), 5.68 (dd, J = 17.6, 1.1 Hz, 1H), 5.11 (dd, J = 10.9, 1.1 Hz, 1H), 4.60 (h, J = 6.0 Hz, 1H), 3.75 (s, 3H), 1.26 (s, 3H), 1.24 (s, 3H). LRMS (ESI) *m/z* for $C_{12}H_{16}O_2$ $[M+H]^+$: found 193.1.

2-(cyclopentyloxy)-1-methoxy-4-vinylbenzene ***(2d).*** Yield: 336 mg (77%) white solid. ^{1}H NMR (300 MHz, DMSO) δ 7.04 (d, J = 1.9 Hz, 1H), 6.96 (dd, J = 8.3, 1.9 Hz, 1H), 6.90 (d, J = 8.3 Hz, 1H), 6.64 (dd, J = 17.6, 10.9 Hz, 1H), 5.68 (dd, J = 17.6, 1.1 Hz, 1H), 5.11 (dd, J = 10.8, 1.1 Hz, 1H), 4.91 – 4.72 (m, 1H), 3.74 (s, 3H), 1.96 – 1.81 (m, 2H), 1.78 – 1.64 (m, 4H), 1.64 – 1.49 (m, 2H). LRMS (ESI) *m/z* for $C_{14}H_{18}O_2$ $[M+H]^+$: found 219.1.

2-(cyclopropylmethoxy)-1-(difluoromethoxy)-4-vinylbenzene ***(2e).*** Yield: 350 mg (73%) white solid. ^{1}H NMR (300 MHz, DMSO) δ 7.32 (s, 0.25H, CF_2H), 7.24 (s, 1H), 7.13 (d, J = 8.2 Hz, 1H), 7.07 (s, 0.52H, CF_2H), 7.04 (dd, J = 8.3, 1.9 Hz, 1H), 6.82 (s, 0.24H, CF_2H), 6.69 (dd, J = 17.6, 11.0 Hz, 1H), 5.84 (dd, J = 17.6, 1.0 Hz, 1H), 5.27 (dd, J = 10.9, 0.9 Hz, 1H), 3.93 (d, J = 6.9 Hz, 2H), 1.28 – 1.21 (m, 1H), 0.62 – 0.53 (m, 2H), 0.40 – 0.30 (m, 2H). LRMS (ESI) *m/z* for $C_{13}H_{14}F_2O_2$ $[M+H]^+$: found 241.1.

1-(difluoromethoxy)-2-ethoxy-4-vinylbenzene ***(2f).*** Yield: 308 mg (72%) white solid. ^{1}H NMR (300 MHz, DMSO) δ 7.31 (s, 0.25H, CF_2H), 7.25 (d, J = 1.9 Hz, 1H), 7.13 (d, J = 8.2 Hz, 1H), 7.08 – 6.97 (m, 1.50H, ArH and CF_2H), 6.81 (s, 0.25H, CF_2H), 6.71 (dd, J = 17.6, 10.9 Hz, 1H), 5.85 (dd, J = 17.7, 0.9 Hz, 1H), 5.27 (dd, J = 10.9, 1.0 Hz, 1H), 4.13 (q, J = 7.0 Hz,

2H), 1.35 (t, *J* = 7.0 Hz, 3H). LRMS (ESI) *m/z* for $C_{11}H_{12}F_2O_2$ [M+H]$^+$: found 215.0.

1-(difluoromethoxy)-2-isopropoxy-4-vinylbenzene ***(2g).*** Yield: 228 mg (74%) white solid. ^{1}H NMR (300 MHz, DMSO) δ 7.27 – 7.25 (m, 1.25H, ArH and CF_2H), 7.13 (d, *J* = 8.3 Hz, 1H), 7.05 (dd, *J* = 8.2, 2.0 Hz, 1H), 7.02 (s, 0.50H, CF_2H), 6.77 (s, 0.25H, CF_2H), 6.71 (dd, *J* = 17.7, 10.9 Hz, 1H), 5.84 (dd, *J* = 17.6, 0.9 Hz, 1H), 5.27 (dd, *J* = 10.9, 0.9 Hz, 1H), 4.71 (hept, *J* = 6.2 Hz, 1H), 1.30 (s, 3H), 1.28 (s, 3H). LRMS (ESI) *m/z* for $C_{12}H_{14}F_2O_2$ [M+H]$^+$: found 229.1.

2-(cyclopentyloxy)-1-(difluoromethoxy)-4-vinylbenzene ***(2h).*** Yield: 396 mg (78%) white solid.^{1}H NMR (300 MHz, DMSO) δ 7.25 – 7.20 (m, 1.25H, ArH and CF_2H), 7.12 (d, *J* = 8.2 Hz, 1H), 7.04 (dd, *J* = 8.3, 1.9 Hz, 1H), 6.98 (s, 0.50H, CF_2H), 6.77 – 6.65 (m, 1.25H, CH and CF_2H), 5.84 (dd, *J* = 17.6, 0.9 Hz, 1H), 5.27 (dd, *J* = 10.9, 0.9 Hz, 1H), 4.98 – 4.91 (m, 1H), 1.97 – 1.85 (m, 2H), 1.79 – 1.67 (m, 4H), 1.64 – 1.52 (m, 2H). LRMS (ESI) *m/z* for $C_{14}H_{16}F_2O_2$ [M+H]$^+$: found 255.1.

3. Yield,^{1}HNMR, ^{13}CNMR and HRMS of Target Compounds 3a-3j

(E)-5-(3-(cyclopentyloxy)-4-methoxystyryl)benzene-1,3-diol ***(3a).*** Yield: 130 mg (40%) grey solid. ^{1}H NMR (300 MHz, DMSO) δ 9.24 (s, 2H), 7.17 (d, *J* = 2.0 Hz, 1H), 7.07 (dd, *J* = 8.4, 1.9 Hz, 1H), 7.02 – 6.84 (m, 3H), 6.42 (d, *J* = 2.1 Hz, 2H), 6.14 (t, *J* = 2.1 Hz, 1H), 4.88 (tt, *J* = 5.9, 2.5 Hz, 1H), 3.75 (s, 3H), 1.99 – 1.85 (m, 2H), 1.73 (q, *J* = 5.3 Hz, 4H), 1.64 – 1.54 (m, 2H). ^{13}C NMR (101 MHz, DMSO) δ 158.97, 150.00, 147.56, 139.61, 130.40, 128.40, 127.19, 120.15, 113.00, 112.68, 104.89, 102.38, 79.89, 56.00, 32.74, 24.11. HRMS (ESI) *m/z* for $C_{20}H_{22}O_4$ [M-H]-: calcd, 325.1445; found, 325.1448.

(E)-3-(3-(cyclopentyloxy)-4-methoxystyryl)phenol ***(3b).*** Yield: 102 mg (33%), white solid. ^{1}H NMR (300 MHz, DMSO) δ 9.40 (s, 1H), 7.19 (d, *J* = 2.0 Hz, 1H), 7.16 – 7.07 (m, 2H), 7.06 – 6.91 (m, 5H), 6.66 (ddd, *J* = 8.0, 2.4, 1.0 Hz, 1H), 4.87 (td, *J* = 5.9, 2.8 Hz, 1H), 3.76 (s, 3H), 1.97 – 1.84 (m, 2H), 1.80 – 1.68 (m, 4H), 1.64 – 1.52 (m, 2H). ^{13}C NMR (101 MHz, DMSO) δ 158.08, 150.06, 147.58, 139.22, 130.39, 130.02, 128.72, 126.85, 120.19, 117.71, 114.85, 113.24, 113.00, 112.68, 79.91, 55.99, 32.75, 24.12. HRMS (ESI) *m/z* for $C_{20}H_{22}O_3$ [M-H]$^-$: calcd, 309.1496; found, 309.1493.

(E)-4-(3-(cyclopentyloxy)-4-methoxystyryl)phenol ***(3c).*** Yield: 130 mg (42%), white solid. ^{1}H NMR (300 MHz, DMSO) δ 9.53 (s, 1H), 7.42 – 7.35 (m, 2H), 7.13 (d, *J* = 2.0 Hz, 1H), 7.03 (dd, *J* = 8.3, 2.0 Hz, 1H), 7.01 – 6.86 (m, 3H), 6.76 (d, *J* = 8.6 Hz, 2H), 4.87 (td, *J* = 5.9, 3.0 Hz, 1H), 3.75 (s, 3H), 1.97 – 1.84 (m, 2H), 1.81 – 1.67 (m, 4H), 1.64 – 1.51 (m, 2H). ^{13}C NMR (101 MHz, DMSO) δ 157.38, 149.59, 147.56, 130.95, 128.91, 127.97, 126.75, 125.67, 119.60, 115.97, 112.77, 112.69, 79.89, 56.01, 32.75, 24.10. HRMS (ESI) *m/z* for $C_{20}H_{22}O_3$ [M+H]$^+$: calcd, 311.1641; found, 311.1633.

(E)-4-(3-ethoxy-4-methoxystyryl)phenol ***(3d).*** Yield: 105 mg (39%), white solid. ^{1}H NMR (300 MHz, DMSO) δ 9.53 (d, *J* = 0.9 Hz, 1H), 7.38 (d, *J* = 8.4 Hz, 2H), 7.16 (d, *J* = 1.9 Hz, 1H), 7.03 (d, *J* = 9.1 Hz, 1H), 6.97 – 6.88 (m, 3H), 6.76 (d, *J* = 8.6 Hz, 2H), 4.06 (q, *J* = 7.0 Hz, 2H), 3.76 (s, 3H), 1.35 (t, *J* = 7.0 Hz, 3H). ^{13}C NMR (101 MHz, DMSO) δ 157.41, 148.86, 148.60, 130.97, 128.92, 127.95, 126.77, 125.64, 119.72, 116.00, 112.36, 110.51, 64.11, 55.89, 15.27. HRMS (ESI) *m/z* for $C_{17}H_{18}O_3$ [M-H]$^-$: calcd, 269.1183; found, 269.1185.

(E)-4-(3-isopropoxy-4-methoxystyryl)phenol ***(3e).*** Yield: 116 mg (41%), white solid. ^{1}H NMR (300 MHz, DMSO) δ 9.54 (s, 1H), 7.38 (d, *J* = 8.6 Hz, 2H), 7.16 (d, *J* = 2.0 Hz, 1H), 7.05 (dd, *J* = 8.3, 1.9 Hz, 1H), 7.03 – 6.86 (m, 3H), 6.76 (d, *J* = 8.5 Hz, 2H), 4.61 (p, *J* = 6.0 Hz, 1H), 3.75 (s, 3H), 1.28 (s, 3H), 1.26 (s, 3H). ^{13}C NMR (101 MHz, DMSO) δ 157.40, 149.95, 147.25, 130.99, 128.92, 127.97, 126.79, 125.58, 119.99, 115.99, 113.58, 112.85, 70.76, 55.95, 22.44. HRMS (ESI) *m/z* for $C_{18}H_{20}O_3$ [M+H]$^+$: calcd, 285.1485; found, 285.1479.

(*E)-4-(3-(cyclopropylmethoxy)-4-methoxystyryl)phenol* ***(3f).*** Yield: 133 mg (45%), white solid. ^{1}H NMR (300 MHz, DMSO) δ 9.54 (s, 1H), 7.38 (d, *J* = 8.5 Hz, 2H), 7.14 (d, *J* = 2.0 Hz, 1H), 7.07 – 6.87 (m, 4H), 6.75 (d, *J* = 8.4 Hz, 2H), 3.85 (d, *J* = 7.0 Hz, 2H), 3.77 (s, 3H), 1.27 – 1.21 (m, 1H), 0.68 – 0.46 (m, 2H), 0.41 – 0.24 (m, 2H). ^{13}C NMR (101 MHz, DMSO) δ 157.39, 148.88, 148.71, 130.96, 128.92, 127.94, 126.74, 125.64, 119.78, 115.99, 112.36, 110.58, 73.24, 55.90, 10.76, 3.67. HRMS (ESI) *m/z* for $C_{19}H_{20}O_3$ [M-H]$^-$: calcd, 295.1339; found, 295.1336.

(E)-4-(3-(cyclopentyloxy)-4-(difluoromethoxy)styryl)phenol ***(3g).*** Yield: 128 mg (37%), grey solid. ^{1}H NMR (300 MHz, DMSO) δ 9.63 (s, 1H), 7.43 (d, *J* = 8.6 Hz, 2H), 7.29 (d, *J* = 1.4 Hz, 1H), 7.22 (s, 0.25H, CF_2H), 7.19 – 7.07 (m, 3H), 7.05 – 6.94 (m, 1.50H, ArH and CF_2H), 6.78 (d, *J* = 8.5 Hz, 2H), 6.72 (s, 0.25H, CF_2H), 5.05 – 4.81 (m, 1H), 2.02 – 1.87 (m, 2H), 1.82 – 1.67 (m, 4H), 1.68 – 1.56 (m, 2H). ^{13}C NMR (101 MHz, DMSO) δ 157.86, 149.59, 139.83, 139.80, 139.77, 136.79, 129.33, 128.43, 128.38, 124.82, 122.24, 119.94, 118.83, 117.38, 116.03, 114.82, 113.19, 80.30, 32.69, 23.97. HRMS (ESI) *m/z* for $C_{20}H_{20}F_2O_3$ $[M-H]^-$: calcd, 345.1307.14533; found, 345.1306.

(E)-4-(4-(difluoromethoxy)-3-ethoxystyryl)phenol ***(3h).*** Yield: 113 mg (37%) grey solid. ^{1}H NMR (300 MHz, DMSO) δ 9.64 (s, 1H), 7.42 (d, *J* = 8.6 Hz, 2H), 7.32 (d, *J* = 1.6 Hz, 1H), 7.30 (s, 0.25H, CF_2H), 7.22 – 7.09 (m, 3H), 7.06 – 6.96 (m, 1.50H, CH and CF_2H), 6.82 – 6.75 (m, 2.25H, ArH and CF_2H), 4.15 (q, *J* = 7.0 Hz, 2H), 1.37 (t, *J* = 7.0 Hz, 3H). ^{13}C NMR (101 MHz, DMSO) δ 157.87, 150.49, 139.13, 139.10, 139.07, 136.81, 129.36, 128.43, 128.37, 124.76, 121.85, 119.84, 119.08, 117.29, 116.06, 114.73, 111.67, 64.54, 15.01. HRMS (ESI) *m/z* for $C_{17}H_{16}F_2O_3$ $[M-H]^-$: calcd, 305.0994; found, 305.0989.

(E)-4-(4-(difluoromethoxy)-3-isopropoxystyryl)phenol ***(3i).*** Yield: 112 mg (35%) grey solid. ^{1}H NMR (300 MHz, DMSO) δ 9.63 (s, 1H), 7.42 (d, *J* = 8.6 Hz, 2H), 7.33 (s, 1H), 7.26 (s, 0.25H, CF_2H), 7.20 – 7.09 (m, 3H), 7.04 – 6.95 (m, 1.50H, CH and CF_2H), 6.82 – 6.74 (m, 2.25H, ArH and CF_2H), 4.72 (hept, *J* = 6.0 Hz, 1H), 1.32 (s, 3H), 1.30 (s, 3H). ^{13}C NMR (101 MHz, DMSO) δ 157.86, 149.39, 140.07, 140.03, 140.00, 136.81, 129.35, 128.44, 128.37, 124.77, 122.16, 119.85, 119.14, 117.29, 116.04, 114.73, 113.77, 71.30, 22.24. HRMS (ESI) *m/z* for $C_{18}H_{18}F_2O_3$ $[M-H]^-$: calcd, 319.1151; found, 319.1146.

(E)-4-(3-(cyclopropylmethoxy)-4-(difluoromethoxy)styryl)phenol ***(3j).*** Yield: 103 mg (31%) grey solid. ^{1}H NMR (300 MHz, DMSO) δ 9.63 (s, 1H), 7.42 (d, *J* = 8.6 Hz, 2H), 7.32 – 7.28 (m, 1.25H, ArH and CF_2H), 7.21 – 7.09 (m, 3H), 7.06 – 6.95 (m, 1.50H, CH and CF_2H), 6.82 – 6.75 (m, 2.25H, ArH and CF_2H), 3.95 (d, *J* = 7.0 Hz, 2H), 1.29 – 1.22 (m, 1H), 0.64 – 0.54 (m, 2H), 0.42 – 0.31 (m, 2H). ^{13}C NMR (101 MHz, DMSO) δ 157.85, 150.59, 139.20, 139.17, 139.14, 136.78, 129.34, 128.43, 128.35, 124.75, 121.83, 119.84, 119.21, 117.28, 116.05, 114.72, 111.90, 73.41, 10.51, 3.48. HRMS (ESI) *m/z* for $C_{19}H_{18}F_2O_3$ $[M-H]^-$: calcd, 331.1151; found, 331.1149.

4. Yield,^{1}HNMR, ^{13}CNMR and HRMS of Target Compounds 4a-4h

(Z)-3-(3-(cyclopentyloxy)-4-methoxybenzylidene)-5-hydroxyindolin-2-one ***(4a).*** Yield: 42 mg (12%) red solid. ^{1}H NMR (400 MHz, DMSO) δ 10.23 (s, 1H), 8.98 (s, 1H), 8.72 (d, J = 2.0 Hz, 1H), 7.75 (dd, *J* = 8.5, 2.0 Hz, 1H), 7.59 (s, 1H), 7.09 (s, 1H), 7.04 (d, *J* = 8.5 Hz, 1H), 6.62 (s, 2H), 4.87 – 4.80 (m, 1H), 3.83 (s, 3H), 2.06 – 1.98 (m, 2H), 1.80 – 1.70 (m, 4H), 1.62 – 1.56 (m, 2H). ^{13}C NMR (101 MHz, DMSO) δ 168.08, 152.64, 152.16, 146.76, 137.65, 133.22, 127.89, 127.67, 126.93, 125.02, 117.74, 115.32, 111.70, 110.08, 107.12, 79.92, 55.98, 32.83, 24.18. HRMS (ESI) *m/z* for $C_{21}H_{21}NO_4$ $[M+H]^+$: calcd, 352.1543; found, 352.1533.

(Z)-3-(3-ethoxy-4-methoxybenzylidene)-5-hydroxyindolin-2-one ***(4b).*** Yield: 43 mg (14%) red solid. ^{1}H NMR (400 MHz, DMSO) δ 10.23 (s, 1H), 8.98 (s, 1H), 8.69 (s, 1H), 7.81 (d, *J* = 8.5 Hz, 1H), 7.59 (s, 1H), 7.15 – 6.98 (m, 2H), 6.63 (s, 2H), 4.10 (q, *J* = 7.0 Hz, 2H), 3.84 (s, 3H), 1.38 (t, *J* = 7.1 Hz, 3H). ^{13}C NMR (101 MHz, DMSO) δ 168.05, 152.66, 151.58, 147.58, 137.55, 133.26, 127.83, 127.70, 126.91, 125.12, 116.36, 115.38, 111.55, 110.11, 107.11, 64.06, 55.95, 15.17. HRMS (ESI) *m/z* for $C_{18}H_{17}NO_4$ $[M+H]^+$: calcd, 312.1230; found, 312.1218.

(Z)-5-hydroxy-3-(3-isopropoxy-4-methoxybenzylidene)indolin-2-one ***(4c).*** Yield: 42 mg (13%) red solid. ^{1}H NMR (300 MHz, DMSO) δ 10.26 (s, 1H), 9.02 (s, 1H), 8.73 (d, *J* = 2.1 Hz, 1H), 7.78 (dd, *J* = 8.6, 2.0 Hz, 1H), 7.61 (s, 1H), 7.11 – 7.03 (m, 2H), 6.64 (s, 2H), 4.63 (p, *J* = 6.1 Hz, 1H), 3.84 (s, 3H), 1.34 (s, 3H), 1.32 (s, 3H). ^{13}C NMR (101 MHz, DMSO) δ 168.07, 152.65, 152.36, 146.42, 137.59, 133.24, 127.93, 127.66, 126.90, 125.07, 118.19, 115.36, 111.83, 110.10, 107.12, 70.63, 55.96, 22.40. HRMS (ESI) *m/z* for $C_{19}H_{19}NO_4$ $[M+H]^+$: calcd, 326.1386; found, 326.1368.

(Z)-3-(3-(cyclopropylmethoxy)-4-methoxybenzylidene)-5-hydroxyindolin-2-one ***(4d).*** Yield: 37 mg (11%) red solid. ^{1}H

NMR (400 MHz, DMSO) δ 10.25 (s, 1H), 9.00 (s, 1H), 8.65 (d, *J* = 2.0 Hz, 1H), 7.83 (dd, *J* = 8.6, 2.0 Hz, 1H), 7.59 (s, 1H), 7.12 – 7.03 (m, 2H), 6.62 (d, *J* = 1.4 Hz, 2H), 3.92 – 3.81 (m, 5H), 1.35 – 1.28 (m, 1H), 0.65 – 0.56 (m, 2H), 0.34 (dt, *J* = 5.9, 4.1 Hz, 2H). ^{13}C NMR (101 MHz, DMSO) δ 168.05, 152.66, 151.61, 147.70, 137.55, 133.25, 127.81, 127.68, 126.91, 125.10, 116.53, 115.37, 111.57, 110.10, 107.10, 73.19, 55.96, 10.66, 3.70. HRMS (ESI) *m/z* for $C_{20}H_{19}NO_4$ $[M+H]^+$: calcd, 338.1386; found, 338.1373.

(Z)-3-(3-(cyclopentyloxy)-4-(difluoromethoxy)benzylidene)-5-hydroxyindolin-2-one ***(4e).*** Yield: 46 mg (12%) red solid.^{1}H NMR (300 MHz, DMSO) δ 10.34 (s, 1H), 9.07 (s, 1H), 8.72 (d, *J* = 1.9 Hz, 1H), 7.74 (dd, *J* = 8.4, 1.9 Hz, 1H), 7.66 (s, 1H), 7.37 (s, 0.25H, CF_2H), 7.22 (d, *J* = 8.3 Hz, 1H), 7.12 (m, 1.50H, CH and CF_2H), 6.87 (s, 0.25H, CF_2H), 6.73 – 6.58 (m, 2H), 4.97 – 4.86 (m, 1H), 2.12 – 1.99 (m, 2H), 1.85 – 1.69 (m, 4H), 1.66 – 1.55 (m, 2H). ^{13}C NMR (101 MHz, DMSO) δ 167.81, 152.79, 148.61, 142.15, 136.20, 133.71, 132.76, 127.80, 126.35, 126.20, 120.84, 119.68, 118.63, 117.12, 116.19, 114.55, 110.34, 107.67, 80.57, 60.22, 32.75, 24.02. HRMS (ESI) *m/z* for $C_{21}H_{19}F_2NO_4[M+H]^+$: calcd, 388.1354; found, 388.1335.

(Z)-3-(4-(difluoromethoxy)-3-ethoxybenzylidene)-5-hydroxyindolin-2-one ***(4f).*** Yield: 52 mg (15%) red solid. ^{1}H NMR (300 MHz, DMSO) δ 10.33 (s, 1H), 9.08 (s, 1H), 8.68 (s, 1H), 7.80 (d, *J* = 7.8 Hz, 1H), 7.66 (s, 1H), 7.44 (s, 0.25H, CF_2H), 7.30 – 7.07 (m, 2.50H, ArH and CF_2H), 6.94 (s, 0.25H, CF_2H), 6.65 (s, 2H), 4.17 (q, *J* = 6.9 Hz, 2H), 1.40 (t, *J* = 7.0 Hz, 3H). ^{13}C NMR (101 MHz, DMSO) δ 167.76, 152.79, 149.27, 141.54, 136.13, 133.71, 132.74, 127.83, 126.33, 126.22, 120.39, 119.61, 117.51, 117.04, 116.20, 114.48, 110.36, 107.64, 64.56, 14.97. HRMS (ESI) *m/z* for $C_{18}H_{15}F_2NO_4[M-H]^-$: calcd, 346.0896; found, 346.0899.

(Z)-3-(4-(difluoromethoxy)-3-isopropoxybenzylidene)-5-hydroxyindolin-2-one ***(4g).*** Yield: 47 mg (13%) red solid. ^{1}H NMR (300 MHz, DMSO) δ 10.33 (s, 1H), 9.08 (s, 1H), 8.71 (d, *J* = 1.9 Hz, 1H), 7.74 (dd, *J* = 8.5, 1.9 Hz, 1H), 7.66 (s, 1H), 7.40 (s, 0.25H, CF_2H), 7.23 (d, *J* = 8.3 Hz, 1H), 7.16 – 7.11 (m, 1.50H, ArH and CF_2H), 6.90 (s, 0.25H, CF_2H), 6.66 (d, *J* = 2.1 Hz, 2H), 4.68 (p, *J* = 6.1 Hz, 1H), 1.37 (s, 3H), 1.35 (s, 3H). ^{13}C NMR (101 MHz, DMSO) δ 167.81, 152.79, 148.36, 142.26, 136.16, 133.71, 132.74, 127.80, 126.33, 126.27, 120.76, 119.60, 118.91, 117.04, 116.20, 114.47, 110.36, 107.66, 71.48, 22.20. HRMS (ESI) *m/z* for $C_{19}H_{17}F_2NO_4[M-H]^-$: calcd, 360.1052; found, 360.1050.

(Z)-3-(3-(cyclopropylmethoxy)-4-(difluoromethoxy)benzylidene)-5-hydroxyindolin-2-one ***(4h).*** Yield: 45 mg (12%) red solid. ^{1}H NMR (300 MHz, DMSO) δ 10.34 (s, 1H), 9.06 (s, 1H), 8.65 (d, *J* = 1.9 Hz, 1H), 7.82 (dd, *J* = 8.5, 1.9 Hz, 1H), 7.66 (s, 1H), 7.39 (s, 0.25H, CF_2H), 7.29 – 7.19 (m, 1.50H, ArH and CF_2H), 7.10 (d, *J* = 2.1 Hz, 1H), 7.02 (s, 0.25H, CF_2H), 6.73 – 6.53 (m, 2H), 3.96 (d, *J* = 7.0 Hz, 2H), 1.34 (m, 1H), 0.65 – 0.54 (m, 2H), 0.41 – 0.32 (m, 2H). ^{13}C NMR (101 MHz, DMSO) δ 167.76, 152.78, 149.36, 141.62, 136.13, 133.69, 132.70, 127.80, 126.32, 126.24, 120.37, 119.60, 117.83, 117.04, 116.19, 114.47, 110.35, 107.63, 73.44, 10.44, 3.56. HRMS (ESI) *m/z* for $C_{20}H_{17}F_2NO_4[M-H]^-$: calcd, 372.1052; found, 372.1056.

5. ^{1}HNMR, ^{13}CNMR, and HRMS Spectra of Synthetic Compounds

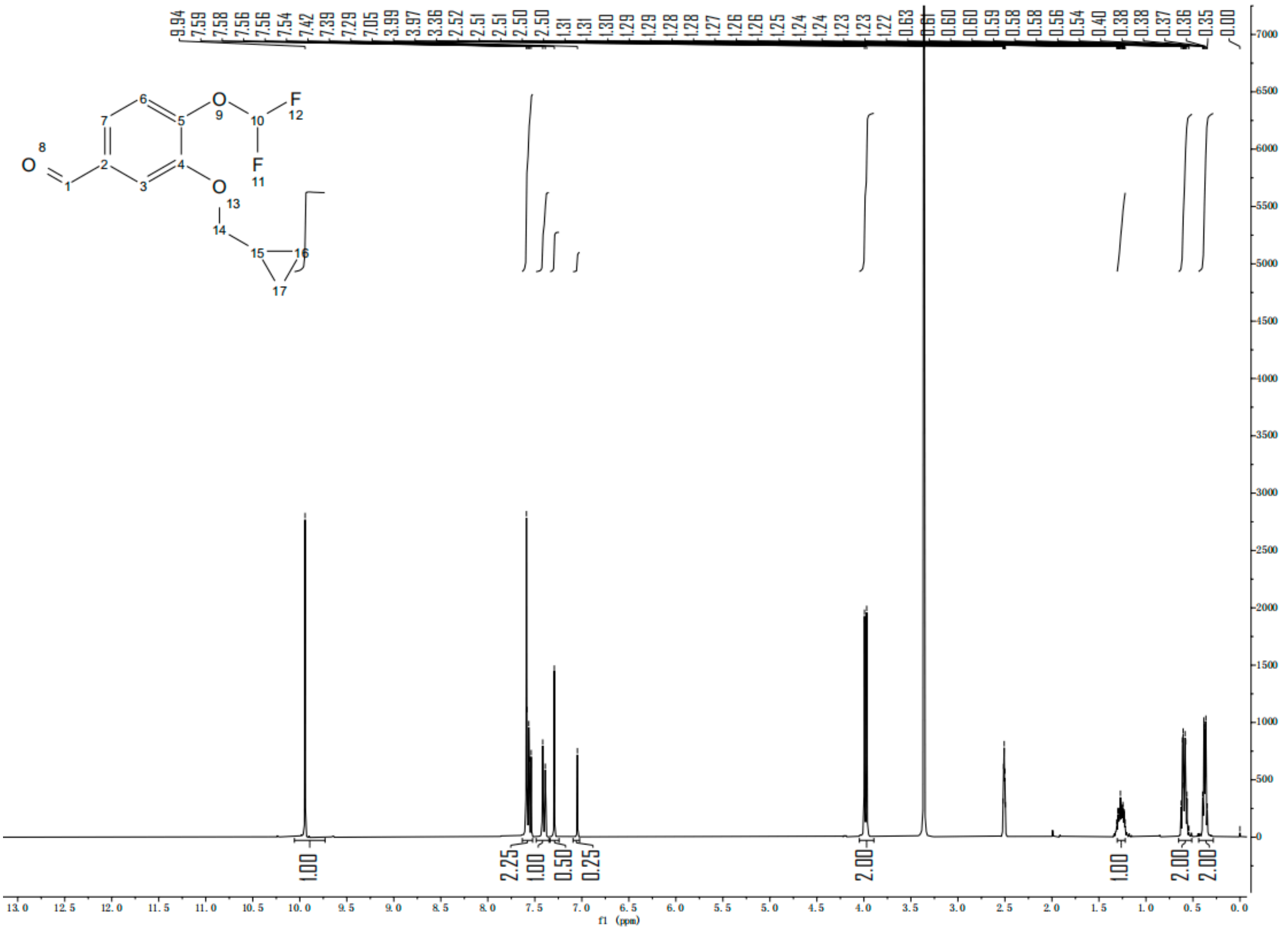

Figure S1. ^{1}H-NMR Spectrum of 1r (300 MHz, DMSO-d6)

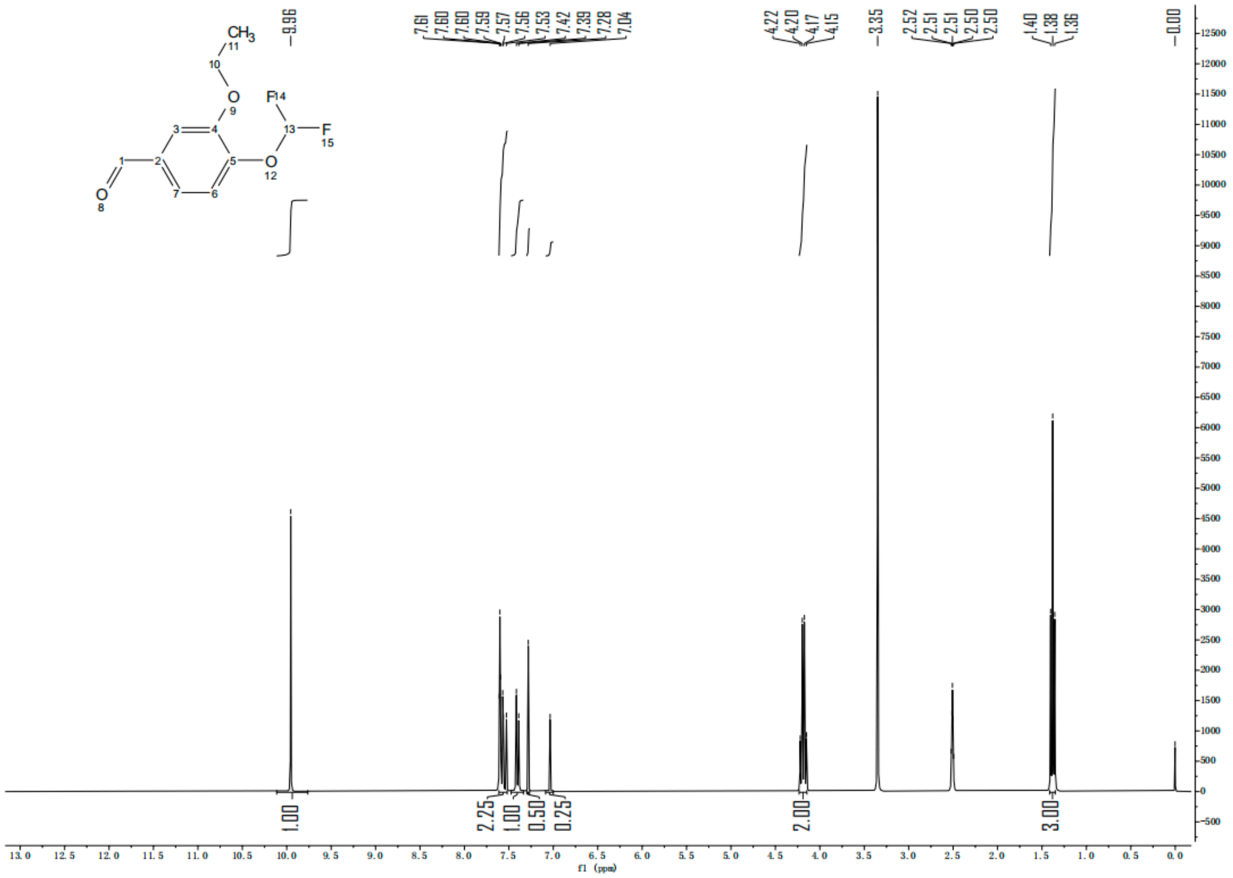

Figure S2. ^{1}H-NMR Spectrum of 1s (300 MHz, DMSO-d6)

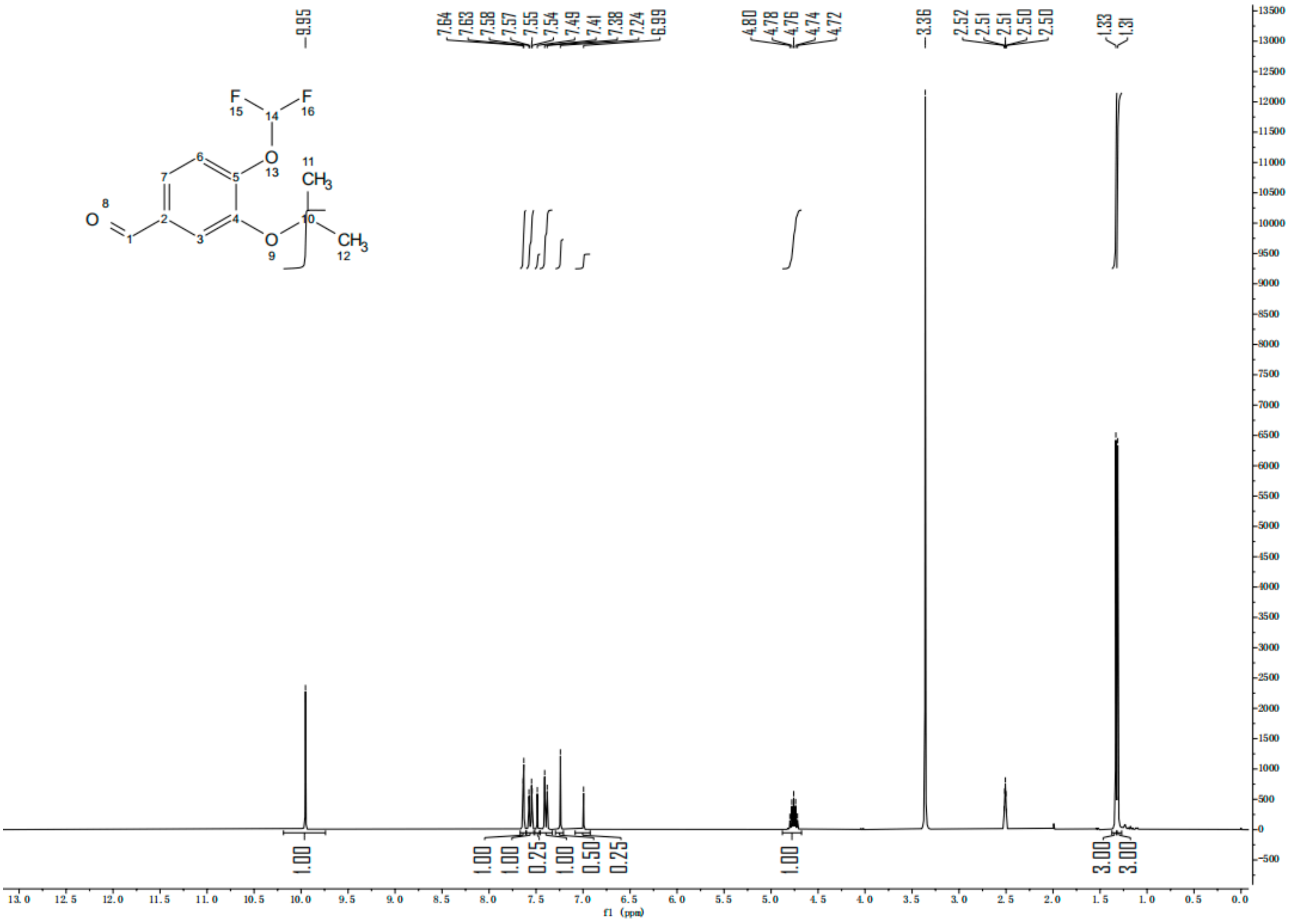

Figure S3.^{1}H-NMR Spectrum of 1t (300 MHz, DMSO-d6)

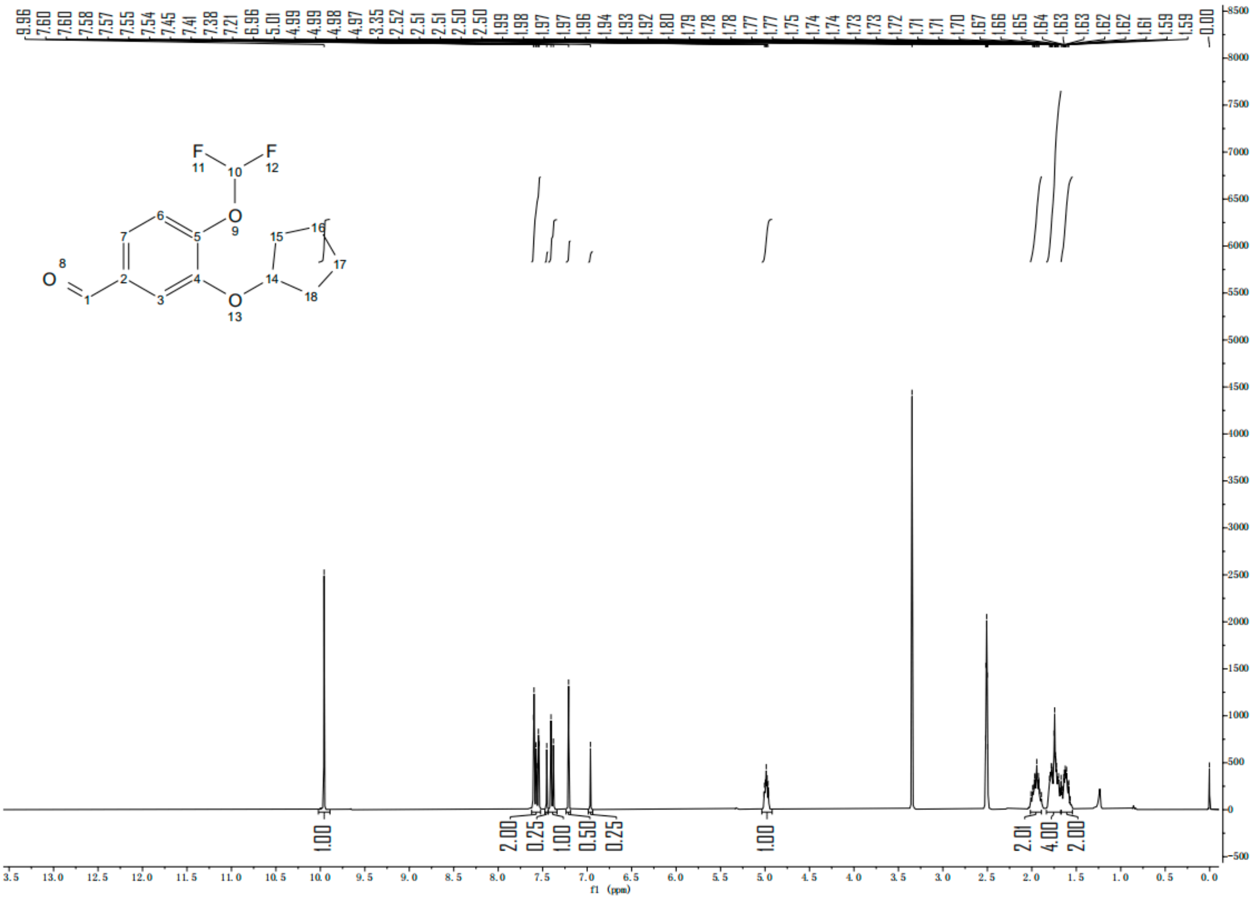

Figure S4.^{1}H-NMR Spectrum of 1u (300 MHz, DMSO-d6)

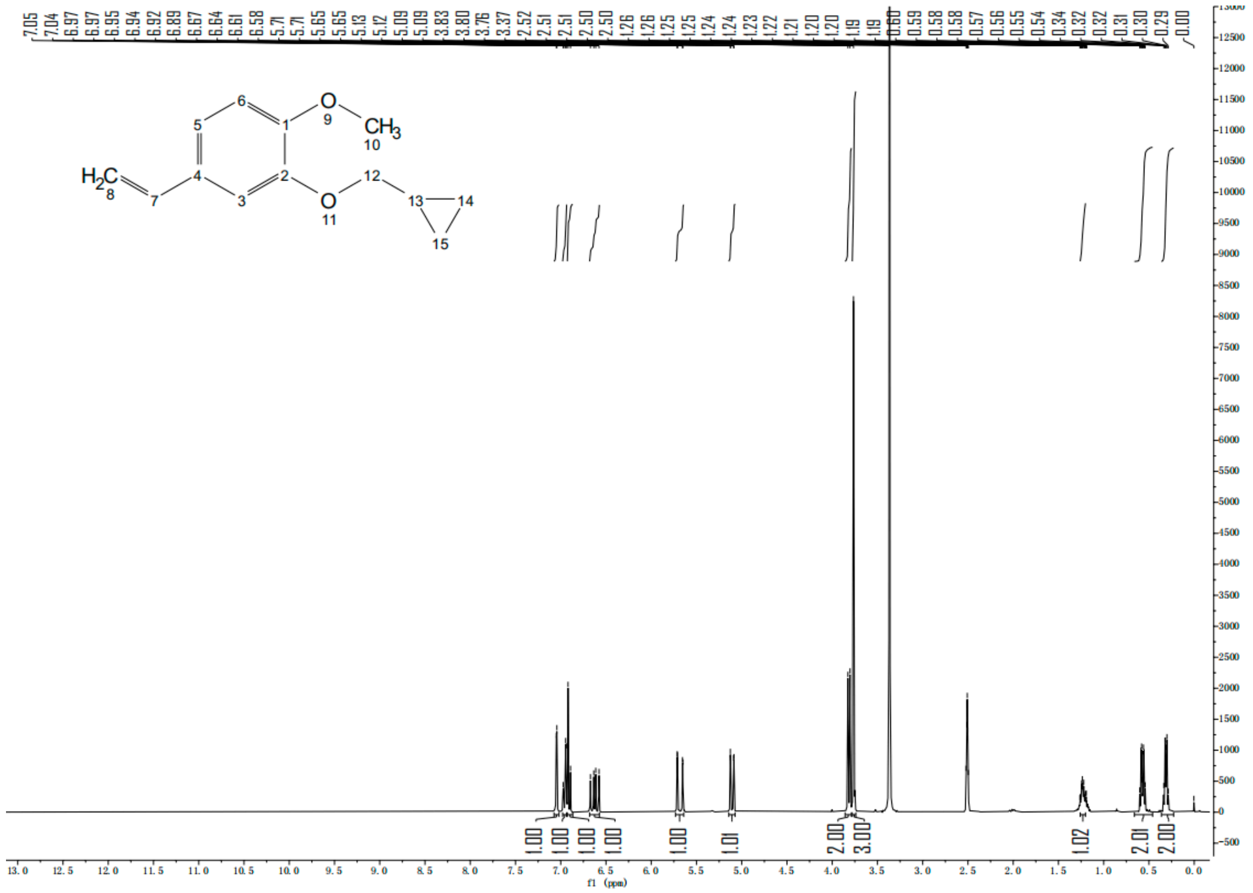

Figure S5.^{1}H-NMR Spectrum of 2a (300 MHz, DMSO-d6)

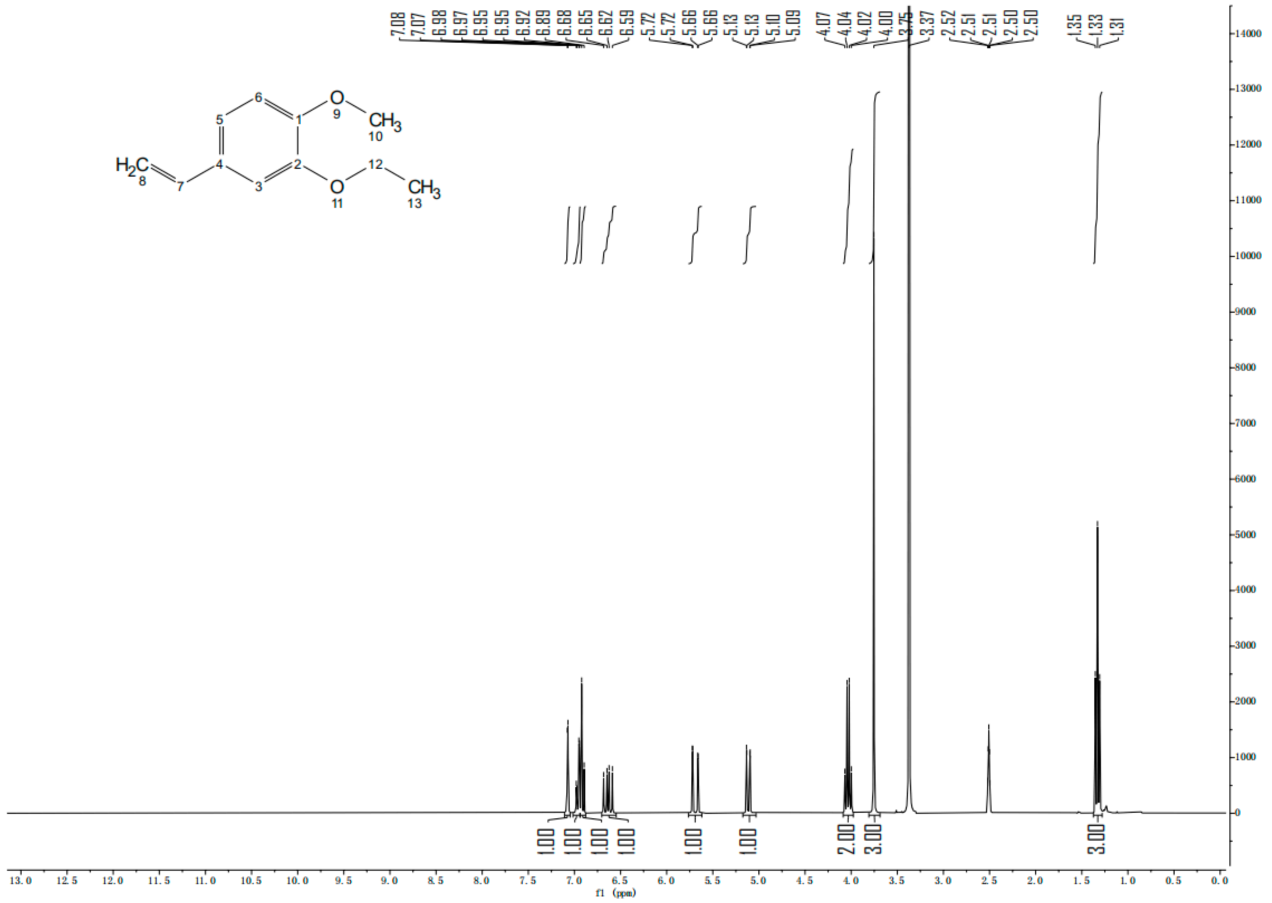

Figure S6.^{1}H-NMR Spectrum of 2b (300 MHz, DMSO-d6)

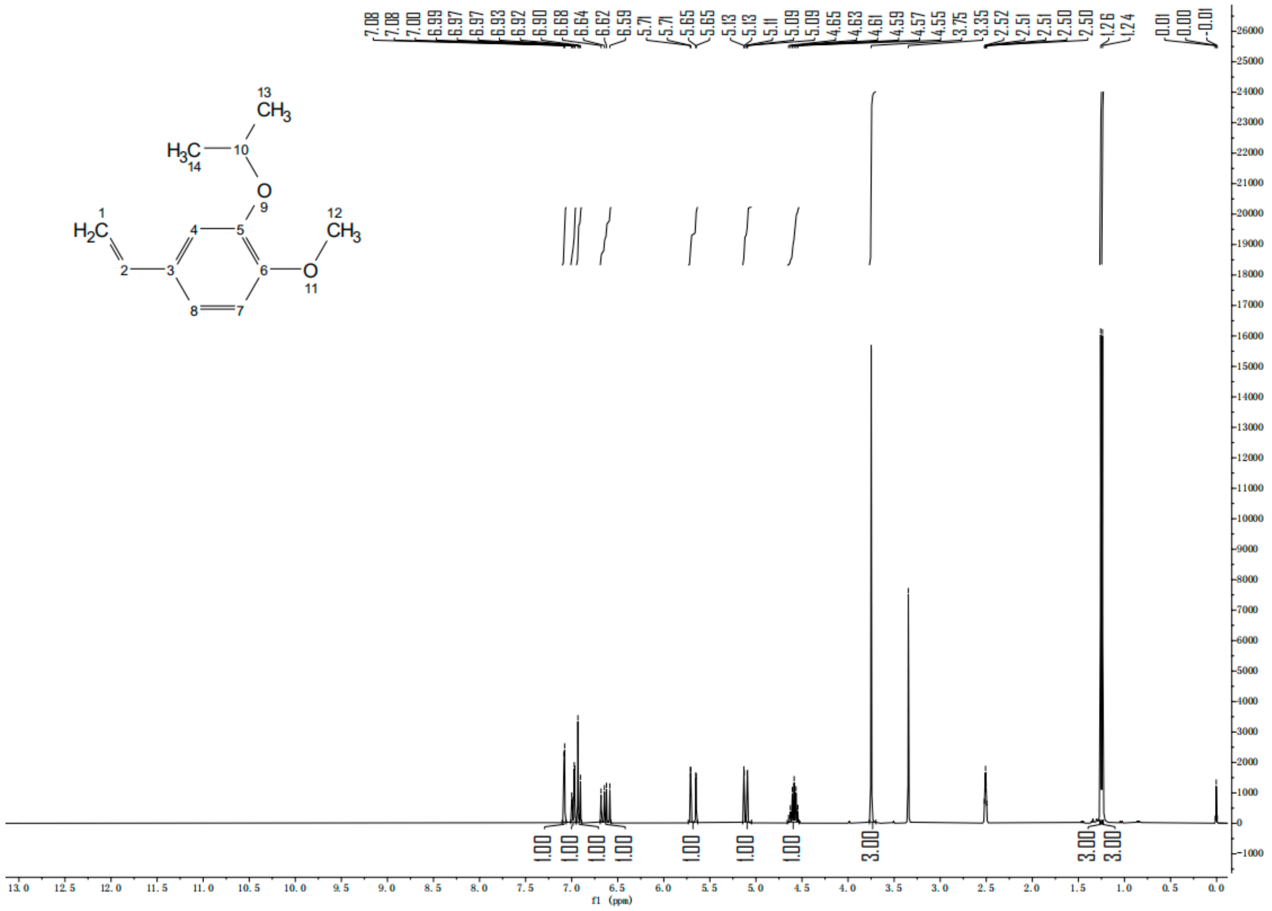

Figure S7.^{1}H-NMR Spectrum of 2c (300 MHz, DMSO-d6)

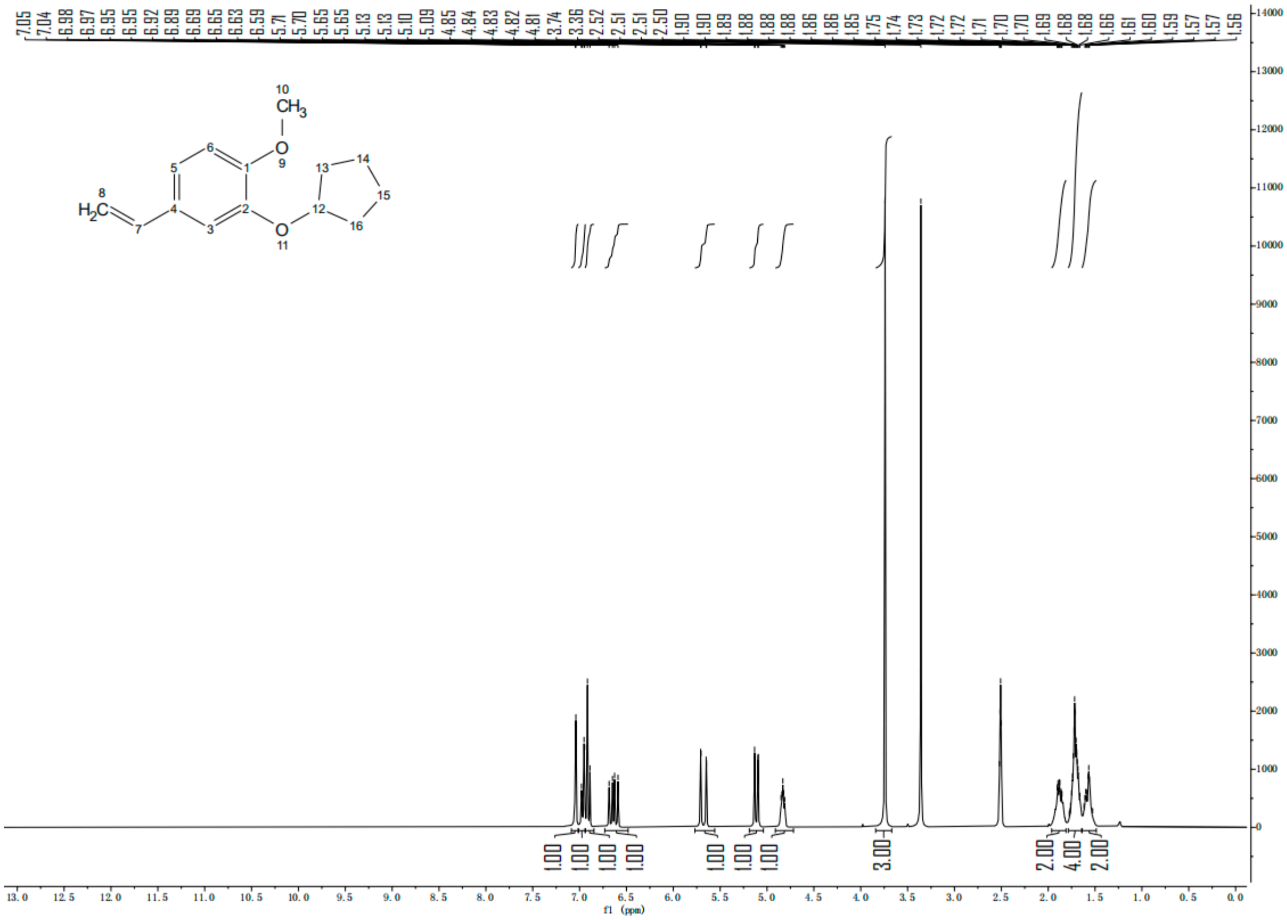

Figure S8.^{1}H-NMR Spectrum of 2d (300 MHz, DMSO-d6)

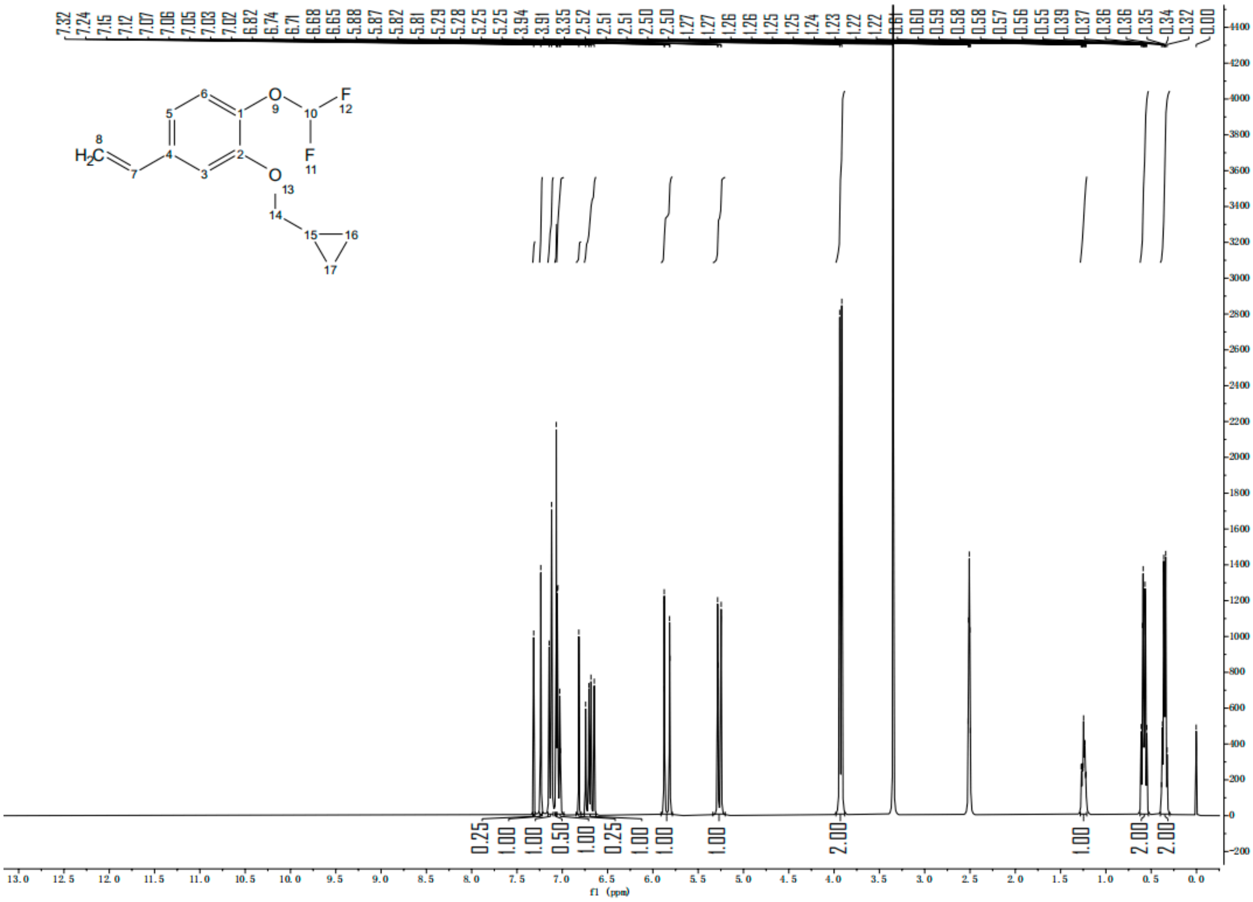

Figure S9.^{1}H-NMR Spectrum of 2e (300 MHz, DMSO-d6)

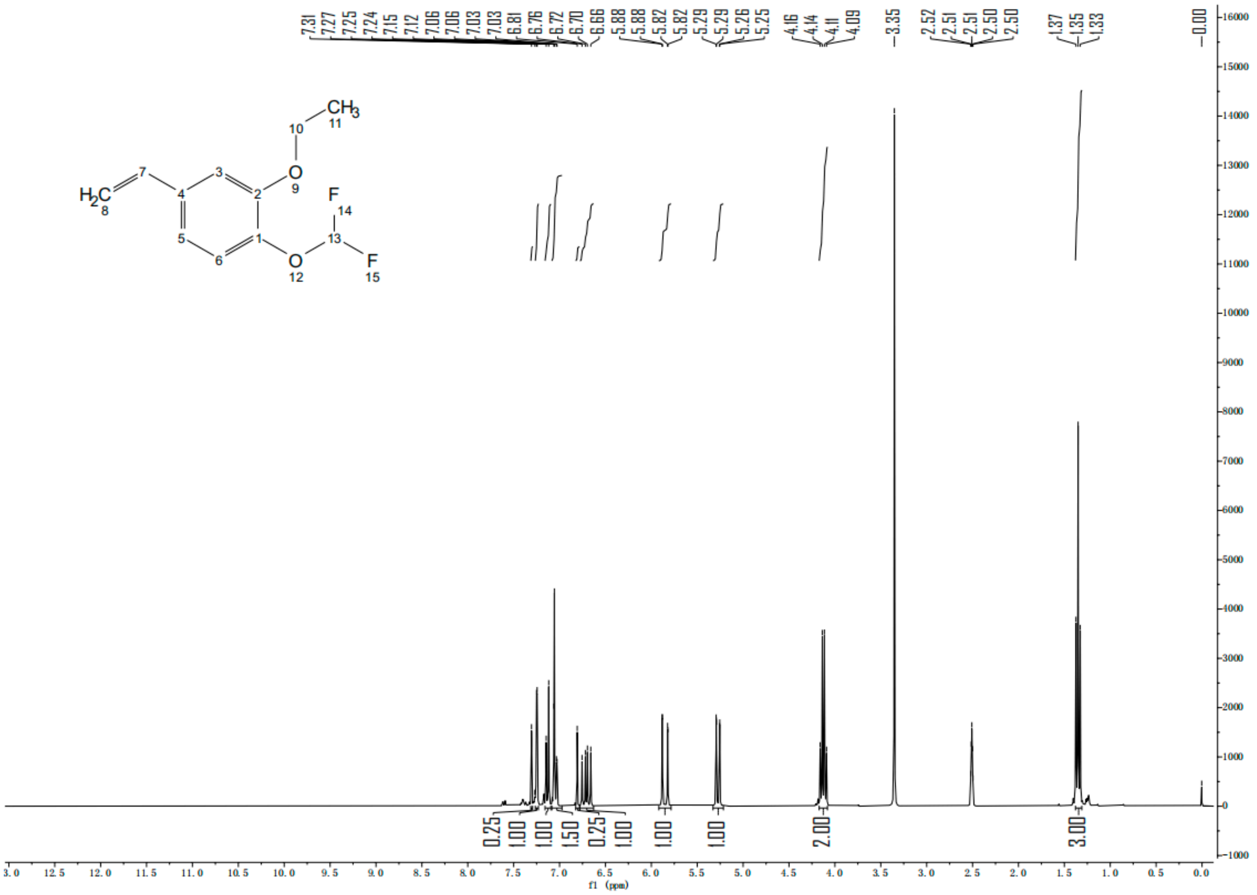

Figure S10.^{1}H-NMR Spectrum of 2f (300 MHz, DMSO-d6)

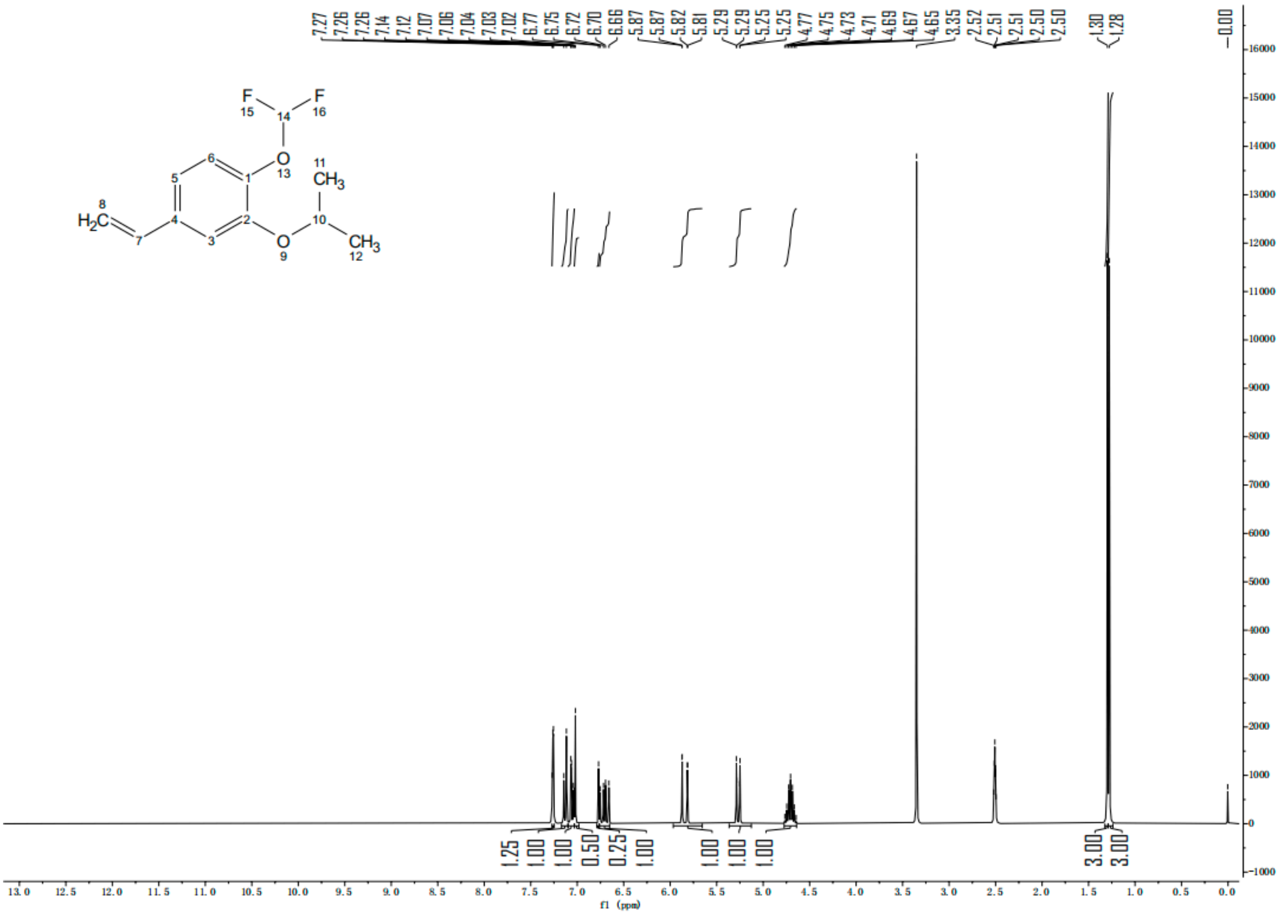

Figure S11.^{1}H-NMR Spectrum of 2g (300 MHz, DMSO-d6)

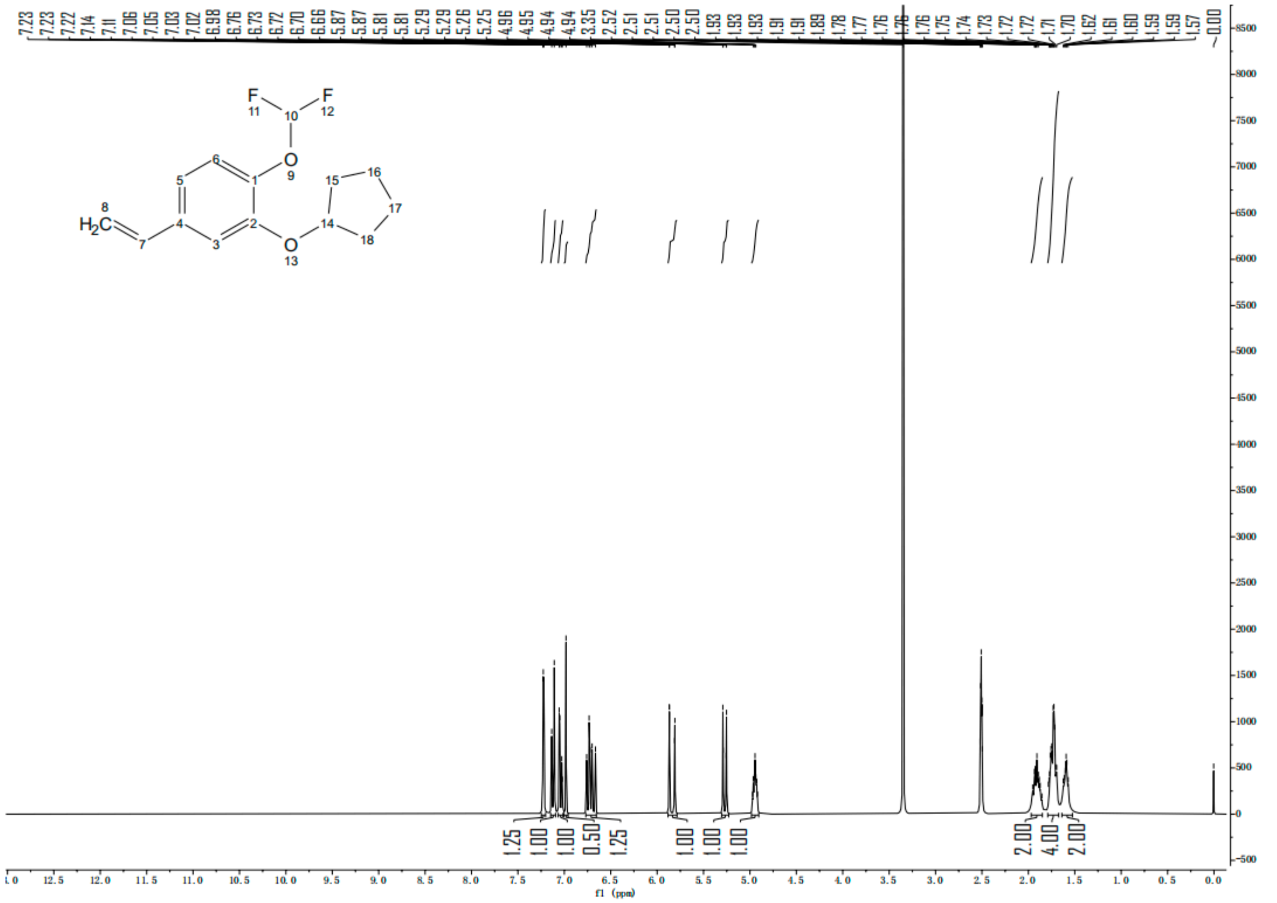

Figure S12.^{1}H-NMR Spectrum of 2h (300 MHz, DMSO-d6)

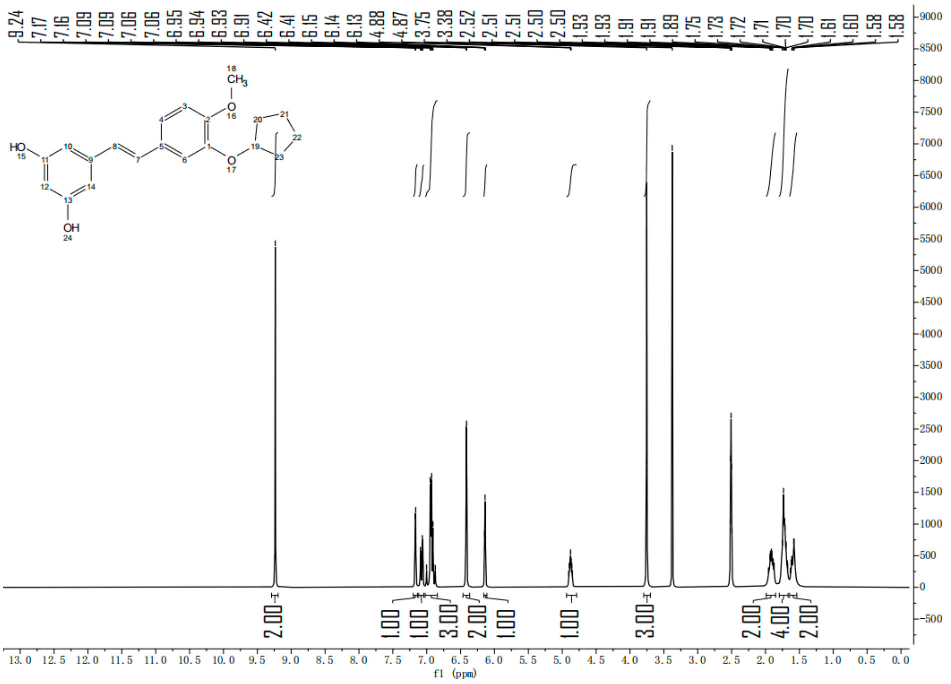

Figure S13.^{1}H-NMR Spectrum of 3a (300 MHz, DMSO-d6)

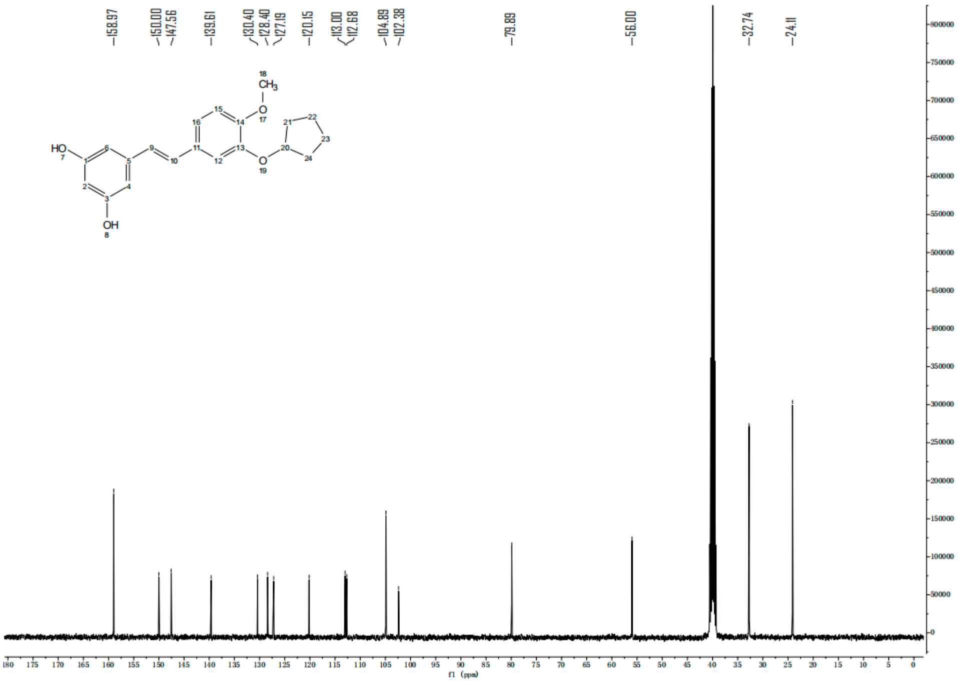

Figure S14.^{13}C-NMR Spectrum of the 3a (101 MHz, DMSO-d6)

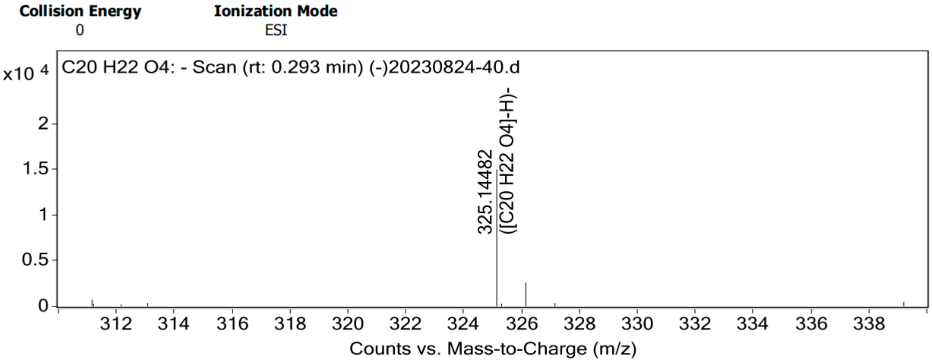

Figure S15.HRMS (ESI) Spectrum of the 3a

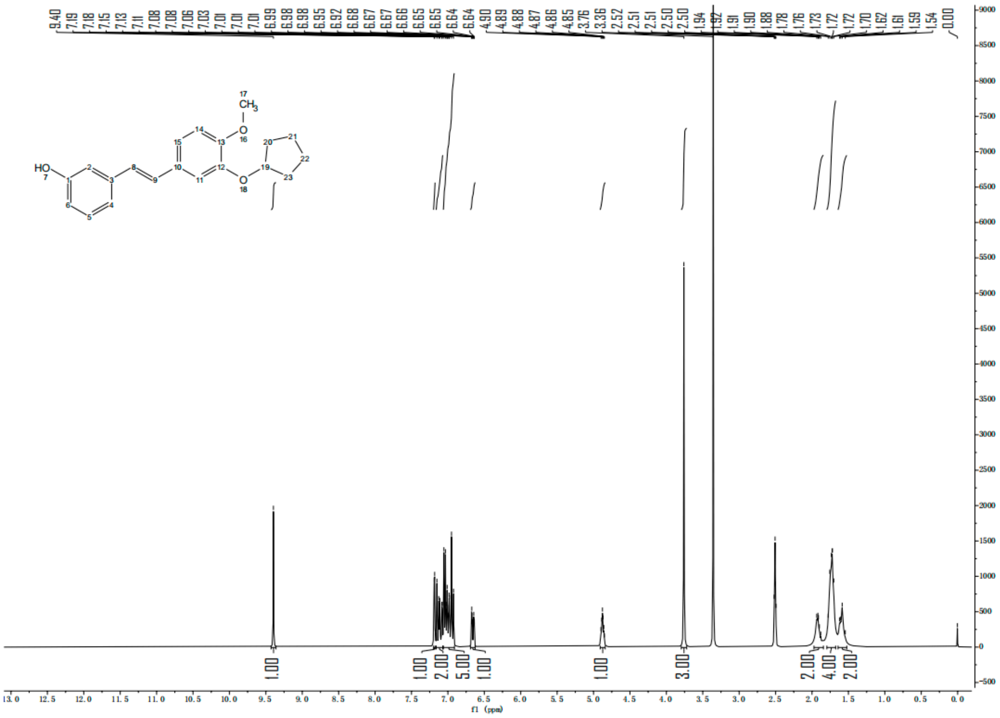

Figure S16.^{1}H-NMR Spectrum of 3b (300 MHz, DMSO-d6)

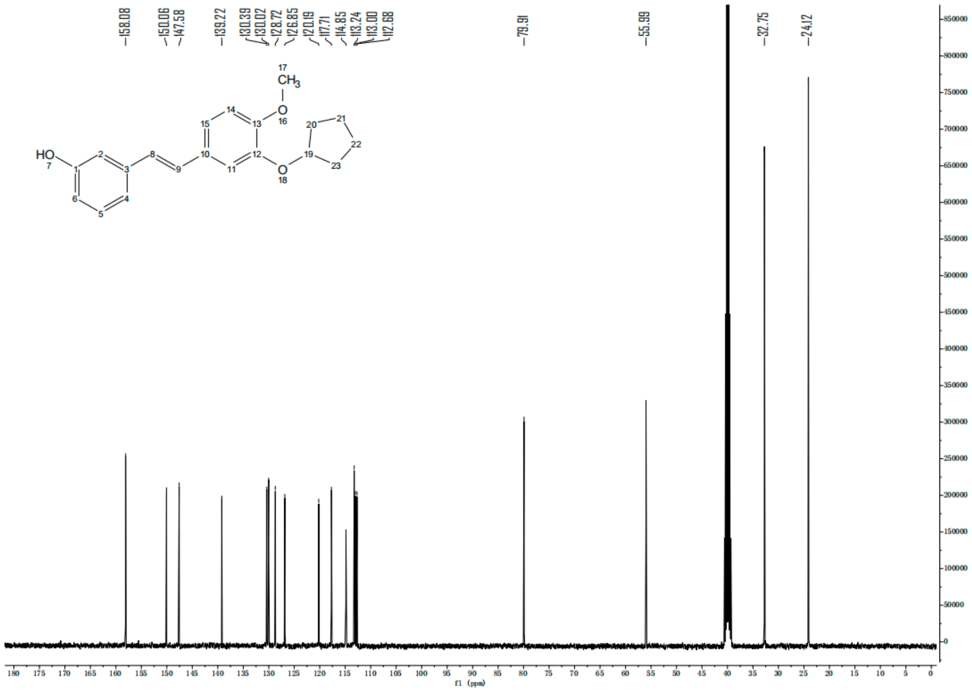

Figure S17.^{13}C-NMR Spectrum of the 3b (101 MHz, DMSO-d6)

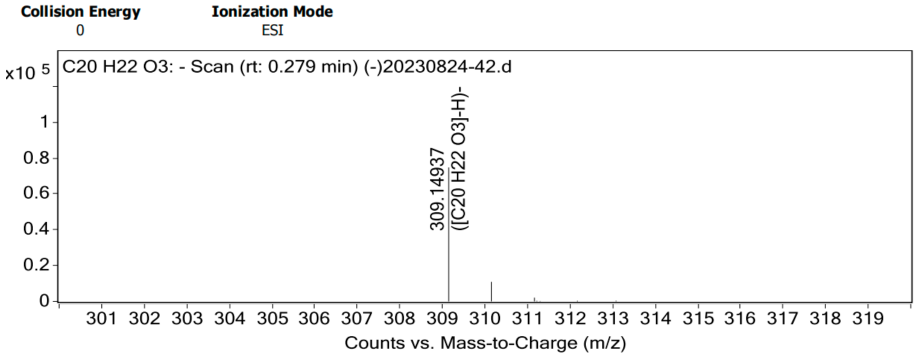

Figure S18.HRMS (ESI) Spectrum of the 3b

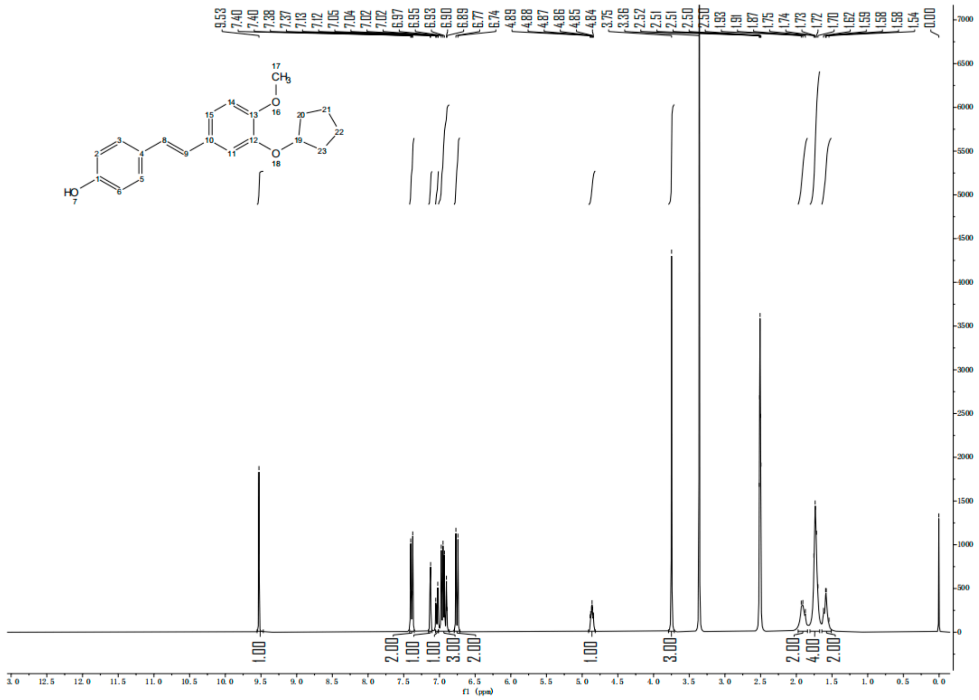

Figure S19.^{1}H-NMR Spectrum of 3c (300 MHz, DMSO-d6)

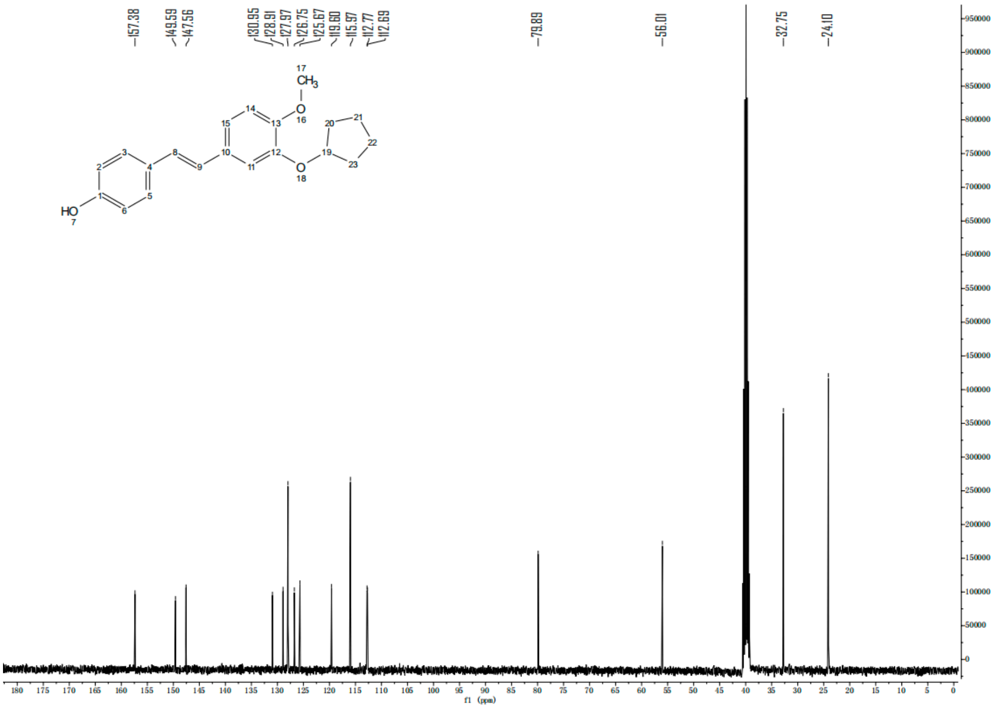

Figure S20.^{13}C-NMR Spectrum of the 3c (101 MHz, DMSO-d6)

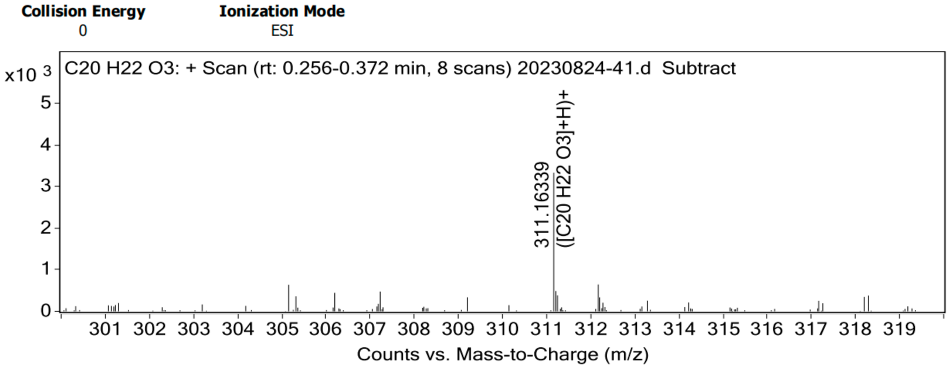

Figure S21.HRMS (ESI) Spectrum of the 3c

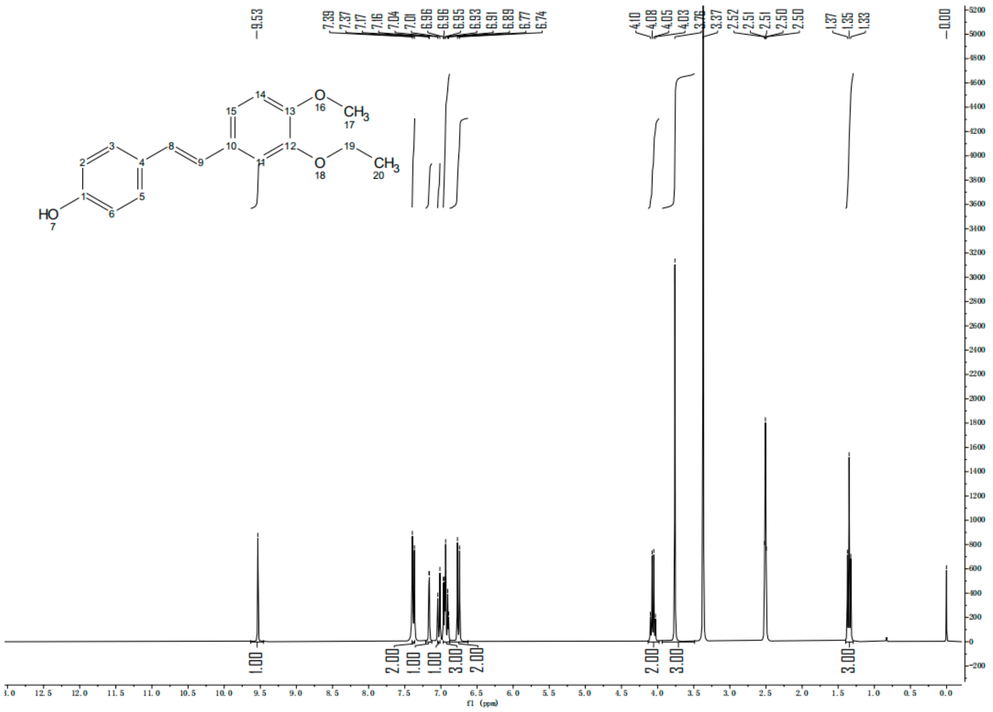

Figure S22.^{1}H-NMR Spectrum of 3d (300 MHz, DMSO-d6)

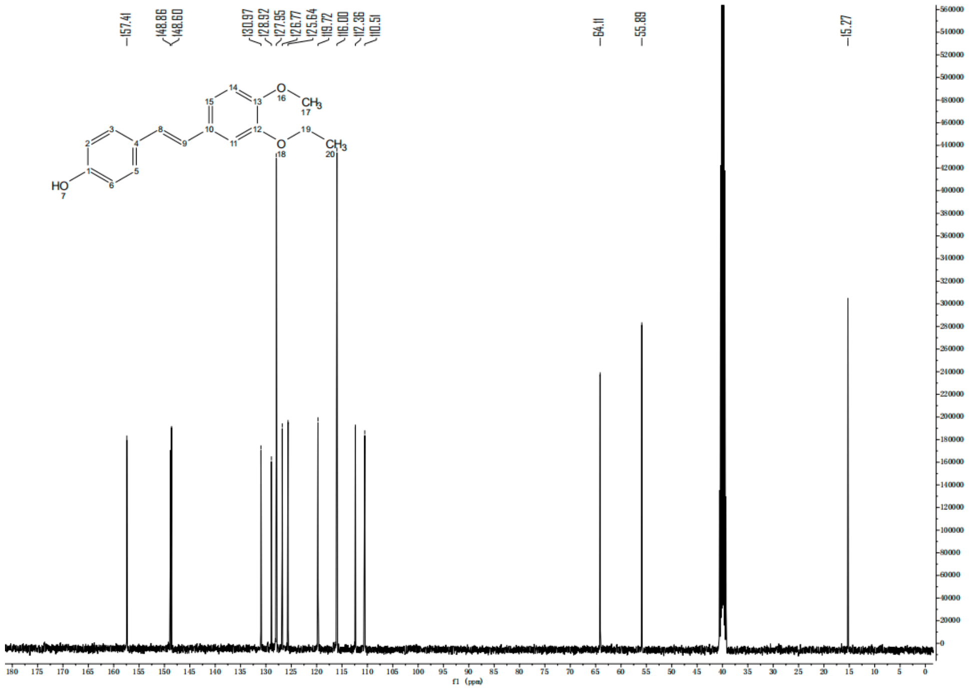

Figure S23.^{13}C-NMR Spectrum of the 3d (101 MHz, DMSO-d6)

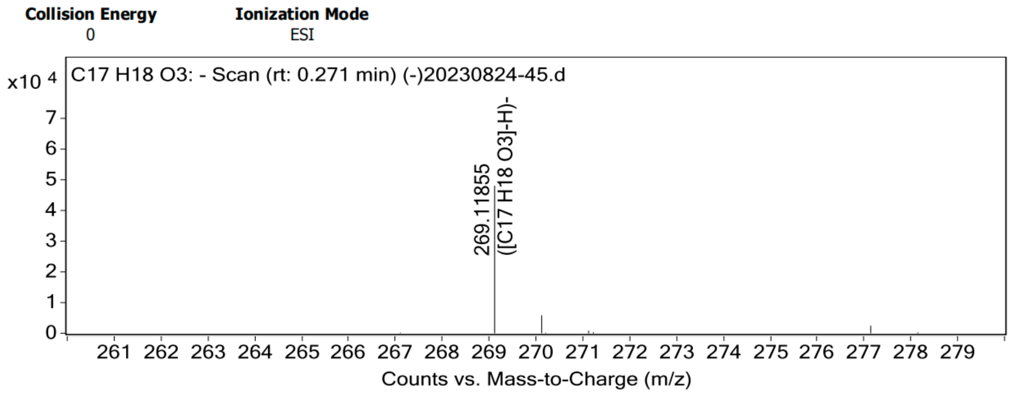

Figure S24.HRMS (ESI) Spectrum of the 3d

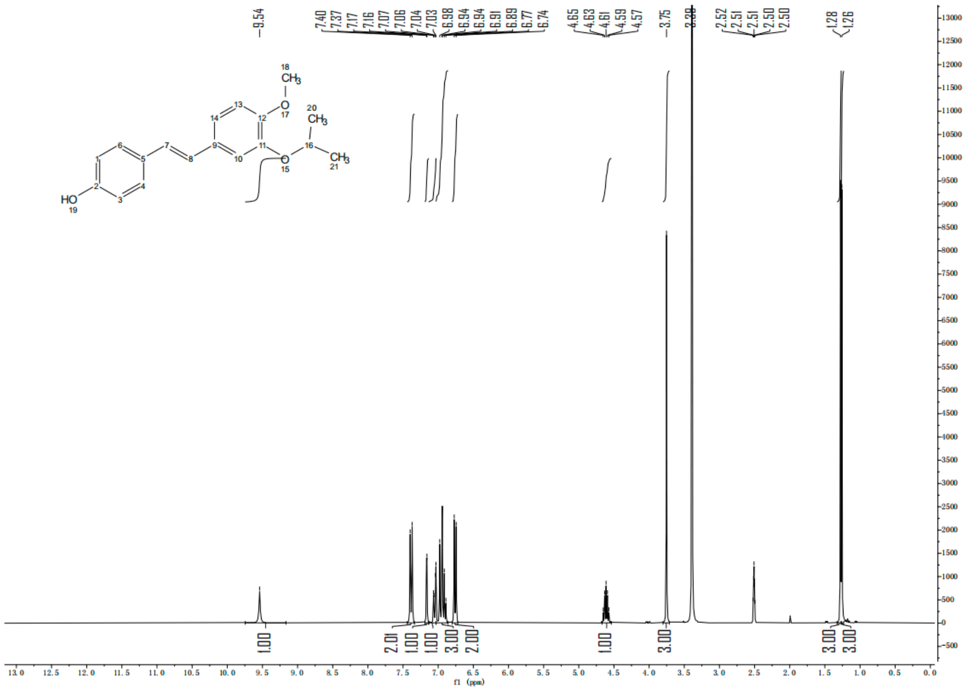

Figure S25.^{1}H-NMR Spectrum of 3e (300 MHz, DMSO-d6)

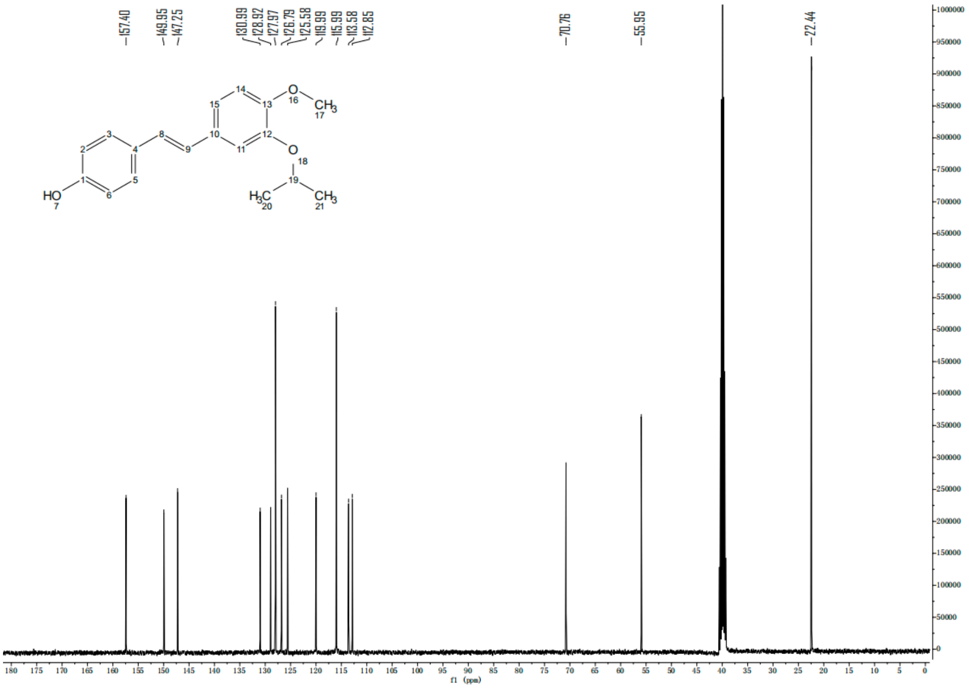

Figure S26.^{13}C-NMR Spectrum of the 3e (101 MHz, DMSO-d6)

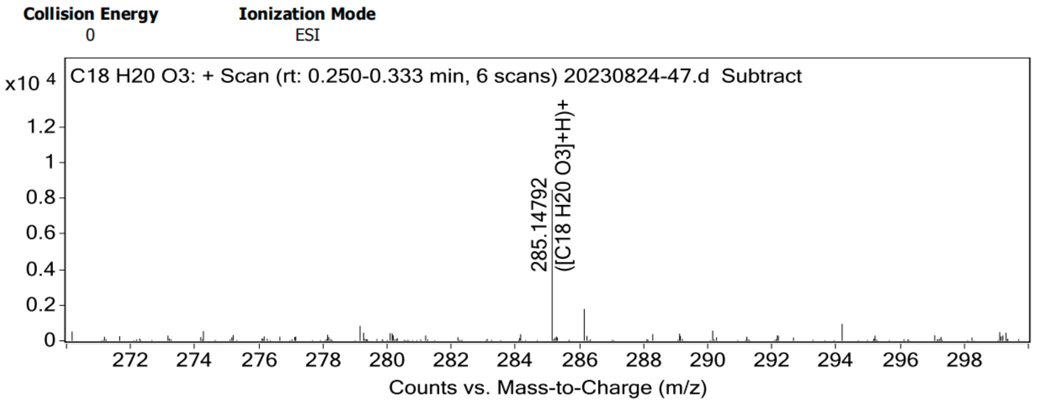

Figure S27.HRMS (ESI) Spectrum of the 3e

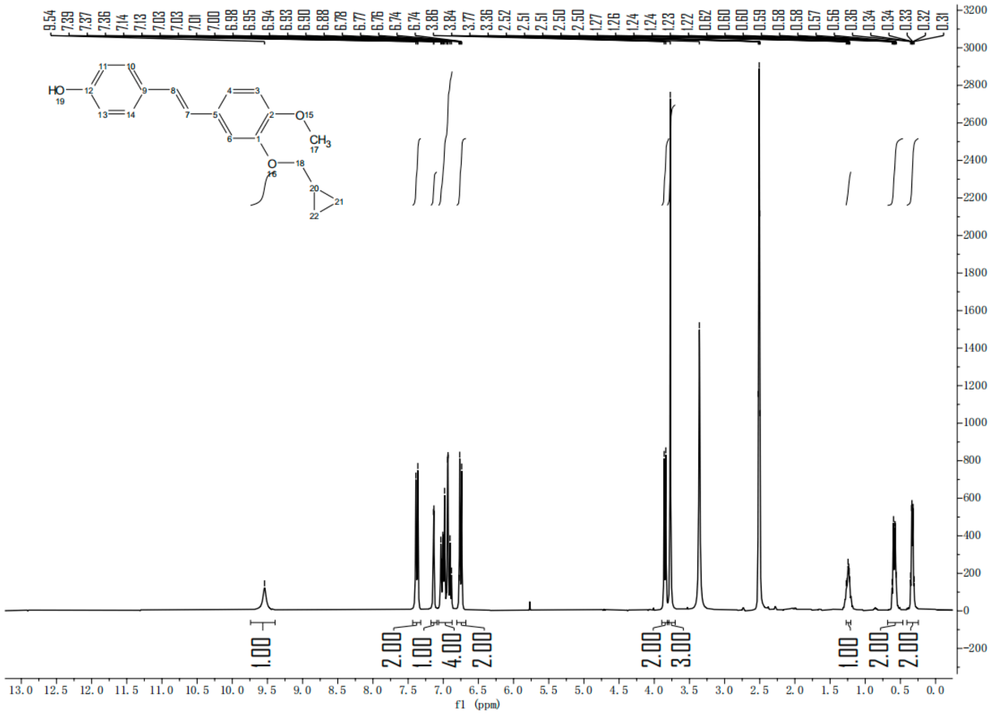

Figure S28.^{1}H-NMR Spectrum of 3f (300 MHz, DMSO-d6)

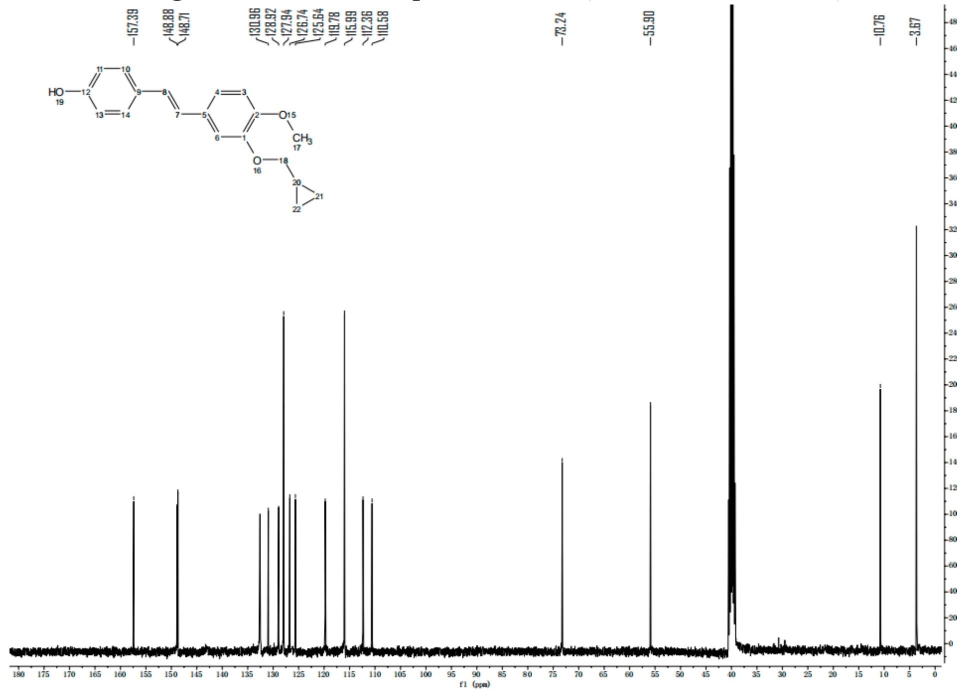

Figure S29.^{13}C-NMR Spectrum of the 3f (101 MHz, DMSO-d6)

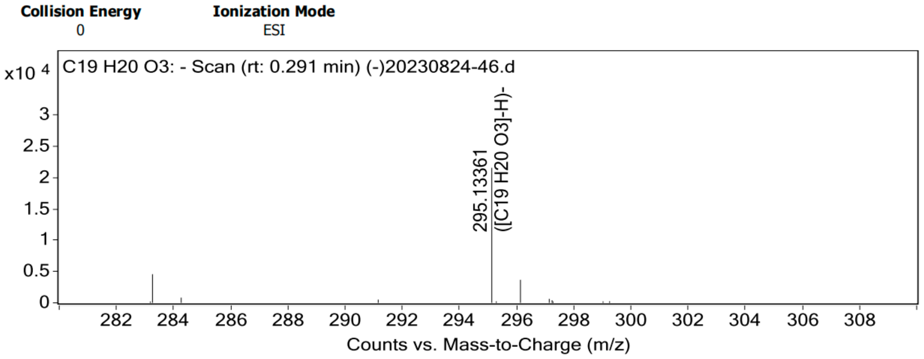

Figure S30.HRMS (ESI) Spectrum of the 3f

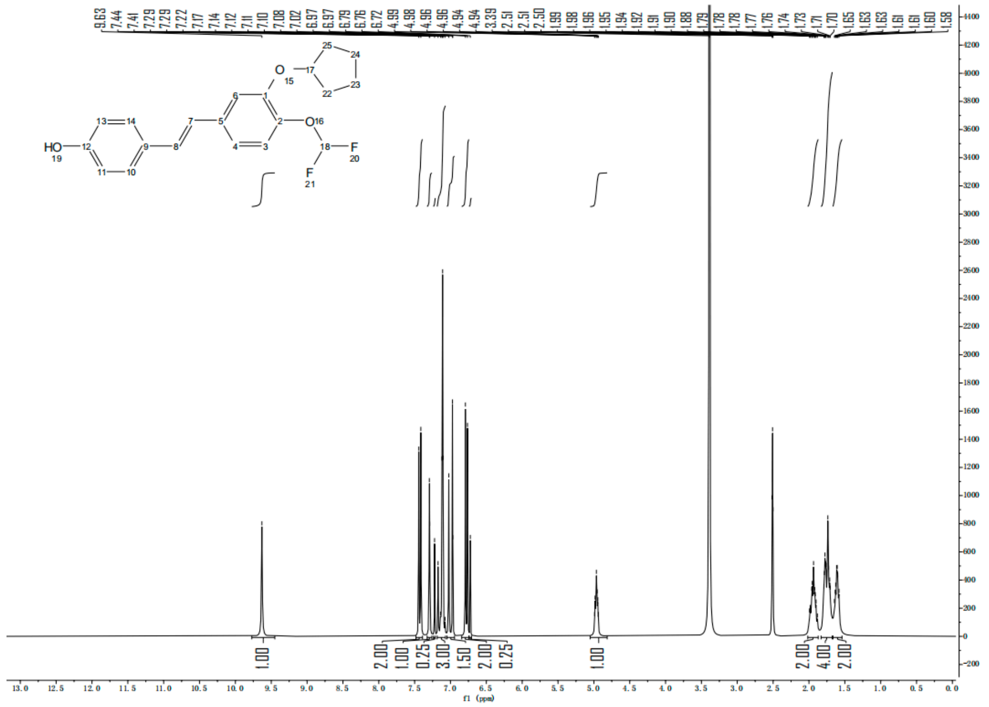

Figure S31.^{1}H-NMR Spectrum of 3g (300 MHz, DMSO-d6)

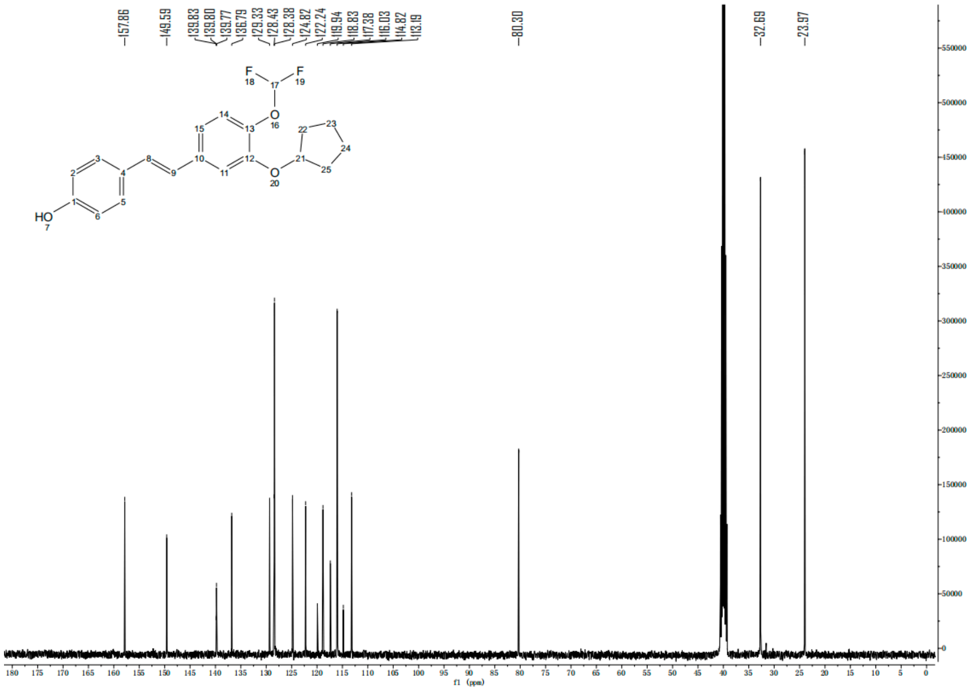

Figure S32.^{13}C-NMR Spectrum of the 3g (101 MHz, DMSO-d6)

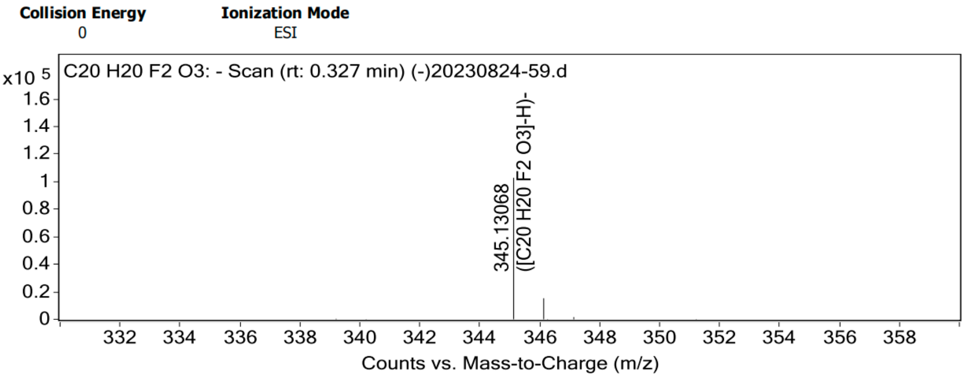

Figure S33.HRMS (ESI) Spectrum of the 3g

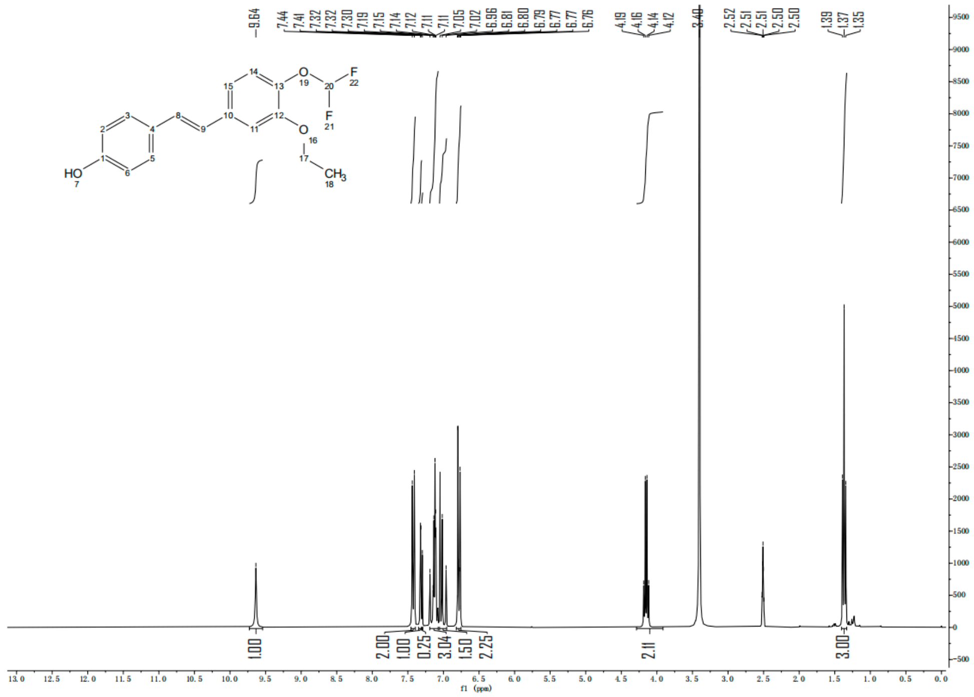

Figure S34.^{1}H-NMR Spectrum of 3h (300 MHz, DMSO-d6)

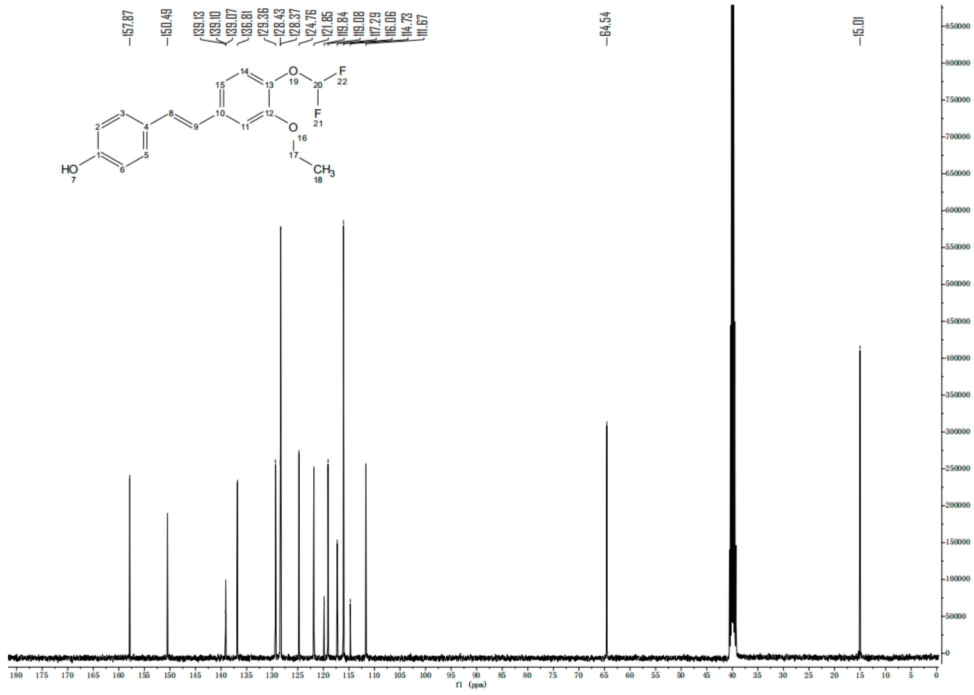

Figure S35.^{13}C-NMR Spectrum of the 3h (101 MHz, DMSO-d6)

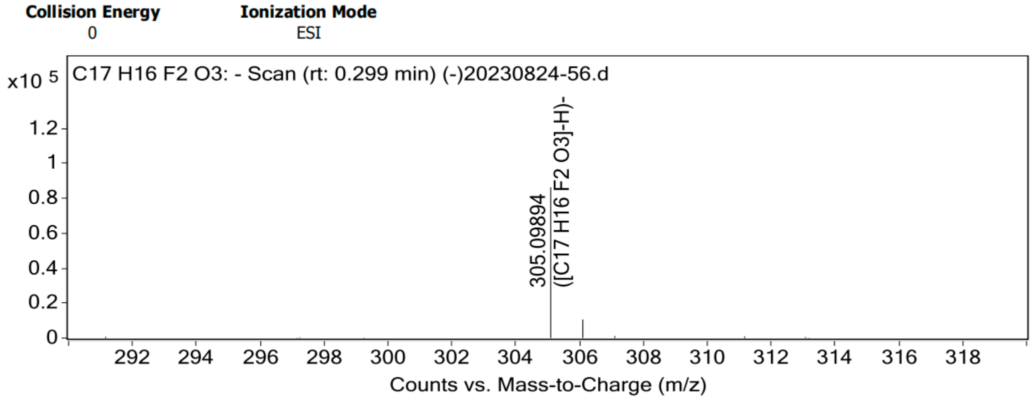

Figure S36.HRMS (ESI) Spectrum of the 3h

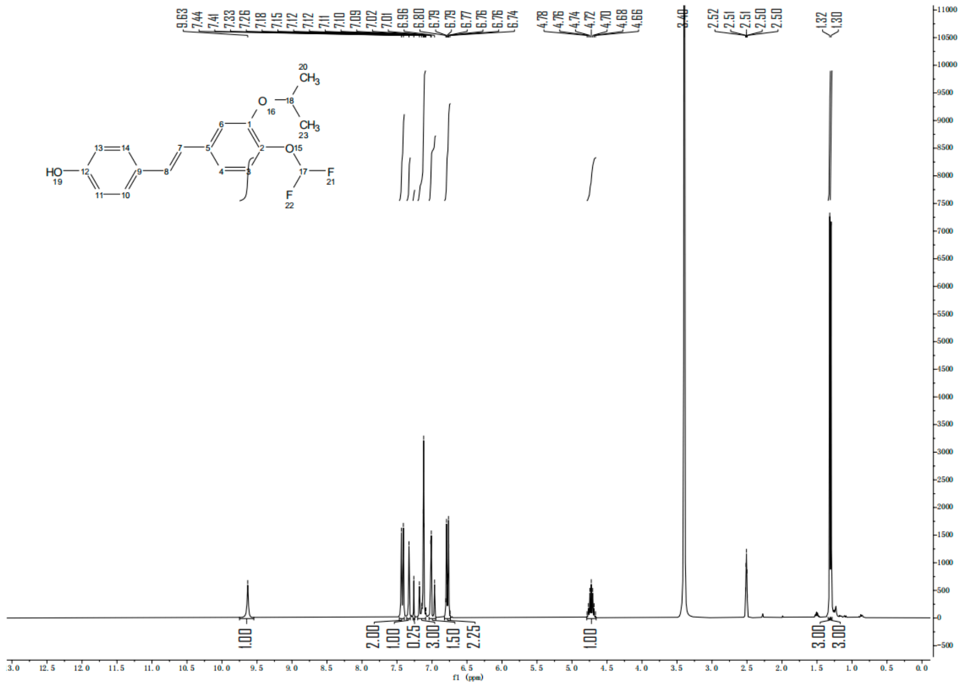

Figure S37.¹H-NMR Spectrum of 3i (300 MHz, DMSO-d6)

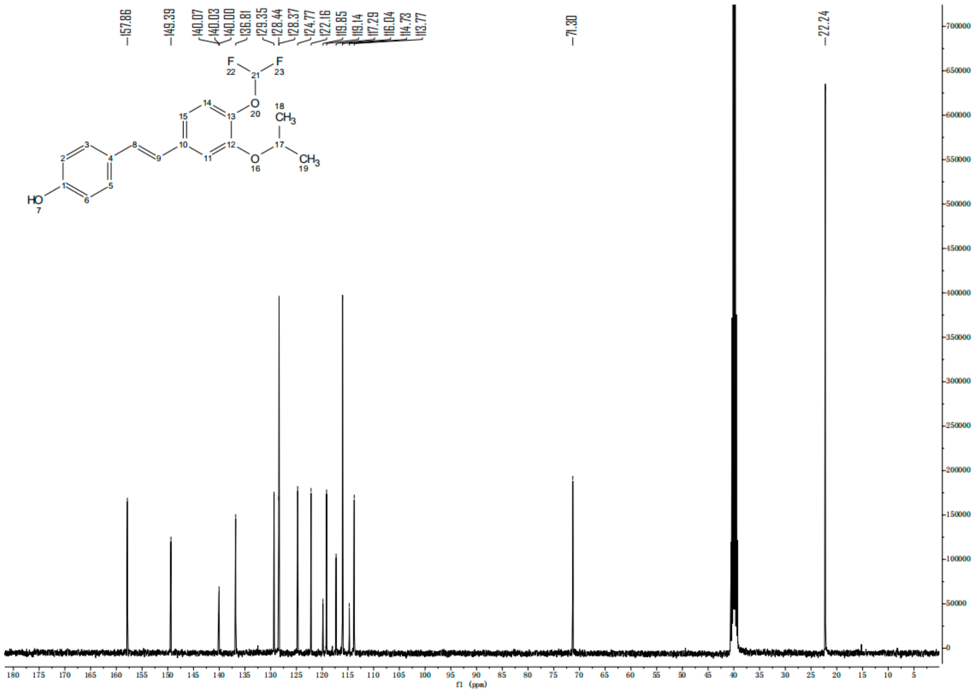

Figure S38.¹³C-NMR Spectrum of the 3i (101 MHz, DMSO-d6)

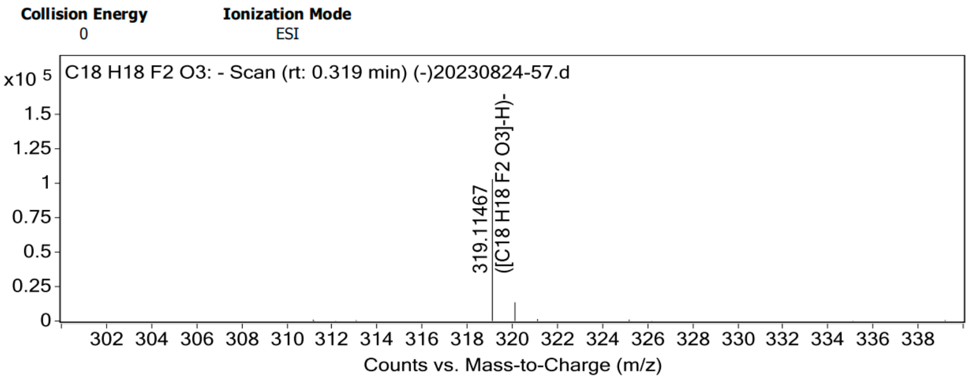

Figure S39.HRMS (ESI) Spectrum of the 3i

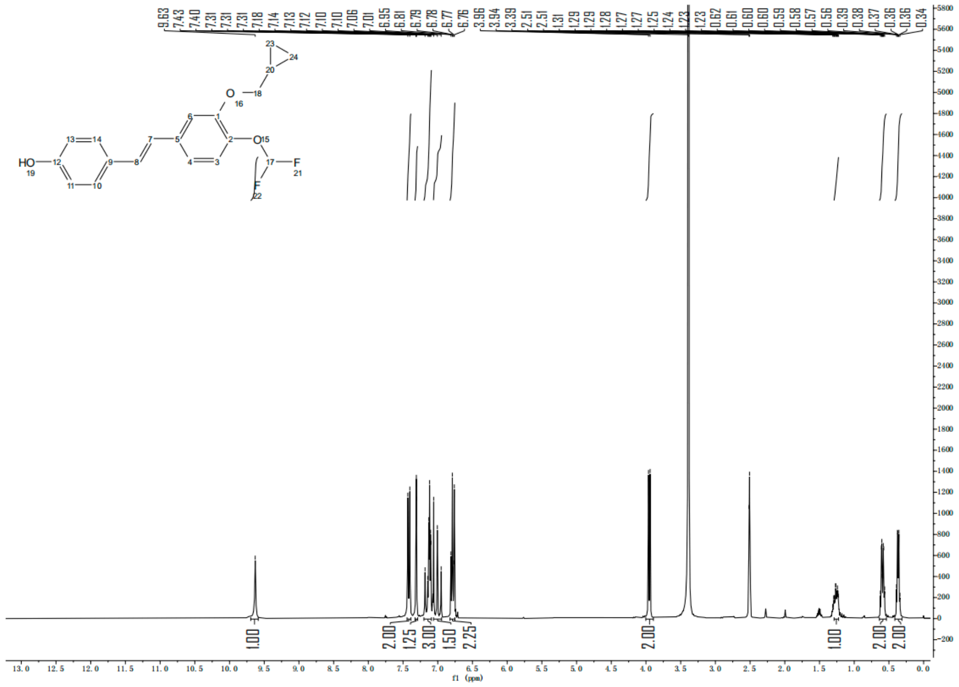

Figure S40.^{1}H-NMR Spectrum of 3j (300 MHz, DMSO-d6)

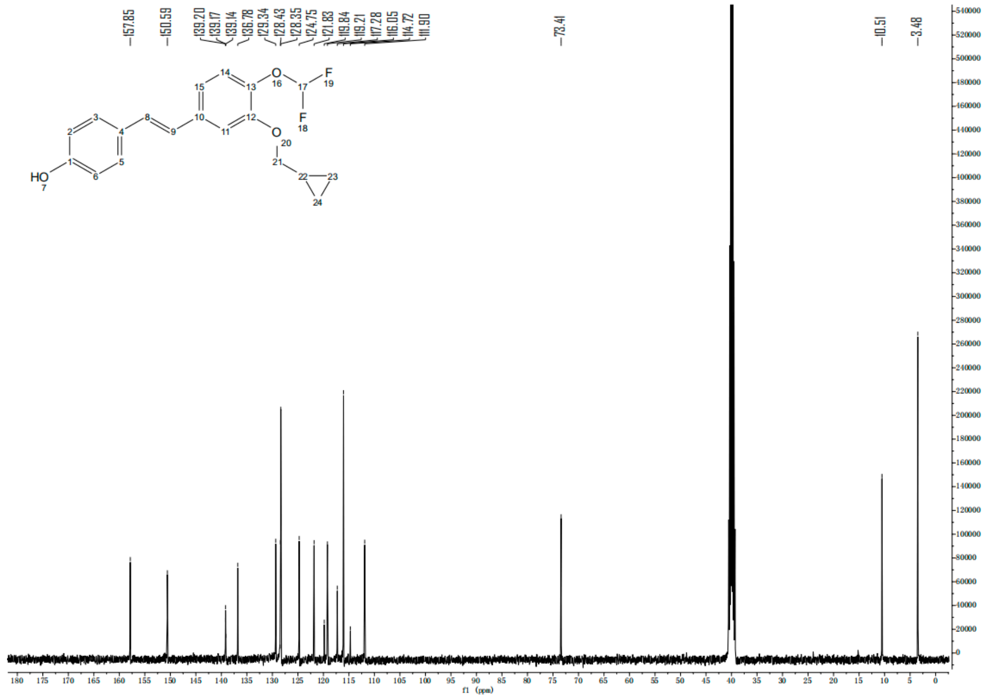

Figure S41.^{13}C-NMR Spectrum of the 3j (101 MHz, DMSO-d6)

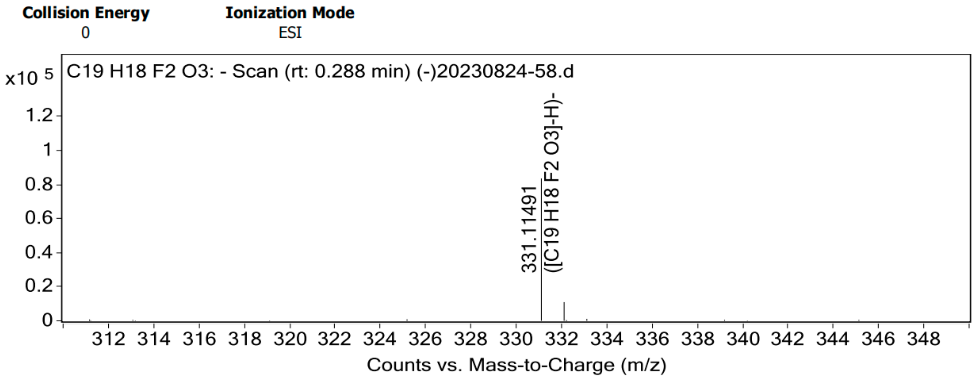

Figure S42.HRMS (ESI) Spectrum of the 3j

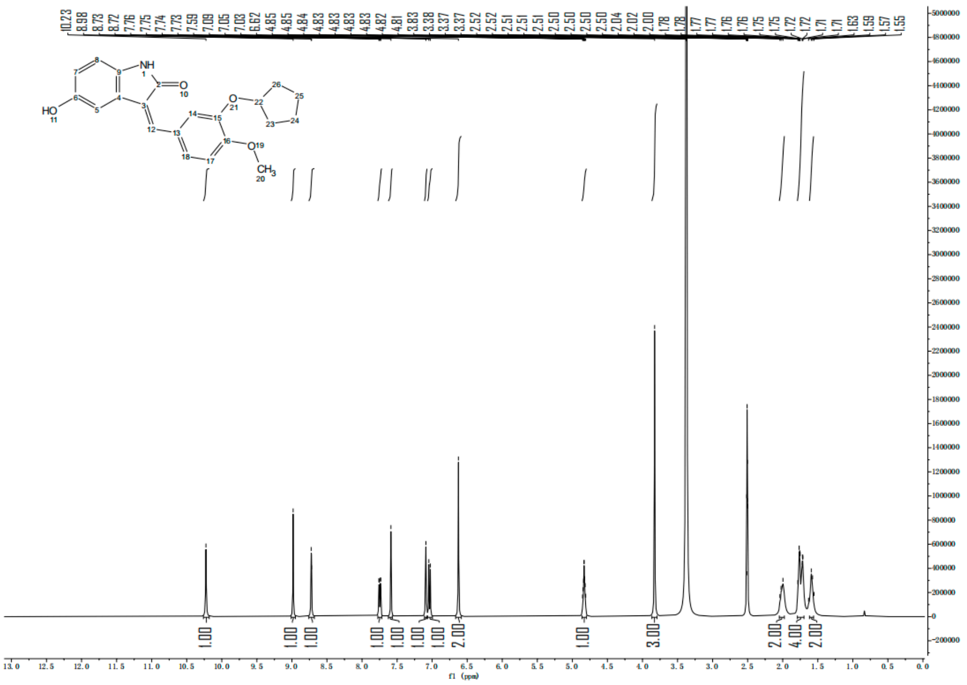

Figure S43.^{1}H-NMR Spectrum of 4a (400 MHz, DMSO-d6)

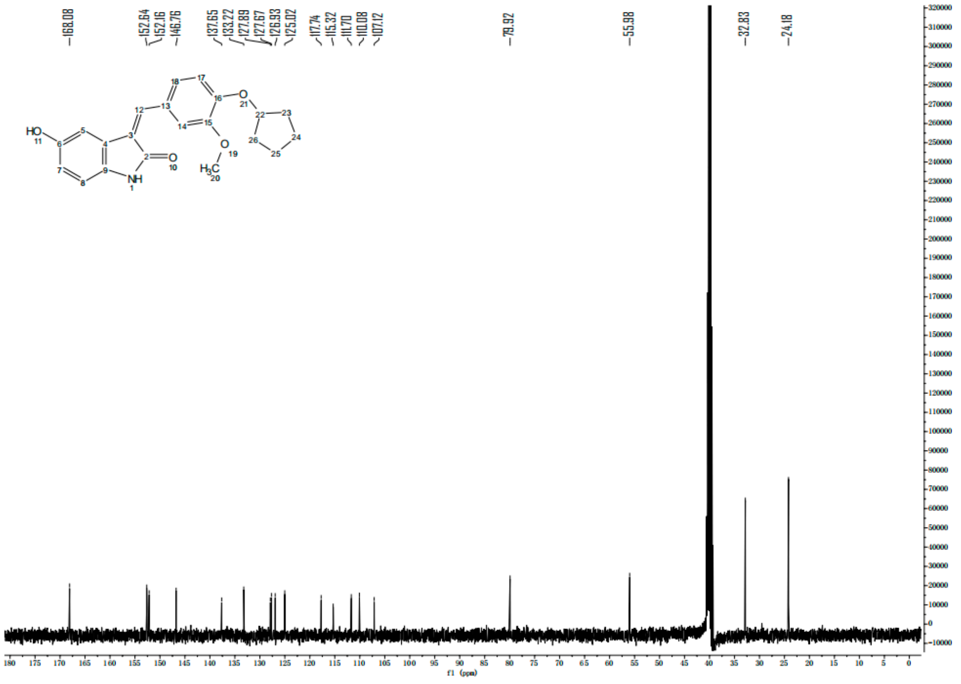

Figure S44.^{13}C-NMR Spectrum of the 4a (101 MHz, DMSO-d6)

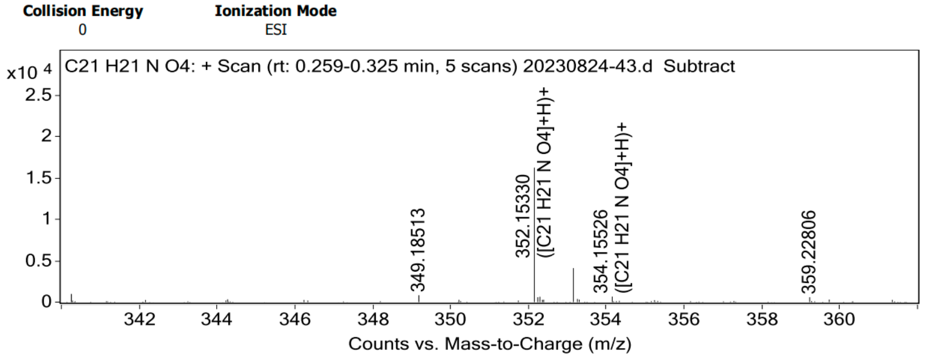

Figure S45.HRMS (ESI) Spectrum of the 4a

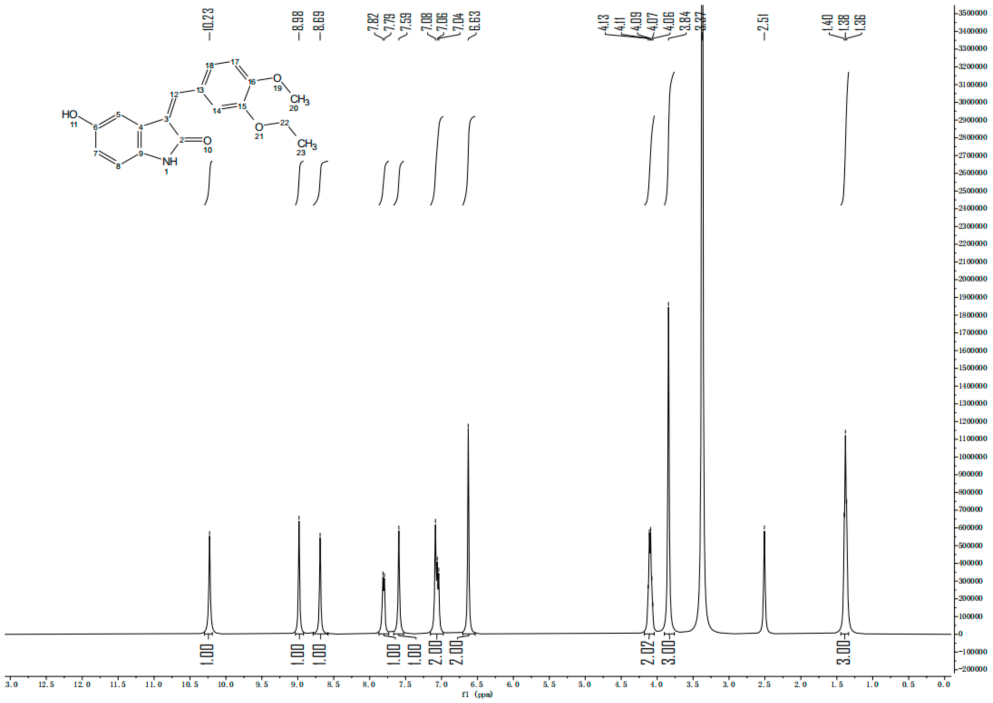

Figure S46.¹H-NMR Spectrum of the 4b (400 MHz, DMSO-d6)

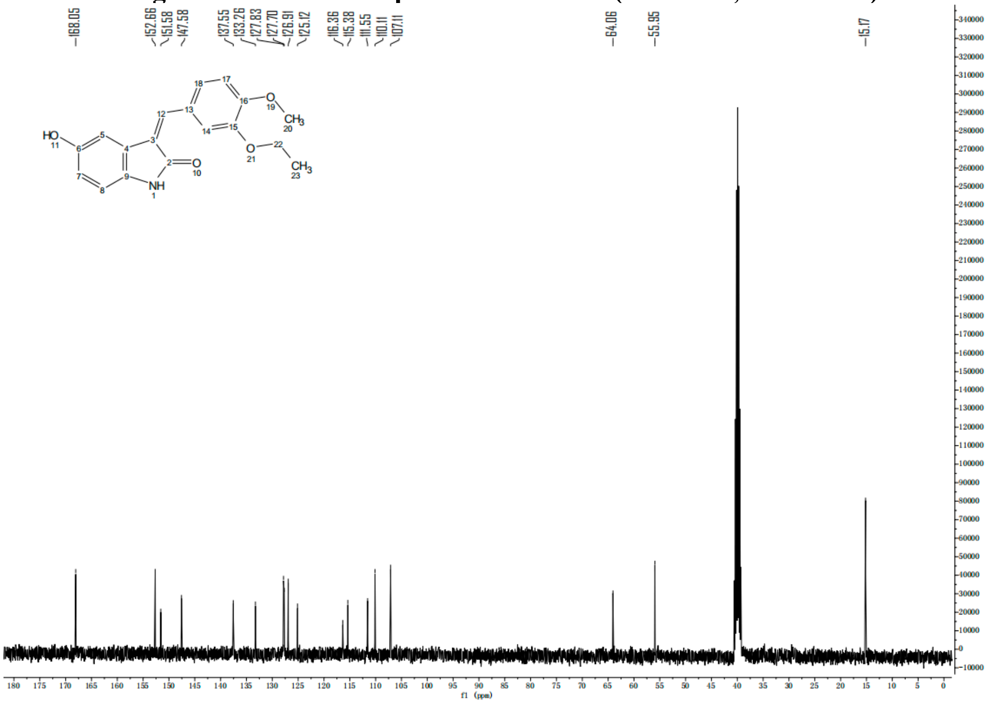

Figure S47.¹³C-NMR Spectrum of the 4b (101 MHz, DMSO-d6)

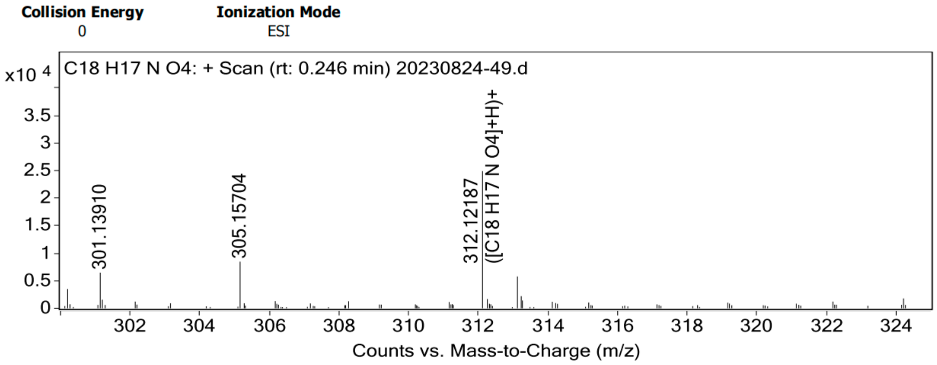

Figure S48.HRMS (ESI) Spectrum of the 4b

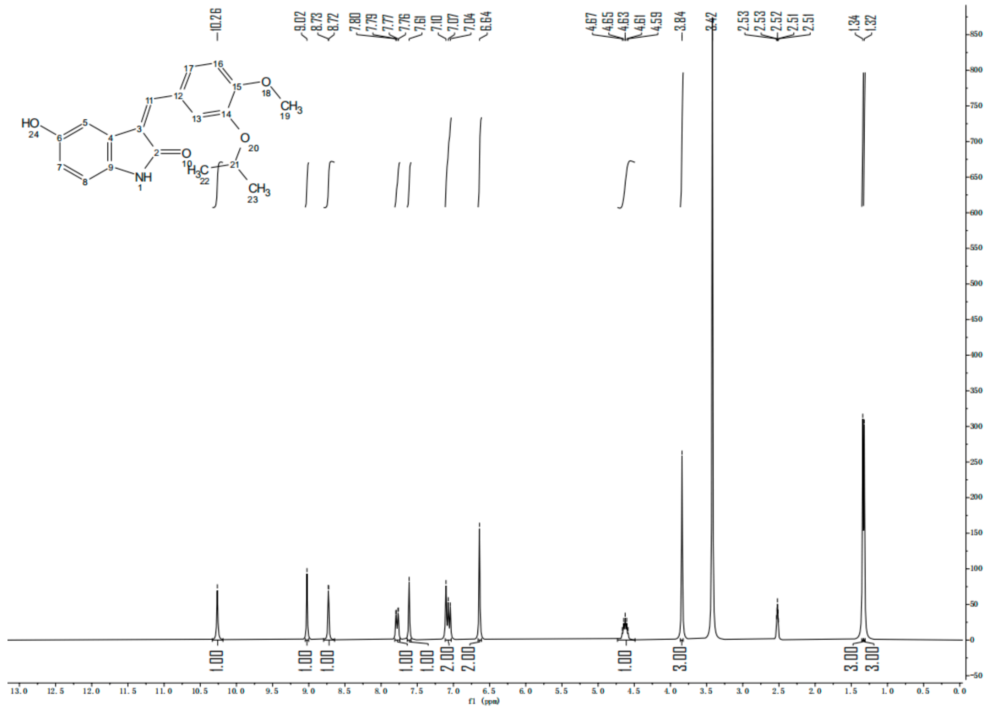

Figure S49.^{1}H-NMR Spectrum of 4c (300 MHz, DMSO-d6)

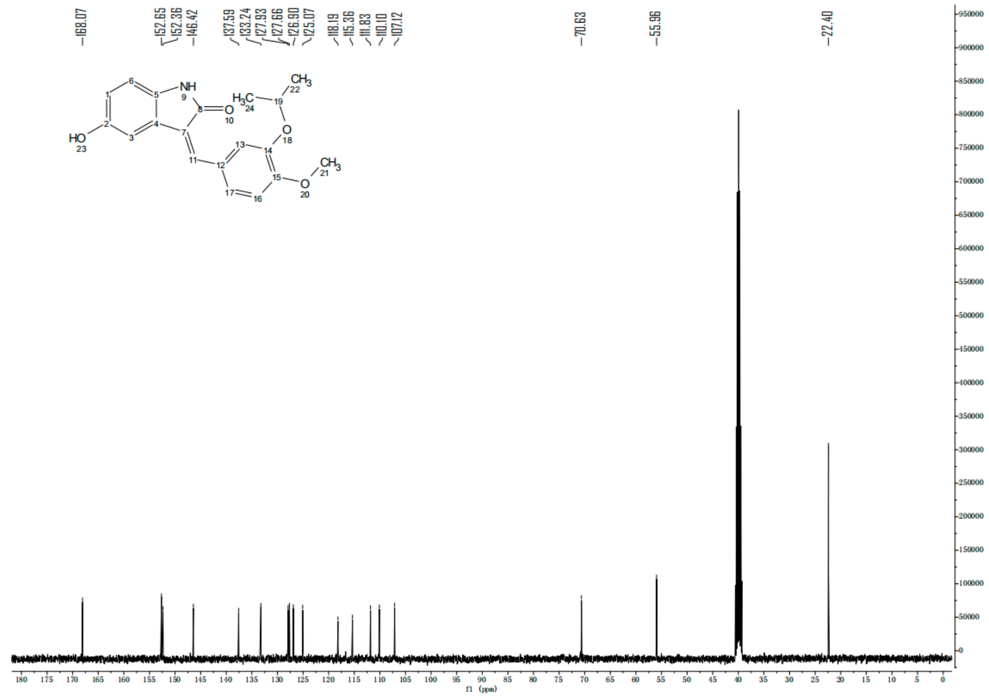

Figure S50.^{13}C-NMR Spectrum of the 4c (101 MHz, DMSO-d6)

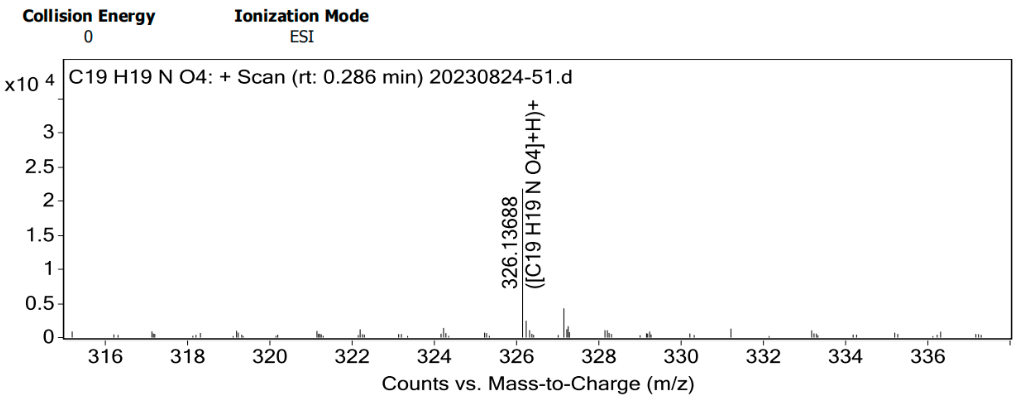

Figure S51.HRMS (ESI) Spectrum of the 4c

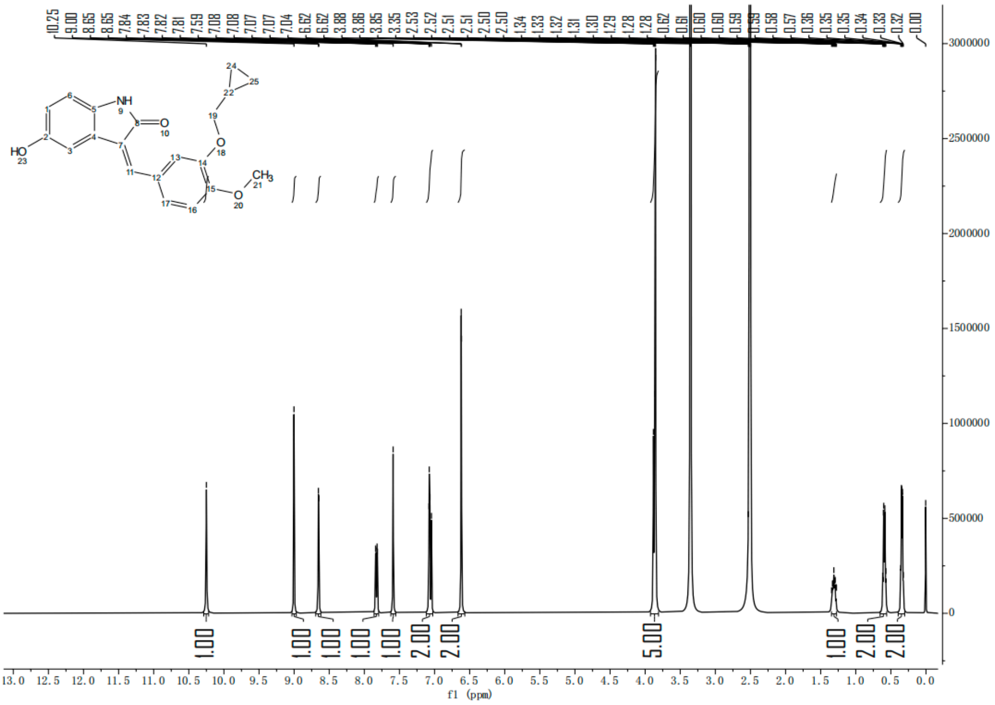

Figure S52.^{1}H-NMR Spectrum of 4d (300 MHz, DMSO-d6)

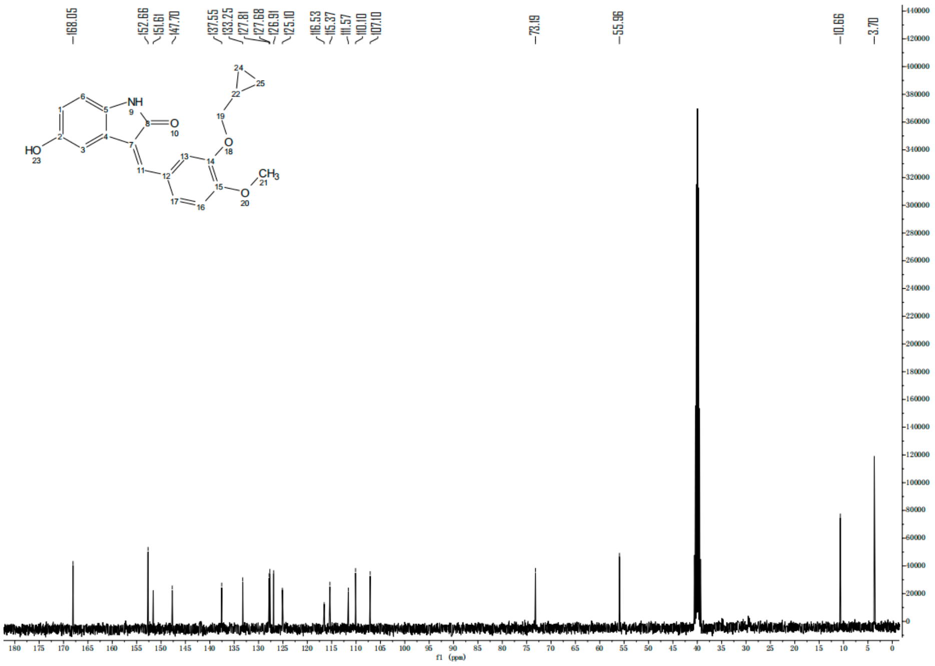

Figure S53.^{13}C-NMR Spectrum of the 4d (101 MHz, DMSO-d6)

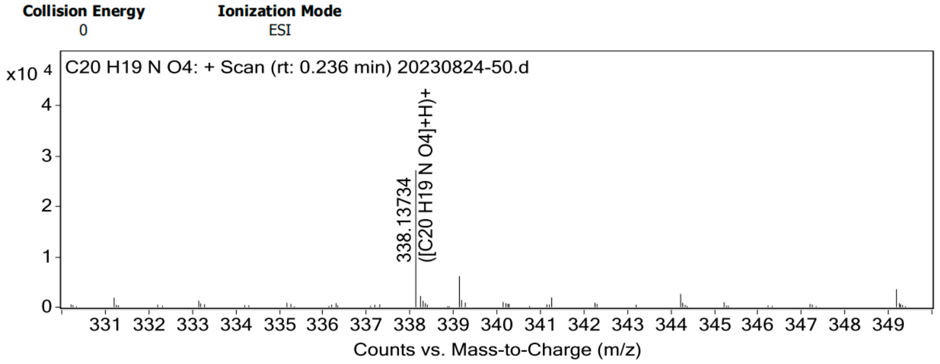

Figure S54.HRMS (ESI) Spectrum of the 4d

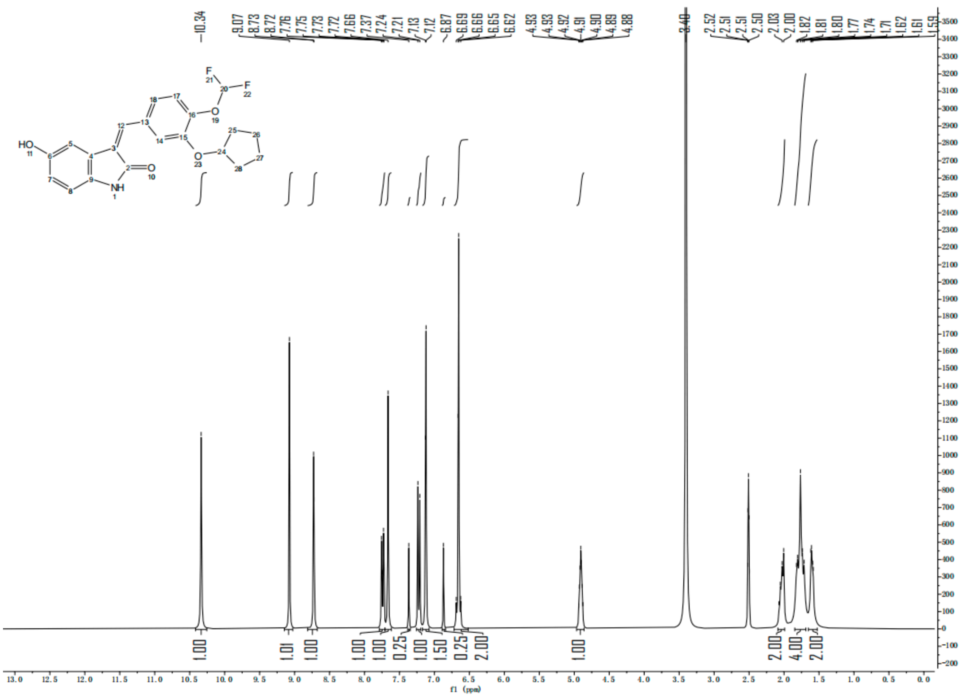

Figure S55.^{1}H-NMR Spectrum of 4e (300 MHz, DMSO-d6)

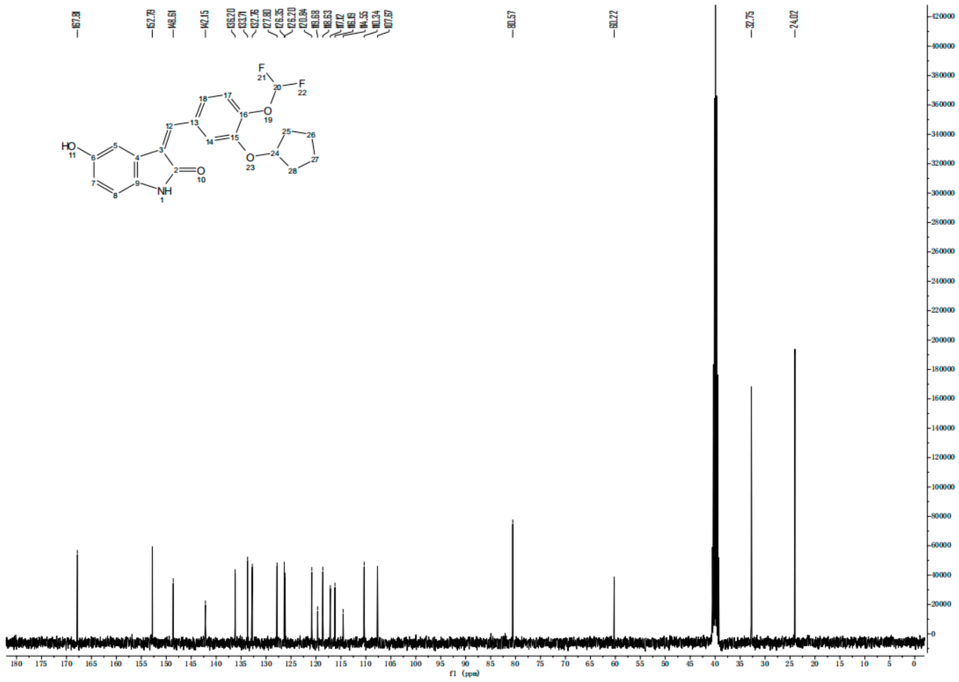

Figure S56.^{13}C-NMR Spectrum of the 4e (101 MHz, DMSO-d6)

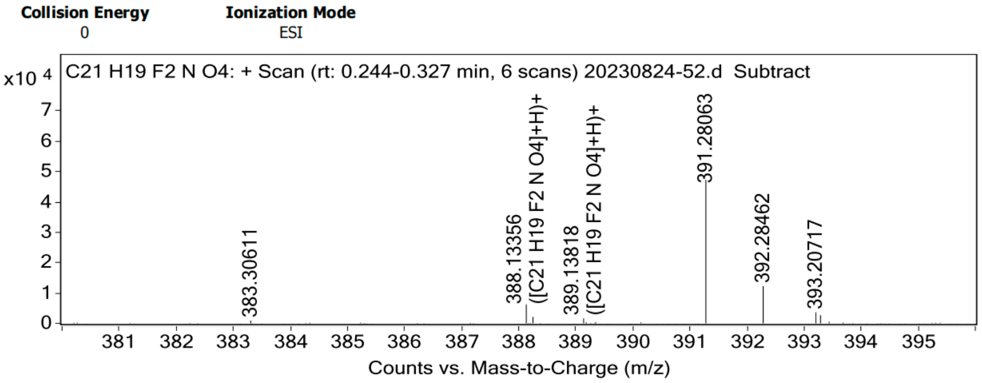

Figure S57.HRMS (ESI) Spectrum of the 4e

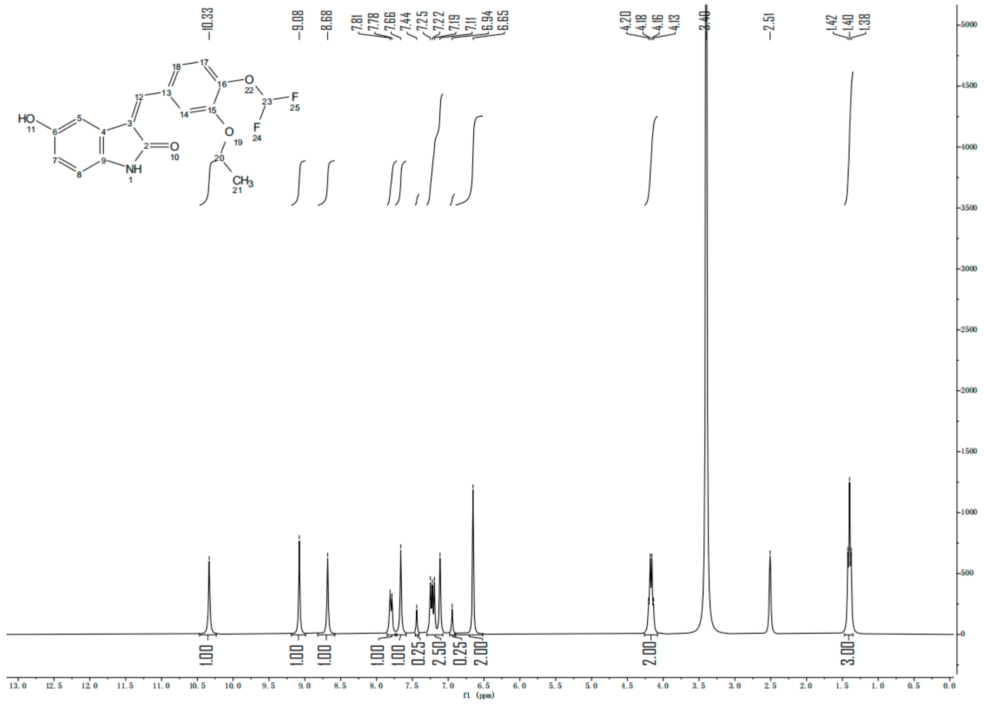

Figure S58.^{1}H-NMR Spectrum of 4f (300 MHz, DMSO-d6)

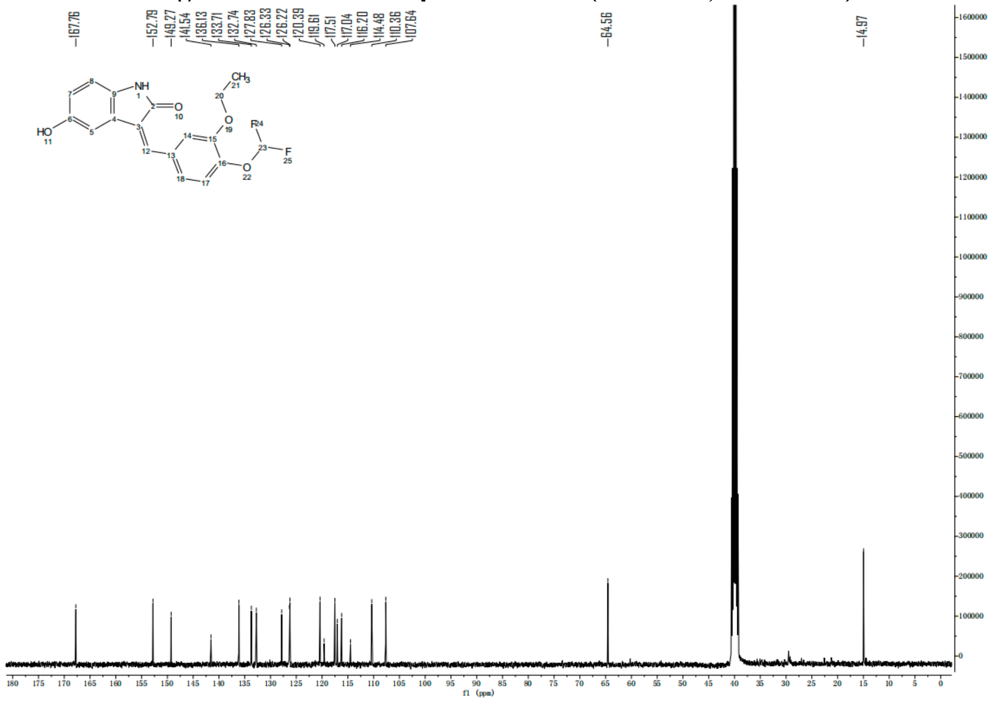

Figure S59.^{13}C-NMR Spectrum of the 4f (101 MHz, DMSO-d6)

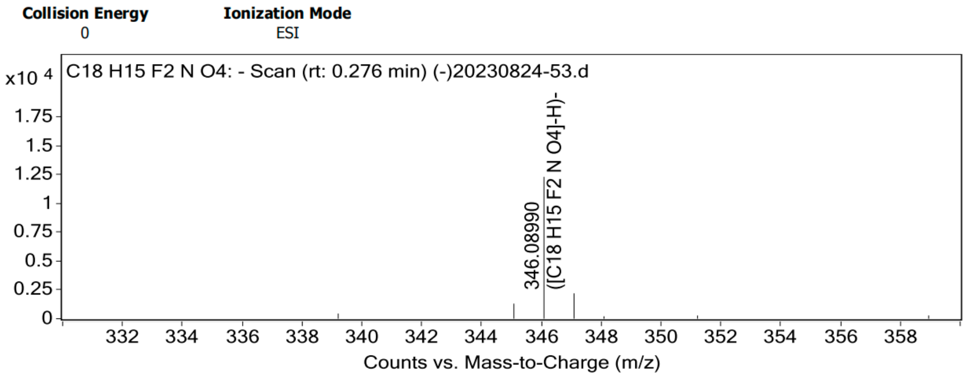

Figure S60.HRMS (ESI) Spectrum of the 4f

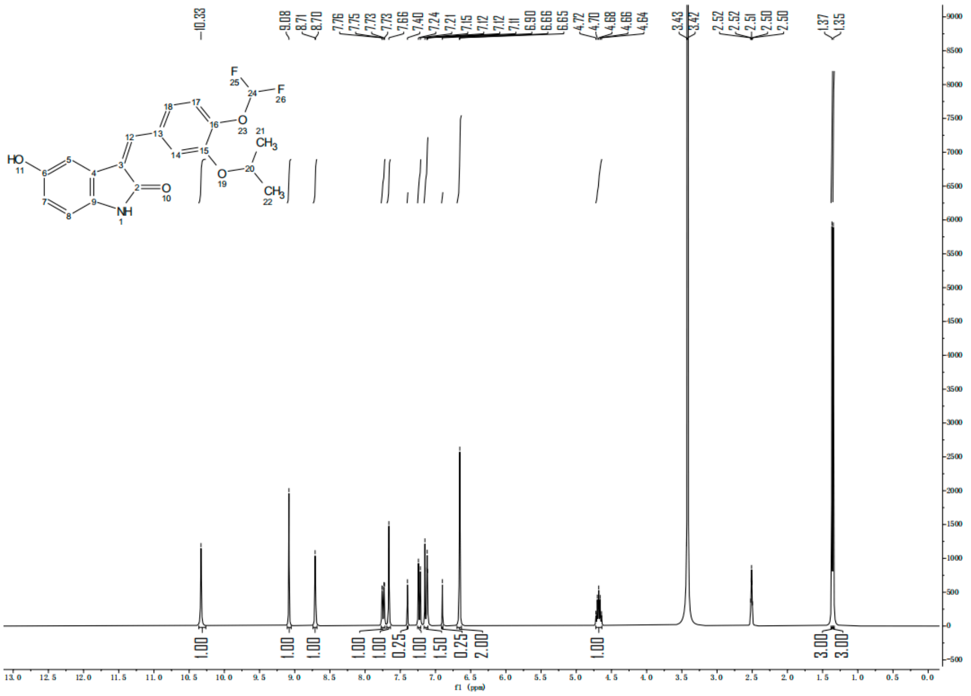

Figure S61.^{1}H-NMR Spectrum of 4g (300 MHz, DMSO-d6)

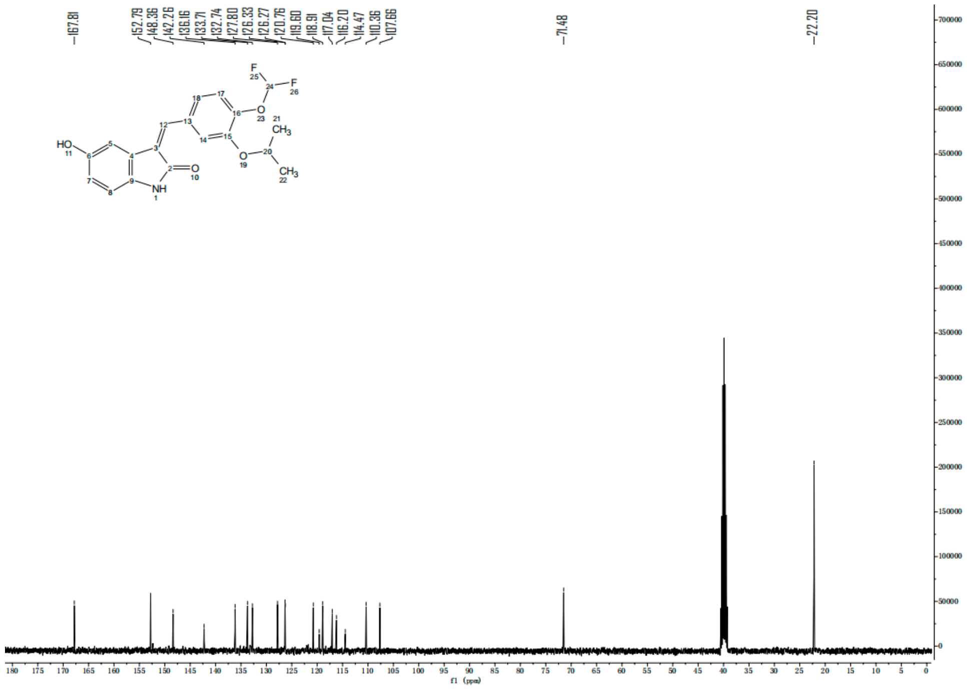

Figure S62.^{13}C-NMR Spectrum of the 4g (101 MHz, DMSO-d6)

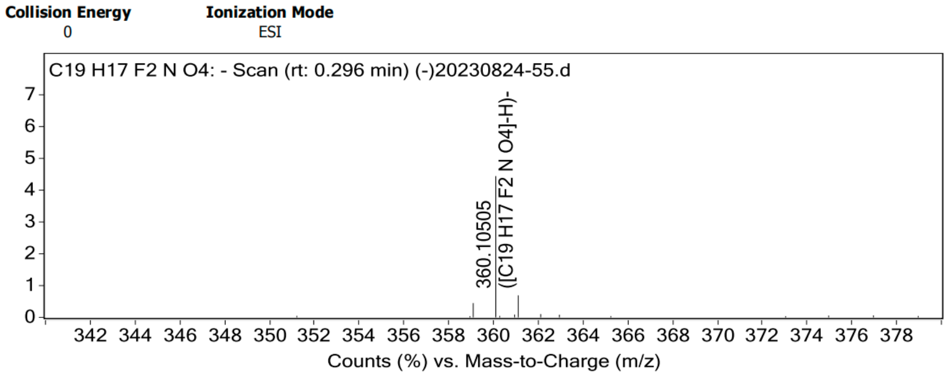

Figure S63.HRMS (ESI) Spectrum of the 4g

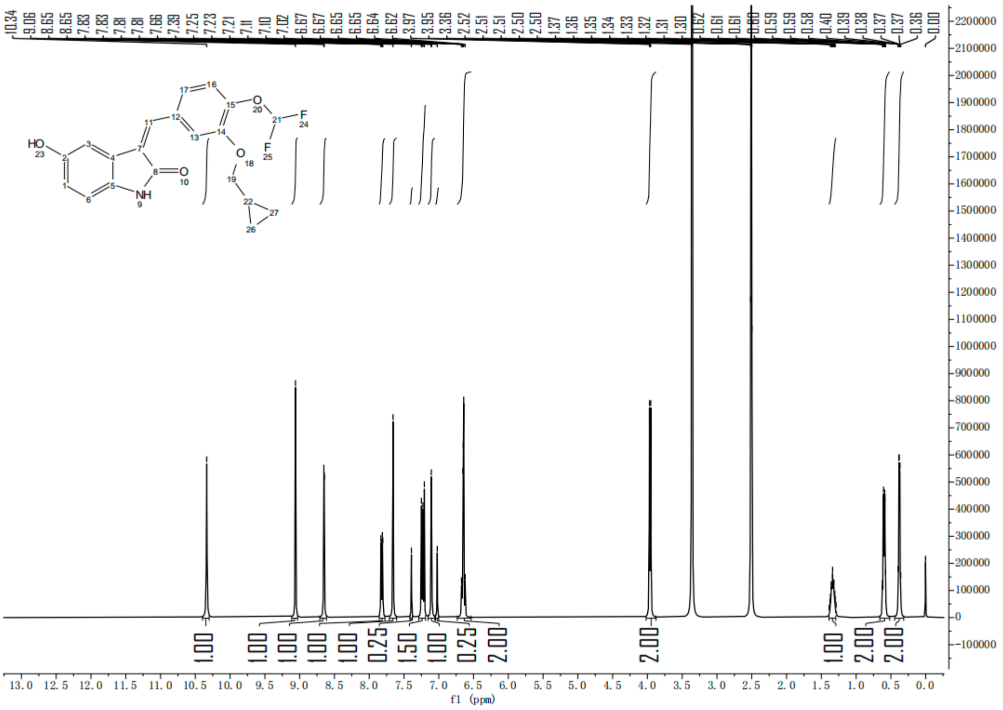

Figure S64.^{1}H-NMR Spectrum of 4h (300 MHz, DMSO-d6)

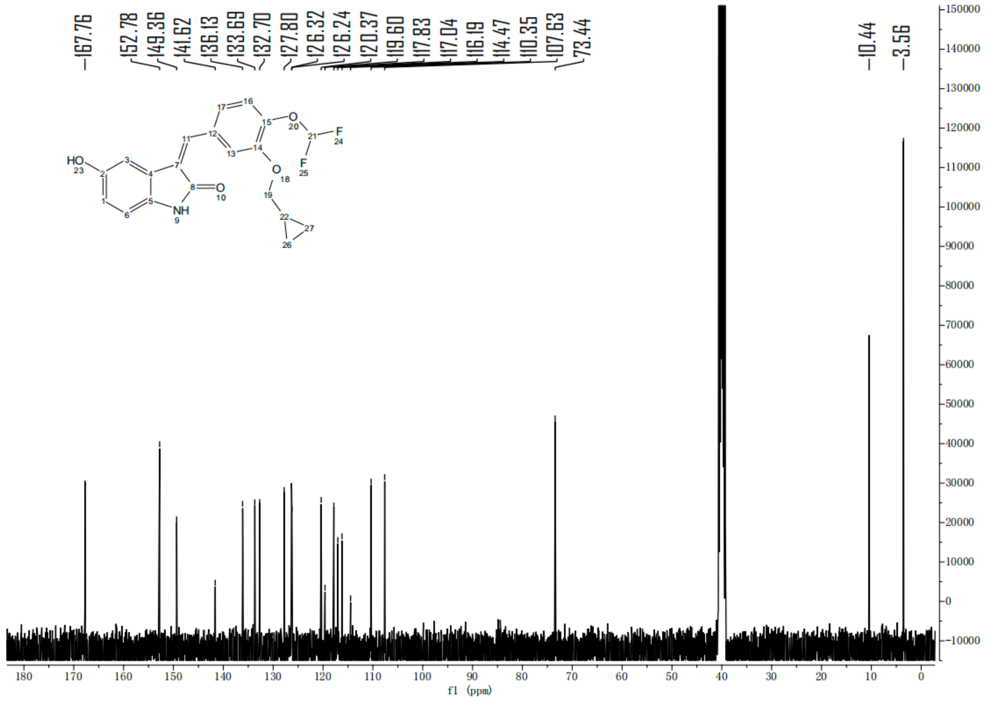

Figure S65.^{13}C-NMR Spectrum of the 4h (101 MHz, DMSO-d6)

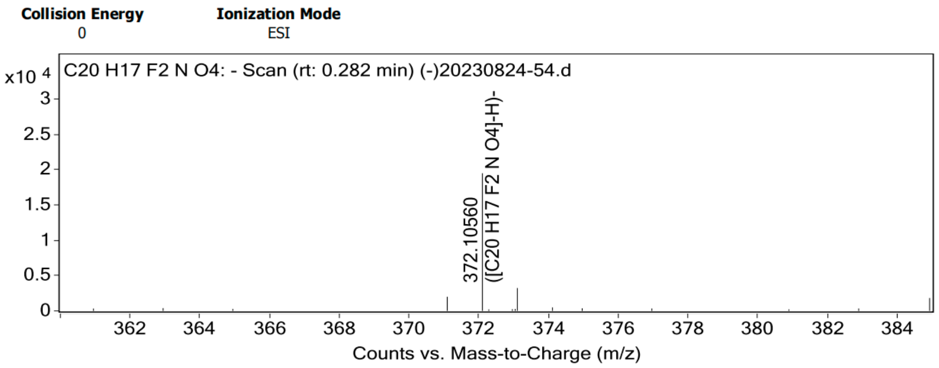

Figure S66.HRMS (ESI) Spectrum of the 4h
